# Nodulation Signaling Pathway 1 and 2 Modulate Vanadium Accumulation and Tolerance of Legumes

**DOI:** 10.1002/advs.202306389

**Published:** 2024-01-15

**Authors:** Peng Liu, Xinfei Zhang, Lin Lin, Yanyan Cao, Xizhen Lin, Liaoliao Ye, Jun Yan, Huiling Gao, Jiangqi Wen, Kirankumar S. Mysore, Jinlong Liu

**Affiliations:** ^1^ College of Grassland Agriculture Northwest A&F University Yangling Shaanxi 712 100 P. R. China; ^2^ State Key Laboratory of Crop Stress Biology for Arid Areas College of Life Sciences Northwest A&F University Yangling Shaanxi 712 100 P. R. China; ^3^ Institute for Agricultural Biosciences Oklahoma State University 3210 Sam Noble Parkway Ardmore OK 73401 USA

**Keywords:** legume, nodule, NSP1, NSP2, rhizobacteria, transporter, vanadium

## Abstract

Vanadium (V) pollution potentially threatens human health. Here, it is found that *nsp1* and *nsp2*, *Rhizobium* symbiosis defective mutants of *Medicago truncatula*, are sensitive to V. Concentrations of phosphorus (P), iron (Fe), and sulfur (S) with V are negatively correlated in the shoots of wild‐type R108, but not in mutant *nsp1* and *nsp2* shoots. Mutations in the P transporter *PHT1*, *PHO1*, and *VPT* families, Fe transporter *IRT1*, and S transporter *SULTR1/3/4* family confer varying degrees of V tolerance on plants. Among these gene families, *MtPT1*, *MtZIP6*, *MtZIP9*, and *MtSULTR1; 1* in R108 roots are significantly inhibited by V stress, while *MtPHO1; 2*, *MtVPT2*, and *MtVPT3* are significantly induced. Overexpression of *Arabidopsis thaliana VPT1* or *M. truncatula MtVPT3* increases plant V tolerance. However, the response of these genes to V is weakened in *nsp1* or *nsp2* and influenced by soil microorganisms. Mutations in *NSPs* reduce rhizobacterial diversity under V stress and simplify the V‐responsive operational taxonomic unit modules in co‐occurrence networks. Furthermore, R108 recruits more beneficial rhizobacteria related to V, P, Fe, and S than does *nsp1* or *nsp2*. Thus, NSPs can modulate the accumulation and tolerance of legumes to V through P, Fe, and S transporters, ion homeostasis, and rhizobacterial community responses.

## Graphical abstract

Vanadium (V) pollution potentially threatens human health through the food chain. This study uncovers the mechanisms of Nodulation Signaling Pathway 1 (NSP1) and NSP2 regulate V accumulation and V tolerance of legumes by regulating the expression of P, S, and Fe transporter genes in a *Rhizobium* symbiosis‐ and rhizosphere microbiome‐dependent manner.

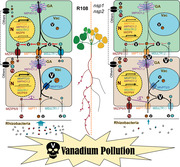

## 1 Introduction

Vanadium (V) is a strategic metal, widely used as a raw material and additive in industries such as aerospace and defense.^[^ ^1^ ^]^ In recent years, owing to extensive V mining and the extraction and combustion of fossil fuels, the amount of V emitted into the environment by humans has far exceeded natural background levels. This has led to increasing V pollution.^[^ ^2^ ^]^ V has several valence states ranging from −1 to +5, of which pentavalent is the most common form in the environment but also the most mobile and toxic.^[^ ^3^ ^]^ V is readily absorbed and accumulated by plants, causing growth retardation, root deformation, yellowing, and wilting.^[^ ^4^ ^]^ In addition, it can readily threaten human health through the food chain, as excessive absorption of it can increase the likelihood of uremia and lung cancer.^[^ ^5^ ^]^ Therefore, more attention must be paid to reducing the absorption and accumulation of V in plants.

As V is similar in structure and electrical charge to phosphate (P), there has been speculation that V may be absorbed by plants through the P uptake system in competition with P.^[^ ^6^ ^]^ Aihemaiti, et al. found that bristlegrass P uptake was significantly reduced under exposure to high levels of V.^[^ ^7^ ^]^ Nawaz, et al. also found similar results in watermelon.^[^ ^8^ ^]^ Furthermore, previous studies have found that the accumulation of V is also related to the accumulation of iron (Fe), sulfur (S), and calcium (Ca).^[^ ^9^ ^]^ P is mainly acquired, translocated, and stored by PHOSPHATE‐TRANSPORTER1 (PHT1), PHOSPHATE 1 (PHO1), and VACUOLAR PHOSPHATE TRANSPORTER (VPT) family members, respectively.^[^ ^10^ ^]^ The ZINC‐IRON PERMEASE (ZIP) family protein IRON‐REGULATED TRANSPORTER 1 (IRT1) is essential for Fe uptake from the soil by plants.^[^ ^11^ ^]^ Members of the SULFATE TRANSPORTER (SULTR) family mediate the absorption and translocation of sulfate (SO_4_^2−^) in higher plants.^[^ ^12^ ^]^ However, there is a lack of genetic evidence supporting that these transporters can regulate V uptake, accumulation, and tolerance in plants so far. Rhizobacteria can detoxify V and reduce its accumulation through valence change, chelation, and promotion of the nutrients that plants uptake.^[^ ^13^ ^]^ Previous research has shown that *Arthrobacter* sp. 5k4‐8‐1 inoculation can convert pentavalent V to tetravalent V, significantly reducing V bioavailability and migration, thereby reducing V toxicity and accumulation in alfalfa.^[^ ^14^ ^]^ Another study has shown that inoculation with *Serratia PRE01* or *Arthrobacter PRE05* can improve V tolerance in *Brassica juncea* by increasing synthesis of antioxidant.^[^ ^15^ ^]^ In the natural environment, however, microorganisms exist as communities, and the abundance and function of individual microorganisms are influenced by other microorganisms, plant species, and their genotypes. Nevertheless, the response, function, and genetic regulation of rhizobacterial communities under V exposure are still unclear.

Legumes are important sources of protein for humans and animals. Reducing the uptake and accumulation of V in legumes is therefore important for global food security. *Rhizobium* symbiosis can not only fix nitrogen, but can also regulate the tolerance and accumulation of heavy metals in legumes.^[^ ^16^ ^]^ Liu et al. showed that *Rhizobium* symbiosis could significantly improve arsenic (As) tolerance of *Medicago truncatula* and reduce the accumulation of As in shoots.^[^ ^17^ ^]^ *Rhizobium* symbiosis has also been reported to improve the tolerance of alfalfa and soybean to cadmium, lead, zinc, and copper.^[^ ^18^ ^]^ However, its role in V uptake and accumulation in legumes is still unclear. In the absence or limitation of molybdenum (Mo), the use of V instead of Mo on the active site of nitrogenase may play a more prominent role in nitrogen fixation.^[^ ^19^ ^]^ Therefore, *Rhizobium*–legume symbiosis has the potential to absorb and utilize V as a beneficial element, but at the same time, it is possible that V causes damage to other organs. Thus, *Rhizobium* symbiosis may improve V tolerance in legumes and regulate V distribution and accumulation in plants to minimize V toxicity. To date, many genes regulating *Rhizobium* symbiosis have been identified, including *NODULATION SIGNALING PATHWAY 1* (*NSP1*), *NSP2*, *NODULE INCEPTION* (*NIN)* and *AURORA KINASE 1* (*AUR1*), but whether they can regulate V uptake and tolerance in legumes remains unknown.^[^ ^20^ ^]^

With above issues in mind, we analyzed the differences in V tolerance and accumulation between *M. truncatula* wild‐type R108 and the *Rhizobium* symbiosis defective mutants *nsp1* and *nsp2*. Furthermore, the relationship between V and the accumulation of P, Fe, S, and Ca was analyzed. Then, targeting the nutrient elements more associated with V accumulation, the role of various nutrients in regulating V tolerance was genetically confirmed by screening a series of related mutants and measuring the expression of related genes. Finally, we analyzed the diversity of rhizobacteria, inferred co‐occurrence networks, and performed a correlation analysis between rhizobacteria and element concentrations in plants. The aim was to elucidate the molecular mechanism by which NSP1 and NSP2 mediate *Rhizobium* symbiosis regulating V tolerance in legumes from the levels of genetic and rhizosphere microbial regulation and to provide new ideas for environmentally sustainable management of V pollution and human health.

## 2 Results

### 2.1 *NSP1* and *NSP2* Mutations Affect Plant Tolerance and Accumulation of V

**Figure 1:**
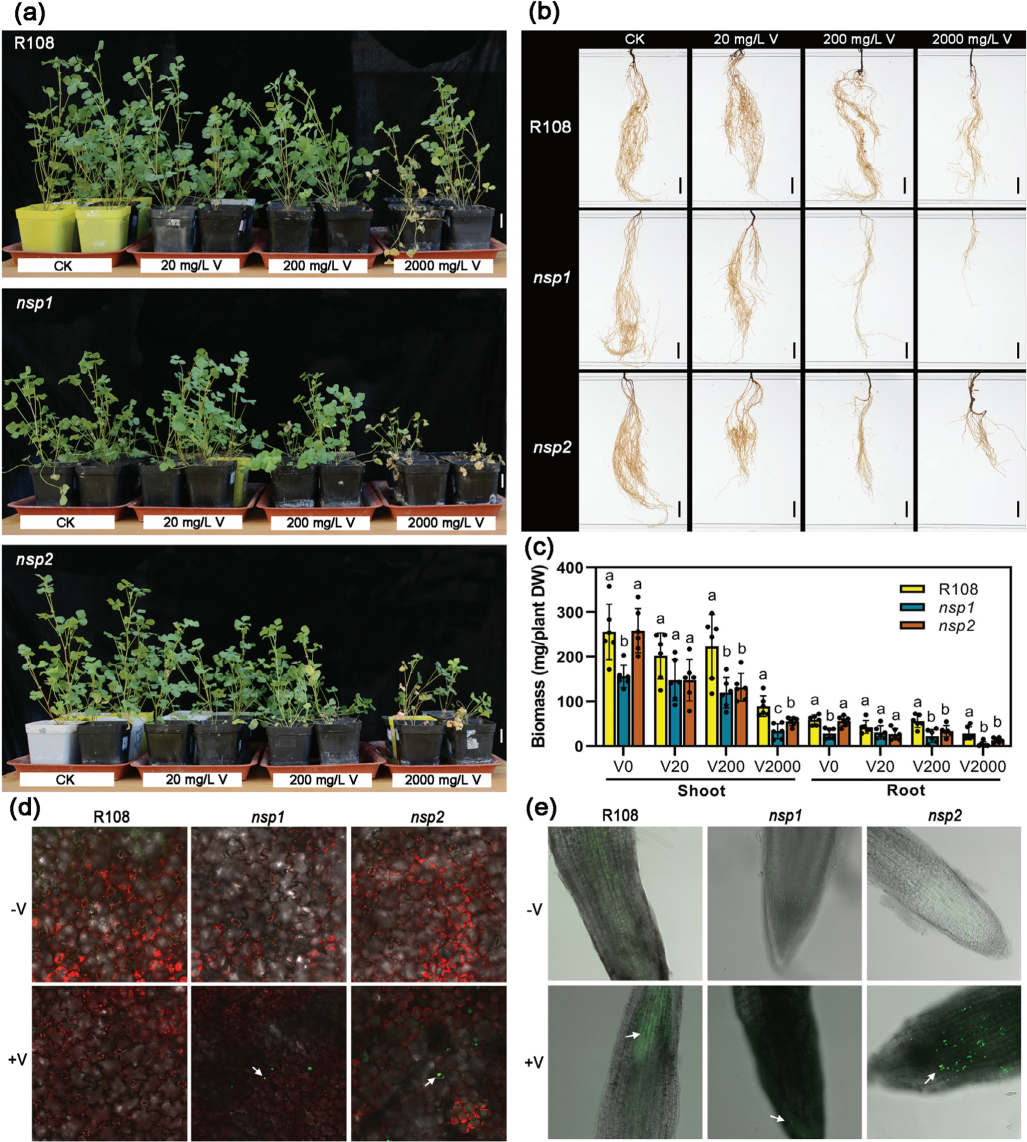
*NSP1* and *NSP2* mutations increase vanadium (V) sensitivity. a,b) Shoot (a) and root (b) phenotypes of wild‐type (R108) and mutant *nsp1* and *nsp2 Medicago truncatula* plants under different V conditions. Four‐week‐old seedlings were treated with the indicated concentrations of V for one week. Bars, 2 cm. c) Biomass of R108, *nsp1*, and *nsp2* plants under different V conditions. Plants were treated as described in (a) and (b). The treatment concentrations of V were 0 mg L^−1^ (V0), 20 mg L^−1^ (V20), 200 mg L^−1^ (V200), and 2000 mg L^−1^ (V2000). Data are expressed as mean ± SD values (*n* = 6). Different letters above the bars indicate significant differences at *P* < 0.05 (Duncan's test). d,e) Intracellular ROS in the leaves (d) and roots (e) of R108, *nsp1*, and *nsp2* plants detected using 2′−7′ dichlorodihydrofluorescein diacetate (H_2_DCFDA). Three‐week‐old seedlings were cultured with ½‐strength Hoagland nutrient solution prepared with soil extract. After nodulation of R108, plants were treated with (+V) or without (‐V) 100 mg L^−1^ V for 2 days. Then, leaves and roots were obtained for ROS detection. Arrows indicate where ROS accumulated in tissues.

To investigate the effects of *Rhizobium* symbiosis mediated by *NSP1* and *NSP2* mutations on V accumulation and tolerance in legumes, a gradient of V treatments (0, 20, 200, and 2000 mg L^−1^) was applied to wild‐type R108 and mutant *nsp1* and *nsp2 M. truncatula* plants using natural soil as substrate and a pot control experiment (**Figure** **1**; Figure S1, Supporting Information). A high concentration of V (2000 mg L^−1^) resulted in plant death. However, the condition of shoots and roots of R108 was significantly better than that of *nsp1* and *nsp2* (Figure 1a,b). There was no significant difference in biomass between R108, *nsp1*, and *nsp2* under low V treatment (Figure 1c). However, the shoot and root biomass of *nsp1* and *nsp2* was significantly lower than that of R108 when the concentration of the V treatment was greater than 200 mg L^−1^. Further, we detected reactive oxygen species (ROS) in tissues by confocal microscopy using H_2_DCFDA (2,7‐Dichlorodihydrofluorescein diacetate). The results showed that short‐term stress of 100 mg L^−1^ V was more likely to cause ROS accumulation in *nsp1* and *nsp2* than wild‐type R108 (Figure 1d,e). In addition, we analyzed the effects of V stress on photosynthesis by chlorophyll fluorescence parameters F_0_, F_m_ and F_v_/F_m_ ratios (Figure S2, Supporting Information). The results of F_v_/F_m_ ratios showed that short‐term V stress had no significant effect on the photosynthetic efficiency (Figure S2c,f, Supporting Information). However, unlike R108, F_m_ in *nsp1* and *nsp2* decreased significantly under V stress, indicating that electron transport in photosystem II (PS II) of *nsp1* and *nsp2* was significantly hindered (Figure S2b,e, Supporting Information). These results fully indicated that mutations in *NSP1* and *NSP2* reduce plant tolerance to V.

**Figure 2:**
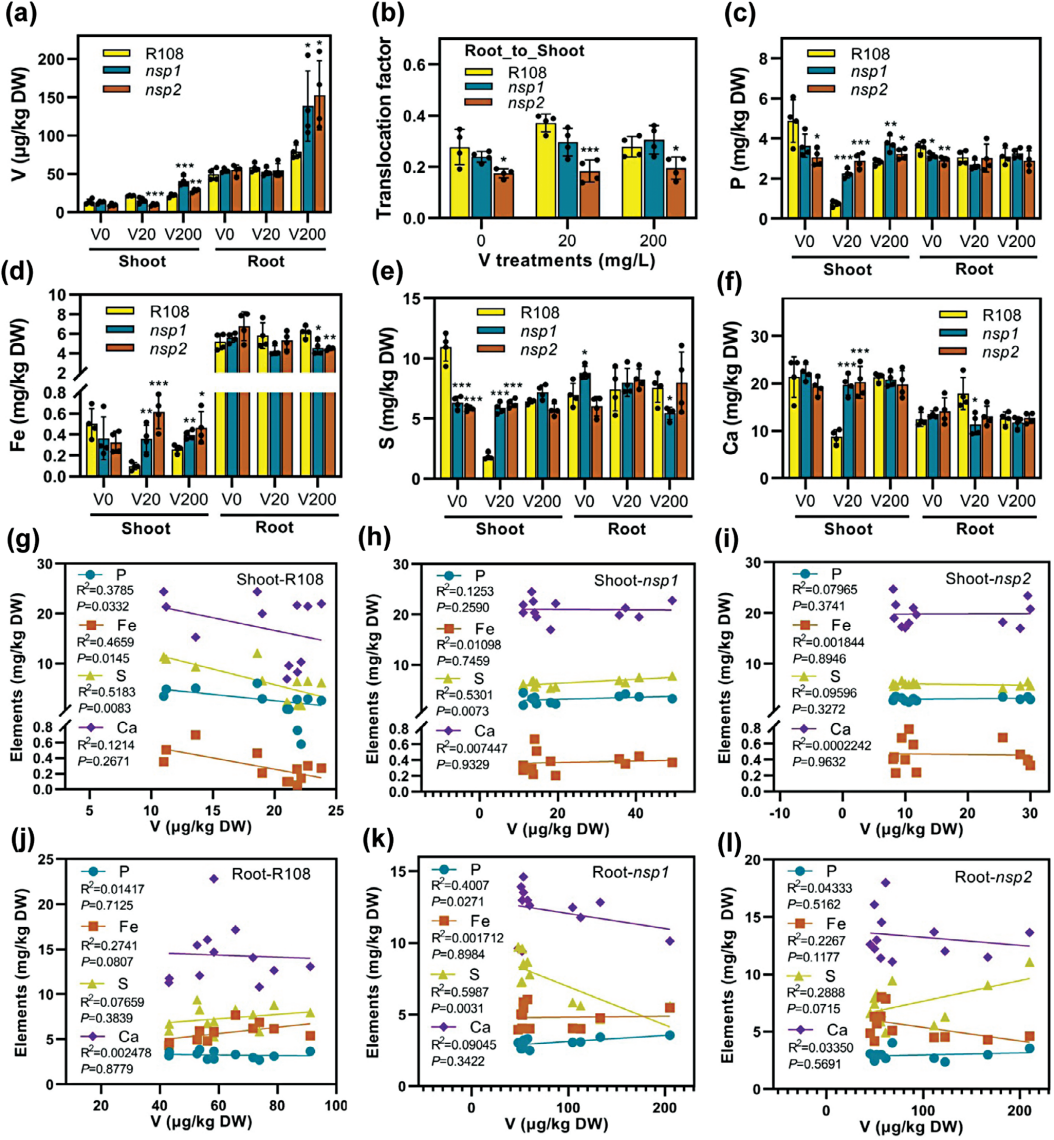
*NSP1* and *NSP2* mutations alter the accumulation of vanadium (V) and the correlations of V concentration with phosphorus (P), iron (Fe), sulfur (S), and calcium (Ca) concentrations. a) The V concentration in the shoots and roots of 5‐week‐old wild‐type (R108), *nsp1*, and *nsp2 Medicago truncatula* plants treated with different concentrations of V for one week. The treatment concentrations of V were 0 mg L^−1^ (V0), 20 mg L^−1^ (V20), and 200 mg L^−1^ (V200). b) The translocation factor of V from roots to shoots of R108, *nsp1*, and *nsp2* plants. Data were calculated from shoot‐to‐root ratios of V concentrations in (a). c–f) Concentrations of P, Fe, S, and Ca in shoots and roots of wild‐type (R108) and mutant *nsp1* and *nsp2* plants described in (a). Data in (a–f) are expressed as mean ± SD values (*n* = 4). Statistical significance is denoted by asterisks based on independent sample *t*‐tests (* *P* < 0.05, ** *P* < 0.01, *** *P* < 0.001). g–i) Correlation analysis of the V concentration with P, Fe, S, and Ca in shoots of wild‐type (R108) (g), *nsp1* (h), and *nsp2* (i) and roots of j) R108, k) *nsp1*, and l) *nsp2* using the data in (a) and (c–f).

Owing to the death of some plants treated with 2000 mg L^−1^ V and the inability to accurately measure the physical and chemical indicators, we measured the V concentrations in plants treated with 0, 20, and 200 mg L^−1^ Na_3_VO_4_ (**Figure** **2**). There was no significant difference in V concentrations among R108, *nsp1*, and *nsp2* roots when the treatment concentration was 20 mg L^−1^ (Figure 2a). However, the V concentration in R108 shoots increased significantly and was significantly higher than that in *nsp1* and *nsp2* shoots. Additionally, *nsp1* and *nsp2* both showed a more pronounced V‐sensitive phenotype than did R108, as the V concentrations in the shoots and roots were significantly higher than that in R108 when the V treatment concentration reached 200 mg L^−1^ (Figures 1 and 2a). The transport coefficient showed that the efficiency of V transport from root to shoot in *nsp1* and *nsp2* was lower than that in R108, especially in *nsp2* (Figure 2b). These results indicated that the absorption and accumulation of V in *M. truncatula* may be affected by *NSP1* and *NSP2* mutations.

### 2.2 *NSP1* and *NSP2* Mutations Weaken or Alter Correlated Concentrations of P, Fe, and S with V in Plants

The accumulation and tolerance of V in plants are closely related to P, Fe, S, and Ca. To investigate the change in V absorption and accumulation caused by *NSP1* and *NSP2*, the levels of P, Fe, S, and Ca were also determined. Overall, under V stress, the differences in P and Fe concentrations between R108, *nsp1*, and *nsp*2 were most significant, followed by differences in S and Ca (Figure 2c–f). Moreover, the differences between these elements were mainly reflected in shoots. Under normal conditions, shoot levels of P, Fe, and S were higher in R108 than in *nsp1* and *nsp2*, especially P and S (Figure 2c–e). However, V treatment at 20 mg L^−1^ resulted in a significant decrease in P, Fe, S, and Ca concentrations in the shoots of R108, but the effects on *nsp1* and *nsp2* were relatively weak or even opposite (Figure 2c–f). Therefore, the concentrations of P, Fe, S, and Ca in R108 shoots were significantly lower than those in *nsp1* and *nsp2* shoots. Although the concentrations of P, Fe, S, and Ca in R108 shoots increased under 200 mg L^−1^ V treatment, the concentrations of P and Fe in R108 shoots were still significantly lower than those in *nsp1* and *nsp2* shoots. Compared with 20 mg L^−1^ V treatment, 200 mg L^−1^ V treatment significantly increased the P/V ratio, Fe/V ratio, S/V ratio and Ca/V ratio in R108 (Figure S3, Supporting Information). However, this trend was reversed in *nsp1* and *nsp2*. The P/V ratio, S/V ratio and Ca/V ratio in R108 were higher than those in *nsp1* and *nsp2* under 200 mg L^−1^ V treatment. Correlation analysis showed that V concentrations in R108 shoots were significantly negatively correlated with P, Fe, and S concentrations, but not significantly correlated with Ca concentration (Figure 2g). Mutations in *NSP1* and *NSP2* attenuated these associations and even were linked with the opposite result to that observed in R108 (Figure 2g–i). However, there was no significant correlation in R108 roots between V concentrations and P, Fe, S, and Ca concentrations (Figure 2j). Although the correlation in *nsp2* roots was also not significant, the mutation of *NSP1* significantly increased the correlations of V concentration with P and S concentrations in roots (Figure 2k,l). However, the trend was opposite to that observed in R108 (Figure 2j,k). Thus, *NSP1* and *NSP2* mutations disrupt the response of P, Fe and S to V accumulation in plants.

### 2.3 The V Sensitivity and Element Accumulation of *nsp1* and *nsp2* Mutants is Affected by P, Fe, and S Conditions

**Figure 3:**
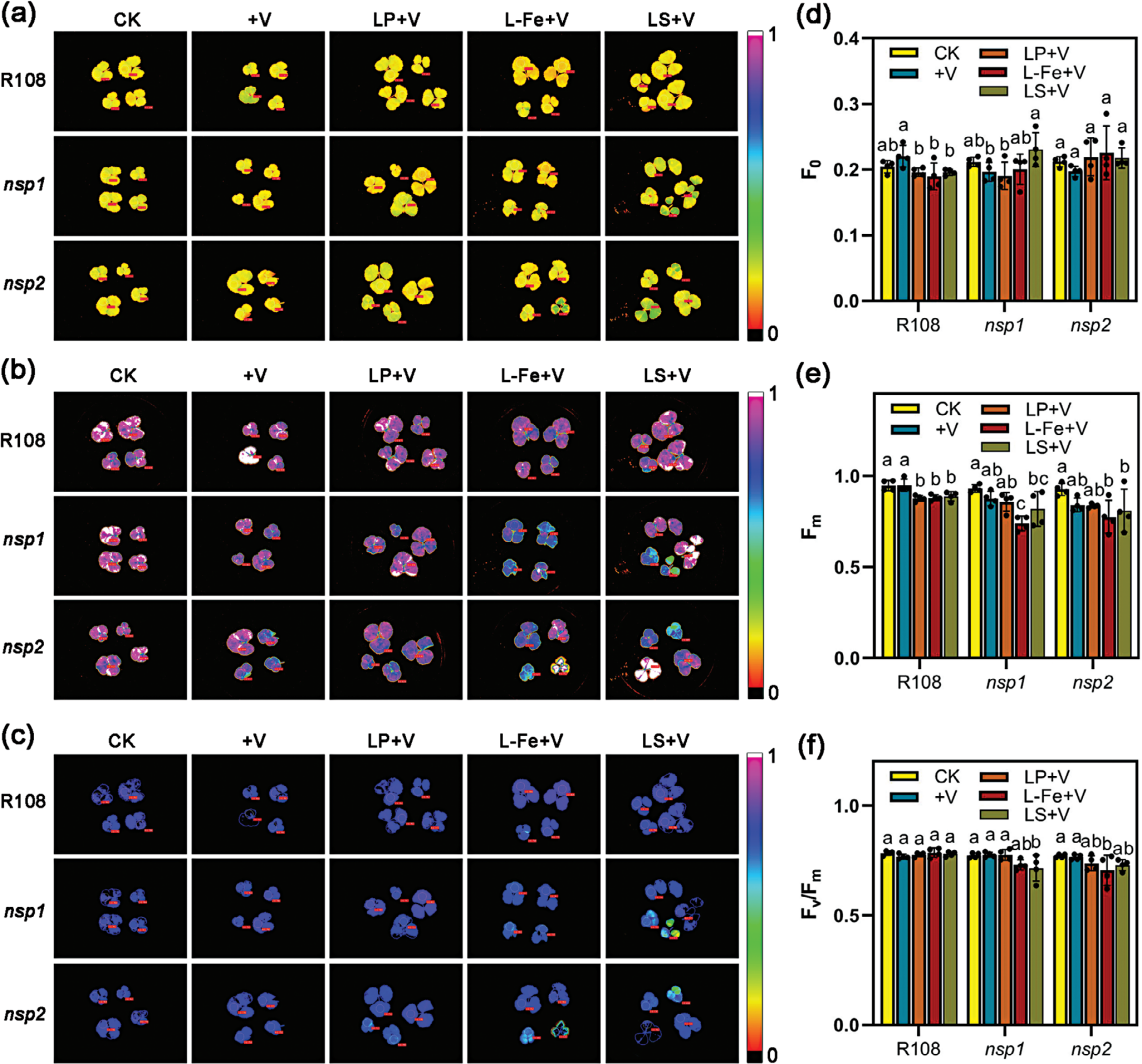
The deficiency of phosphorus (P), iron (Fe), or sulfur (S) increases the sensitivity of plants to vanadium (V) stress. a–c) Images of F0 (a), Fm (b), and Fv/Fm ratios (c) in the leaves of wild type (R108) and mutant *nsp1* and *nsp2 Medicago truncatula* plants. Three‐week‐old seedlings were cultured with ½‐strength Hoagland nutrient solution prepared with soil extract. After nodulation of R108, plants were treated with 100 mg L^−1^ V (+V) under low P (LP, 1 µm PO_4_^3−^), low Fe (L‐Fe, 1 µm Fe^2+^), or low S (LS, 1 µm SO_4_^2−^) conditions for 16 h. Then, leaves were obtained for chlorophyll fluorescence imaging. d–f) Statistical analysis of F0 (d), Fm (e), and Fv/Fm ratios (f) in (a‐c). Data are expressed as mean ± SD values (*n* = 4). Different letters above the bars indicate significant differences at *P* < 0.05 (Duncan's test).

In order to clarify the regulatory effects of P, Fe, and S on V accumulation and V tolerance in plants, we investigated the effects of various P, Fe, and conditions on V sensitivity and element accumulation in R108, *nsp1*, and *nsp2*. Chlorophyll fluorescence analysis was performed on plant leaves after 16 hours of short‐term V treatment with low P, Fe, or S (**Figure** **3**). The results showed that the F_m_ of wild‐type R108 under V stress was significantly reduced by low P, Fe or S treatment, indicating that the electron transport of PS II was significantly impaired (Figure 3b,e). Importantly, Fe or S deficiency further exacerbated the inhibition of F_m_ in *nsp1* and *nsp2* by V stress. Low P, Fe, or S treatments had little effect on F_v_/F_m_ ratios in R108 (Figure 3c,f). However, the F_v_/F_m_ ratios in *nsp1* and *nsp2* under V stress was reduced to varying degrees by low Fe or low S treatment, indicating a decrease in photosynthetic efficiency. These results indicate that the deficiency of P, Fe, or S can increase the sensitivity of plant photosynthetic apparatus to V stress, while *nsp1* and *nsp2* are more sensitive to the deficiency of Fe or S.

Further, we analyzed the effects of long‐term treatment with different concentrations of P, S or Fe on V sensitivity of R108, *nsp1*, and *nsp2*. In addition, DAB (3,3‐diaminobenzidine) staining was performed to analyze ROS production. According to the case of leaf death and tissue ROS accumulation, reducing the concentration of P in the environment significantly increased the sensitivity of plants to V stress, especially the wild‐type R108 (Figures S4a and S5, Supporting Information). On the contrary, plant V tolerance was stronger in high P conditions. The phenotypes of *nsp1* and *nsp2* that were more sensitive to V than R108 were more likely to appear under high P conditions. However, the growth status of *nsp1* and *nsp2* was not worse than that of R108 under high P condition without V (Figure S6, Supporting Information). The V concentration in shoots of *nsp1* and *nsp2* was higher than that of R108 under both high and low P conditions (Figure S4b, Supporting Information). Interestingly, the change of total P level was very consistent with that of V (Figure S4b,c, Supporting Information). High P conditions significantly increased the P/V ratio of wild‐type R108, but had no significant effect on *nsp1* and *nsp2* (Figure S4d, Supporting Information). Thus, the P/V ratio may explain changes in plant tolerance better than either P or V alone.

Similar to P deficiency, Fe deficiency also increased the degree of V stress to plants (Figure S7a, Supporting Information). According to the case of leaf death and tissue ROS accumulation, the V‐sensitive phenotypes of *nsp1* and *nsp2* were more prominent under low Fe conditions than under high Fe conditions (Figures S7a and S8, Supporting Information). However, the leaf yellowing degree of nsp1 and nsp2 was not more serious than that of R108 under low Fe condition without V (Figure S9, Supporting Information). The translocation factor of V from root to shoot was significantly increased in low Fe conditions, and the level of V in shoot was increased, thus aggravating the symptoms of V toxicity (Figure S7b,c, Supporting Information). This was more prominent in *nsp1* and *nsp2* than in R108. Under high Fe conditions, V was more likely to accumulate in the root, thus reducing its transport to the shoot. Except in the shoot of *nsp2*, the variation trend of P concentration in plants was very similar to that of V (Figure S7b,d, Supporting Information). Interestingly, under Fe deficiency conditions, although the Fe level in the root decreased significantly, the Fe level in the shoot increased significantly (Figure S7e, Supporting Information). Compared with *nsp1* and *nsp2*, the Fe/V ratio in shoots of R108 was higher and further increased under the condition of low Fe (Figure S7f, Supporting Information). However, the Fe/V ratio in *nsp1* and *nsp2* did not respond significantly to the low Fe condition. In addition, under low Fe conditions, the P/V ratio in the shoot of the wild‐type plants did not change significantly, but decreased significantly in that of *nsp1* and *nsp2*. Therefore, the response defects of Fe/V and P/V ratio may be important reasons for the increased sensitivity of *nsp1* and *nsp2* to V caused by low Fe.

Low S conditions also significantly increased the sensitivity of plants to V stress, especially *nsp1* mutants (Figure S10a, Supporting Information). Under the condition of low S, although the shoot status of *nsp2* did not change significantly after 5 days of V treatment, a large amount of ROS actually accumulated in tissues (Figures S10a and S11, Supporting Information). The V‐sensitive phenotypes of *nsp1* and *nsp2* were more likely to appear under low S conditions. However, the shoot growth status of *nsp1* and *nsp2* was not significantly different from that of R108 under low S condition without V (Figure S12, Supporting Information). The shoot V concentration of *nsp1* and *nsp2* was higher than that of R108 under both low and high S conditions (Figure S10b, Supporting Information). However, this difference was significantly reduced under high S conditions. Furthermore, the S level in *nsp1* and *nsp2* was significantly higher than that in R108 under high S conditions (Figure S10c, Supporting Information). Moreover, although the S/V ratio in R108 increases significantly under high S conditions, the increase was even greater in *nsp1* and *nsp2* (Figure S10d, Supporting Information). Therefore, high S conditions can improve the V tolerance of plants, but narrow the difference between R108 and *nsp1* and *nsp2*.

### 2.4 Plant V Tolerance is Regulated by P, Fe, and S Transporters

**Figure 4:**
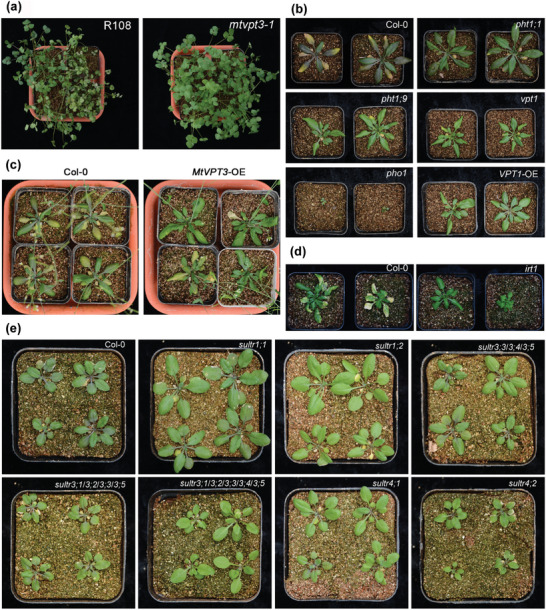
Mutation or overexpression of phosphate (P), iron (Fe) and sulfate (S) transporter genes can enhance plant tolerance to vanadium (V). a) Growth phenotypes of *Medicago truncatula* wild‐type (R108) plants and the vacuolar phosphate transporter mutant *mtvpt3‐1* plants under V stress. Five‐week‐old seedlings were treated with 1000 mg L^−1^ V for five days. b) Growth phenotypes of *Arabidopsis thaliana* wild‐type (Col‐0), P transporter mutant (*pht1;1*, *pht1;9*, *vpt1*, and *pho1*), and *VPT1*‐overexpressing plants (*VPT1‐*OE) under V stress. Three‐week‐old seedlings were treated with 1000 mg L^−1^ V for 10 days. c) Growth phenotypes of wild‐type (Col‐0) and *MtVPT3*‐overexpressing plants (*MtVPT3‐*OE) under V stress. Three‐week‐old seedlings were treated with 1000 mg L^−1^ V for 10 days. d) Growth phenotypes of wild‐type (Col‐0) and Fe transporter mutant *irt1* plants under V stress. Three‐week‐old seedlings were treated with 1000 mg L^−1^ V for 10 days. e) Growth phenotypes of wild‐type (Col‐0) and S transporter mutant (*sultr1;1*, *sultr1;2*, *sultr3;3/3;4/3;5*, *sultr3;1/3;2/3;3/3;5*, *sultr3;1/3;2/3;3/3;4/3;5*, *sultr4;1*, and *sultr4;2*) plants under V stress. Three‐week‐old seedlings were treated with 1000 mg L^−1^ V for one week.

We investigated the regulatory effects of P, Fe, and S transporters on plant V tolerance from a genetic perspective using a series of *M*. *truncatula* and *A. thaliana* mutants (**Figure** **4**). Under the stress of high V (1000 mg L^−1^), *M*. *truncatula* wild‐type R108 showed severe leaf‐wilting phenotypes earlier than did the vacuolar phosphate transporter mutant *mtvpt3‐1* (Figure 4a). In addition, based on the degree of leaves yellowing and turning purple, the *A. thaliana* P transporter mutants *pht1;1*, *pht1;9*, *vpt1*, and *pho1*, Fe transporter mutant *irt1* and S transporters mutants *sultr1;1*, *sultr1;2*, *sultr3;3/3;4/3;5*, *sultr3;1/3;2/3;3/3;5*, *sultr3;1/3;2/3;3/3;4/3;5*, *sultr4;1*, and *sultr4;2* all showed varying degrees of V tolerance phenotypes compared to wild‐type plants (Figure 4b–e). Among them, *pht1;9*, *vpt1*, *irt1*, and *sultr1;2* were the most obvious. Notably, the overexpression of *VPT1* (*A. thaliana*) and *MtVPT3* (*M. truncatula*) can also improve the V tolerance of plants like their mutations (Figure 4b,c). However, under normal conditions, there was no significant difference in leaf yellowing in Col‐0, mutants, and overexpression plants (Figure S13, Supporting Information).

Further, we analyzed ion accumulations in the *mtvpt3* mutant and *MtVPT3* overexpressing plants under V stress. The results showed that *mtvpt3* was more tolerant to V than wild‐type R108, but it had a higher concentration of V accumulated in the shoot (Figure S14a–c, Supporting Information). However, the concentrations of total P and inorganic phosphate (Pi) in the shoot of *mtvpt3* were lower than those of R108 (Figure S14d,e, Supporting Information). Unexpectedly, the total S and total Ca levels in the shoot of *mtvpt3* were significantly higher than those of wild‐type plants, which may be related to the V tolerance of *mtvpt3* (Figure S14d, Supporting Information). The concentrations of V and P in *mtvpt3* root were both significantly lower than that of wild‐type R108 (Figure S14f,g, Supporting Information). However, *mtvpt3* had significantly higher P/V ratio than the wild type (Figure S14h, Supporting Information). Also, the levels of S, Fe and Ca in *mtvpt3* root were significantly higher than those in wild‐type R108 (Figure S14g, Supporting Information). These may also confer *mtvpt3* greater V tolerance. Interestingly, overexpression of *MtVPT3* also significantly increased V levels in plant shoots (Figure S14i,j, Supporting Information). Different from the *mtvpt3* mutant, the total P level in the shoots of *MtVPT3* overexpressed plants was significantly higher than that of the wild type (Figure S14k, Supporting Information). This indicated that the mechanism of V level increase in shoots of *MtVPT3* overexpression plants and *mtvpt3* mutants may be different. Interestingly, changes in S, Fe, and Ca levels in shoots of *MtVPT3* overexpressed plants were very similar to those in *mtvpt3* mutants (Figure S14d,k, Supporting Information). This indicated that *MtVPT3* overexpressed plants and *mtvpt3* mutant plants may have similar mechanisms to cope with V toxicity after accumulating more V. Fe in plants affects the mobility of Pi, and the tolerance of *irt1* may be related to changes in Pi mobility. Our results showed that under normal conditions, the Pi concentration of *irt1* was significantly higher than that of Col‐0, but under V treatment, the Pi concentration of *irt1* was significantly lower than that of Col‐0 (Figure S15, Supporting Information).

Owing to the inability to observe roots in potted vermiculite culture, we analyzed the effect of V stress on plant roots grown in *Arabidopsis* nutrient solution (ANS) agar medium.^[^ ^17^ ^]^ Unexpectedly, the root phenotypes of most mutants under V stress were not significantly different from those of Col‐0 (Figures S16–S19, Supporting Information). However, the roots of *vpt1* and *sultr4;2* were more sensitive to V than those of Col‐0 (Figures S16 and S19a,b, Supporting Information). Reducing the P concentration in the culture medium significantly increased the sensitivity of *A. thaliana* to V, but also reduced the difference in sensitivity between *vpt1* and Col‐0 (Figure S17, Supporting Information). Increasing the Fe concentration in the culture medium was able to significantly alleviate the toxicity of V to Col‐0, but had little effect on *irt1* (Figure S18, Supporting Information). The toxicity of V to *A. thaliana* could also be alleviated by increasing the concentration of SO_4_^2−^ in the medium. The roots of *sultr1;2* showed a significantly more V‐tolerant phenotype than did Col‐0 under a low SO_4_^2−^ concentration of 50 µm, but they were more sensitive than Col‐0 roots under a high SO_4_^2−^ concentration of 5000 µm (Figure S19c,d, Supporting Information). These results indicated that plant V tolerance is regulated by P, Fe, and S transporters, and the levels of P and Fe in the environment determine plant V tolerance.

### 2.5 *NSP1* and *NSP2* Mutations Weaken or Alter the Response of P, Fe, and S Transporter Genes to V Stress

**Figure 5:**
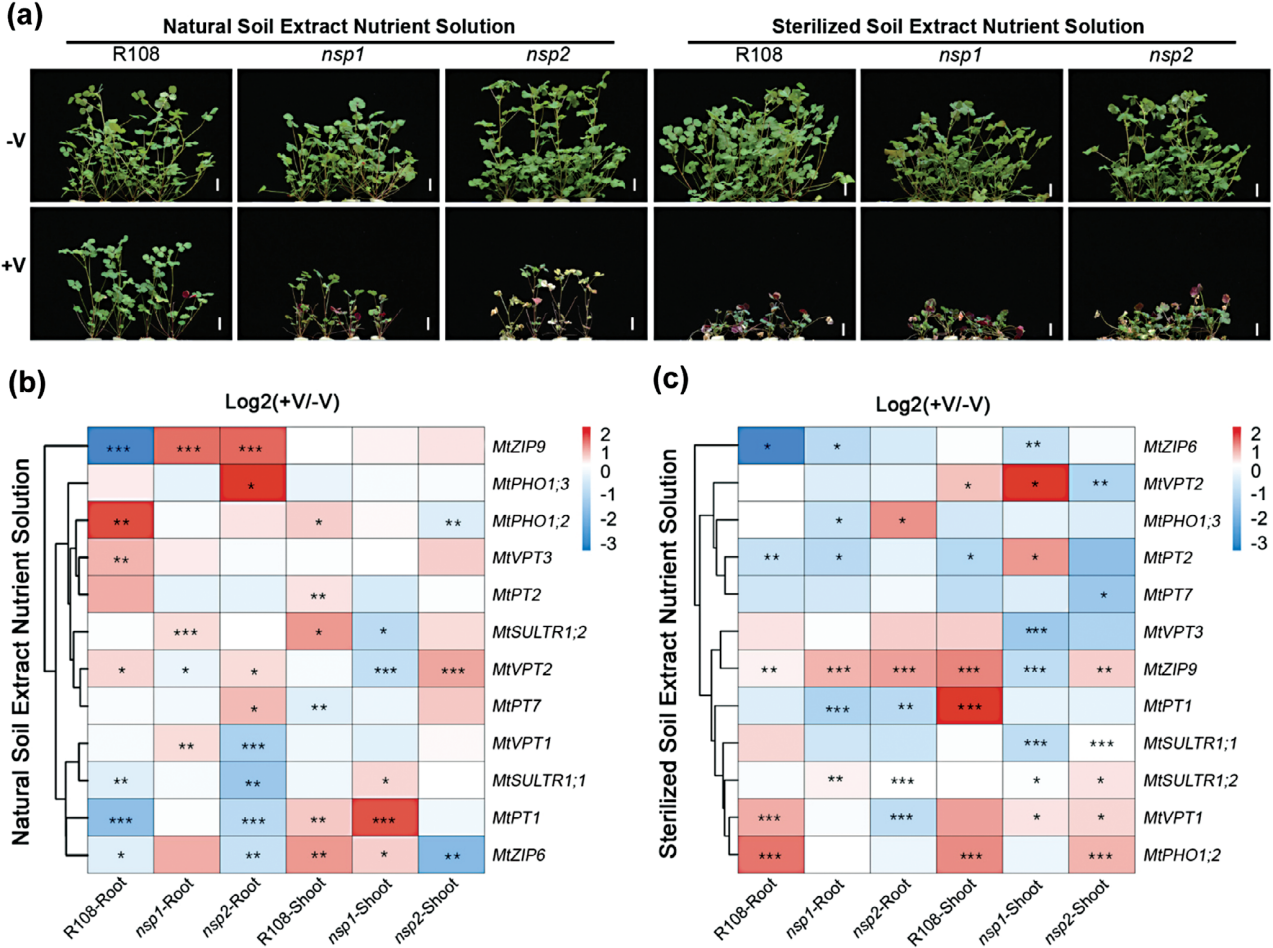
NSP1 and NSP2 are required for both plant tolerance to vanadium (V) and the response of phosphate (P), iron (Fe), and sulfate (S) transporter genes and are also dependent on the existence of soil microbes. a) Growth phenotypes of wild‐type (R108) and *nsp1* and *nsp2* mutant *Medicago truncatula* plants under V stress in the presence and absence of soil microorganisms. Three‐week‐old seedlings were cultured with ½‐strength Hoagland nutrient solution prepared with non‐sterile (Natural) or sterile soil extract (Sterilized Soil Extract Nutrient Solution). After nodulation of R108, plants were treated with (+V) or without (‐V) 30 mg L^−1^ V for 1 week. Bars, 2 cm. b,c) Heatmap of relative expression levels of P, Fe, and S transporter genes in the shoots and roots of wild‐type (R108), *nsp1*, and *nsp2* plants under V stress in the presence (b) and absence (c) of soil microorganisms. Plants were treated as in (a). The color codes indicate the fold changes in gene expression after V treatment, expressed as log_2_[(expression with V (+V))/(expression without V (‐V))]. Data were calculated based on the values in Figures S10–S14 (Supporting Information). Asterisks indicate statistically significant differences based on independent sample *t*‐tests (* *P* < 0.05, ** *P* < 0.01, *** *P* < 0.001).

To investigate the relationship between V tolerance differences and gene expression responses in R108, *nsp1*, and *nsp2*, as well as their dependence on soil microorganisms, we conducted experiments using sterilized soil. Unexpectedly, some R108 plants had higher V tolerance than *nsp1* and *nsp2* plants, while some R108 plants, like all *nsp1* and *nsp2* plants, died under V stress (Figure S20a, Supporting Information). After their roots were separated from the soil, R108 plants with high V tolerance still survived owing to their nodulation induced by symbiosis with *Rhizobium* (Figure S20b, Supporting Information). Thus, it is difficult to completely inactivate microorganisms in the soil. Furthermore, we utilized a hydroponic device with an absorbent slope (HDAS) to grow plants with sterilized or unsterilized soil extract containing ½‐strength Hoagland's nutrient solution,^[^ ^17^ ^]^ so as to better control and observe the *Rhizobium* symbiosis (**Figure** **5**; Figure S21, Supporting Information). The growth status of R108 under these conditions was significantly better than that of *nsp1* and *nsp2* without sterilization (Figure 5a). However, there was no significant difference between them after sterilization, indicating that the difference in V tolerance between R108 and *nsp1* or *nsp2* depends on *Rhizobium* and rhizosphere microorganisms.

Based on the phenotypic results of mutants on V tolerance (Figure 4), we analyzed the gene expression of several members of the *PHT1*, *VPT*, *PHO1*, *ZIP*, and *SULTR1* families in *M. truncatula* (Figures S22–S26, Supporting Information). Without sterilization, the expression levels of *MtPT1*, *MtZIP6*, *MtZIP9*, and *MtSULTR1;1* were significantly reduced in R108 roots, but under sterilized conditions, the expression of these genes remained unchanged or even upregulated (Figure 5b,c). In *nsp1* and *nsp2* roots, this trend was weakened or even the opposite trend occurred, especially for *MtZIP9*. In addition, *MtPHO1;2*, *MtVPT2*, and *MtVPT3* were upregulated in R108 roots, but showed no change in *nsp1* and *nsp2* roots. Among these genes, the responses of *MtVPT2* and *MtVPT3* to V stress weakened after sterilization.

In the shoots, only the expression of *MtPT7* was significantly inhibited by V stress in R108, while its inhibition was not significant in *nsp1* and *nsp2* shoots (Figure 5b,c). *MtPT1*, *MtPT2*, *MtPHO1;2*, *MtZIP6*, and *MtSULTR1;2* were significantly upregulated by V stress in R108 roots, but most of these genes were not significantly affected by V stress, or their expression even decreased, in *nsp1* and *nsp2* roots. Among these genes, *MtPT1*, *MtPHO1;2*, and *MtSULTR1;2* were also significantly induced by V stress in R108 shoots after sterilization, and the change was not significant in *nsp1* shoots. These results indicate that mutations in *NSP1* and *NSP2* can significantly weaken or change the expression response of P, Fe, and S transporter genes to V stress, and their expression response in roots is more dependent on *Rhizobium* or soil microorganisms than that in shoots.

### 2.6 *NSP1* and *NSP2* Mutations Alter Rhizobacterial Diversity under V Stress

We performed 16 rRNA gene sequencing of rhizobacteria. Simpson, hao1, ACE, and Shannon indices were used to assess rhizobacterial α‐diversity (Figure S27, Supporting Information). The treatment with a high concentration of V (2000 mg L^−1^) significantly increased the Simpson index of *nsp1*, indicating a significant decrease in community diversity (Figure S27a, Supporting Information). Second, V treatment significantly reduced the Chao1 and ACE indices of *nsp1*, indicating a significant decrease in community richness (Figure S27b,c, Supporting Information). Under 200 mg L^−1^ V treatment, the Shannon's index of *nsp2* increased significantly, but the Chao1 and ACE indices did not change significantly, suggesting that the uniformity of the community increased significantly (Figure S27b–d, Supporting Information). However, V treatment had no significant effect on any of the diversity indices for wild‐type R108 (Figure S27, Supporting Information). This suggests that the *NSP1* mutation is not conducive to maintaining the α‐diversity of rhizobacteria under V stress (especially in terms of richness), while the *NSP2* mutation has relatively little effect on it.

**Figure 6:**
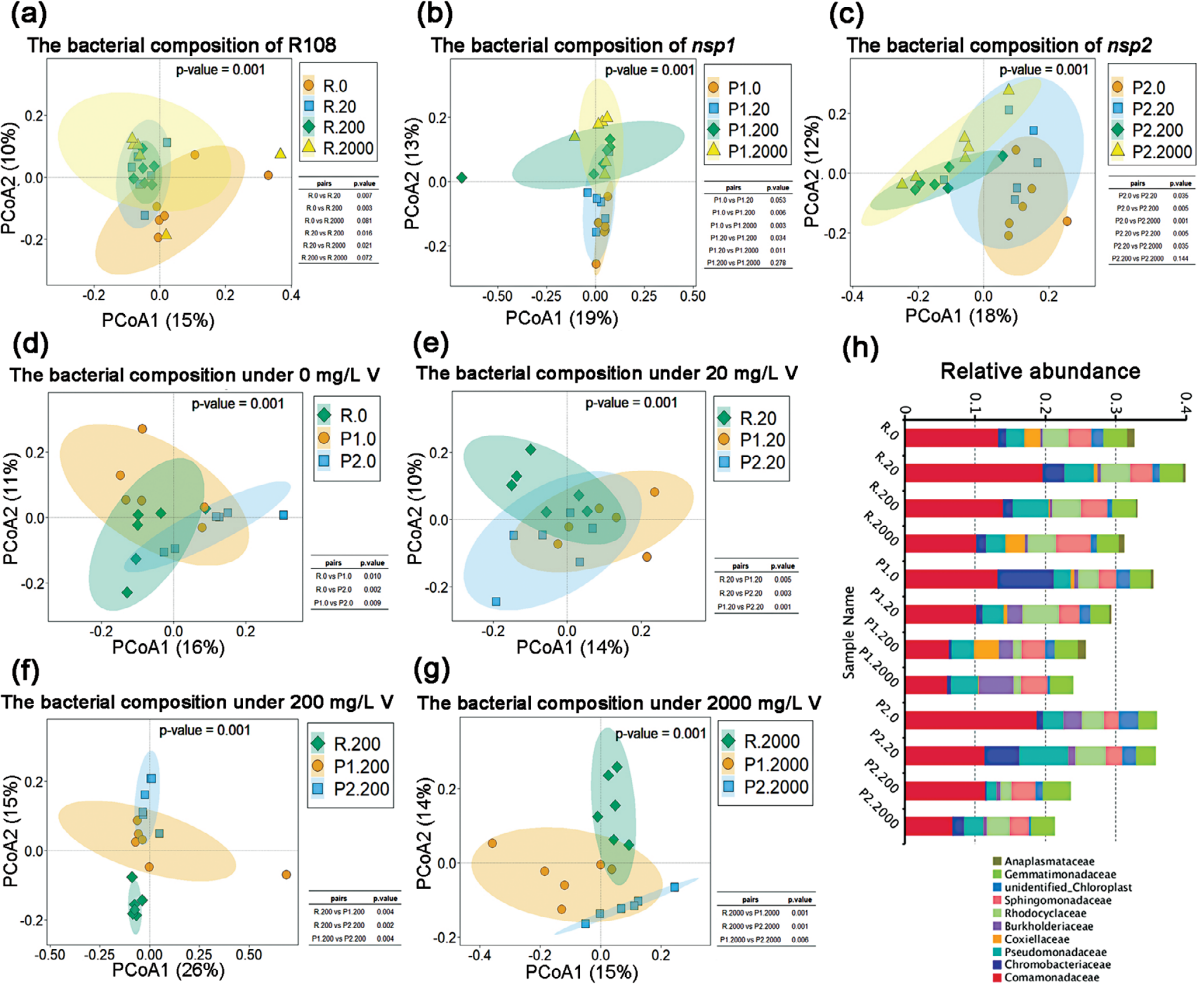
*NSP1* and *NSP2* mutations alter the β‐diversity and relative abundance of rhizobacteria under vanadium (V) stress. a–c) Principal coordinate analysis (PCoA) depicting the β‐diversity of rhizobacteria in wild‐type (R108) (a), *nsp1* (b), and *nsp2* (c) *Medicago truncatula* plants treated with different V concentrations. In the legend, R, P1, and P2 represent R108, *nsp1*, and *nsp2*, respectively, while 0, 20, 200, and 2000 represent V treatment concentrations in mg L^−1^. The table presents the corresponding *P*‐values for comparisons between different concentrations within the same plant material. d–g) Principal coordinate analysis (PCoA) of β‐diversity of rhizobacteria among wild‐type (R108), *nsp1*, and *nsp2* treated with 0 (d), 20 (e), 200 (f), or 2000 (g) mg L^−1^ V. The meaning of the legend and the content shown in the table are consistent with (a–c). Permutational multivariate analysis of variance (PERMANOVA) (a–g) was conducted based on Bray–Curtis distances with a 90% confidence interval. h) Relative abundance of the top ten rhizobacterial families in wild‐type (R108), *nsp1*, and *nsp2* plants. R, P1, and P2 represent R108, *nsp1*, and *nsp2*, respectively. The numbers following the points of R, P1, and P2 indicate the V treatment concentration in mg L^−1^.

Additionally, β‐diversity can reflect the response of the rhizobacterial community structure of R108, *nsp1*, and *nsp2* to V treatment. We used principal coordinate analysis (PCoA) and permutational multivariate analysis of variance (PERMANOVA) to visualize and quantify the differences between microbial communities (β‐diversity) using Bray‐Curtis distance (**Figure** **6**). The β‐diversity of rhizobacterial communities associated with R108, *nsp1*, and *nsp2* were significantly affected by V stress (Figure 6a–c). However, the inter‐group distribution associated with R108 was more concentrated than that of *nsp1* and *nsp2*, indicating that the rhizobacterial community structure of *nsp1* and *nsp2* was more affected by V treatment than was that of R108. Then, we analyzed the β‐diversity of rhizobacterial communities among R108, *nsp1*, and *nsp2* plants at the same V concentration. The results showed that there were significant differences among R108, *nsp1*, and *nsp2* across all conditions (Figure 6d–g). However, without the addition of V, the rhizobacteria associated with R108 overlapped with those of *nsp1* and *nsp2*, but they gradually separated along principal coordinate axis 2 as V treatment concentration increased. This indicated that V treatment increased the difference in rhizobacterial community structure between R108 and both *nsp1* and *nsp2*.

### 2.7 *NSP1* and *NSP2* Mutations Alter the Abundance and Co‐Occurrence Networks of Rhizobacteria under V Stress

To investigate the effects of V treatment on the dominant rhizosphere bacteria associated with R108, *nsp1*, and *nsp2*, we determined the most abundant families. Comamonadaceae was the most abundant family in all samples and decreased as V treatment concentration increased (Figure 6h; Figure S28a, Supporting Information). However, its abundance was significantly higher in association with R108 than with *nsp1* and *nsp2* under various V treatments. Second, the abundance of Burkholderiaceae in *nsp1* rhizosphere soil was significantly higher than that in R108 rhizosphere soil (Figure S28b, Supporting Information). Then, the abundance of Pseudomonadaceae was significantly increased by 20 and 200 mg L^−1^ V treatments for R108 but did not significantly change for *nsp1* and *nsp2* (Figure S28c, Supporting Information). Lastly, the abundance of Rhodocyclaceae remained at a relatively high and stable level for R108 but was easily disturbed by V stress for *nsp1* and *nsp2* (Figure S28d, Supporting Information).

**Figure 7:**
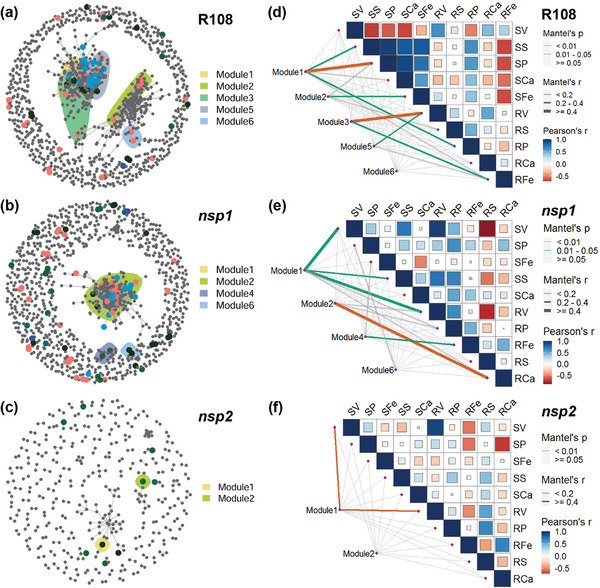
*NSP1* and *NSP2* mutations alter rhizobacterial co‐occurrence networks. a–c) Co‐occurrence networks of rhizobacteria in wild‐type (R108) (a), *nsp1* (b), and *nsp2* (c) *Medicago truncatula* plants. Low frequency operational taxonomic units (OTUs) with frequencies less than 92% were removed from all samples. V‐responsive OTUs (VrOTUs) are highlighted and color‐coded. The edges of the co‐occurrence network were selected based on criteria of Spearman's rho > 0.7 and *P*‐value < 0.001. d–f) Mantel test of plant element concentrations and VrOTU modules in R108 (d), *nsp1* (e), and *nsp2* (f). Plant element concentration data were sourced from Figure 2. Lines with significant correlations are indicated in color.

We used co‐occurrence networks to explore differences in the response patterns of R108, *nsp1*, and *nsp2* rhizobacteria to V treatment (**Figure** **7**). By using edgeR and indicator species methods, V‐responsive operational taxonomic units (VrOTUs) were identified and mapped onto co‐occurrence networks, and their distribution characteristics were then analyzed. The rhizobacterial co‐occurrence networks driven by V stress were significantly simpler for *nsp2* than those associated with R108 and *nsp1* (Figure 7a–c). In R108, two major co‐occurrence network modules were formed, whereas there was only one in *nsp1* and almost none in *nsp2*. In addition, under V treatment, R108 formed more modules containing VrOTUs than did *nsp1* and *nsp2*, and most of the VrOTUs mostly came from two V treatments, 0 and 200 mg L^−1^ (Figure S29, Supporting Information). Thus, compared to R108, under V treatment there was less symbiosis with rhizobacteria for *nsp1* and especially *nsp2*.

We also constructed co‐occurrence networks of rhizobacteria from R108, *nps1*, and *nsp2* plants at the same V concentration and used edgeR and indicator species methods to identify the species‐differentiated operational taxonomic units (SdOTUs). Overall, the number of modules containing SdOTUs decreased under V treatment compared to normal conditions (Figure S30, Supporting Information). However, there was an increase in the complexity of the bacterial network in the rhizosphere under the 200 mg L^−1^ V treatment in comparison with the 20 mg L^−1^ treatment (Figure S30b,c, Supporting Information). Under normal conditions, the SdOTUs in the network were mostly associated with R108, but under V treatment, they were mostly associated with *nsp2* (Figure S30, Supporting Information). Also, modules in all networks that primarily contain R108 SdOTUs were separated from modules that primarily contain *nsp1* and *nsp2* SdOTUs. These results suggest that *NSP1* and *NSP2* mutations reshape rhizobacterial communities and alter the ability of rhizobacterial interactions to cope with V stress.

### 2.8 *NSP1* and *NSP2* Mutations Alter the Association Between Rhizobacteria and Accumulation of Elements in Plants

There may be an intrinsic relationship between rhizosphere microorganisms and the accumulation of heavy metals and nutrients in plants. To investigate this, we performed a correlation analysis between the modules and the element concentrations in the rhizobacterial co‐occurrence networks. There was a more significant correlation between the VrOTU modules and element concentrations in R108 rhizosphere soil compared to *nsp1* and *nsp2* rhizosphere soil (Figure 7d–f). In R108, four out of five VrOTU modules were significantly correlated with elements in plants. Among these, module 3 and module 5 were significantly correlated with V concentrations in roots, and module 1, module 2, and module 3 were significantly correlated with P, Fe, and S concentrations in shoots or roots. However, no modules were associated with shoot V concentrations and plant Ca concentrations (Figure 7d). In *nsp1*, three out of four VrOTU modules were significantly associated with plant elemental concentrations. Among them, module 1 was significantly correlated with V concentrations in both shoots and roots and with S concentrations in shoots. Module 2 and module 4 were significantly correlated with Fe and Ca concentrations, respectively, in roots. However, no modules were significantly associated with P (Figure 7e). In *nsp2*, of the two modules, only module 1 was significantly correlated with the concentration of any element, and it was significantly correlated with V concentrations in shoots and roots, but not with concentrations of other elements (Figure 7f).

**Figure 8:**
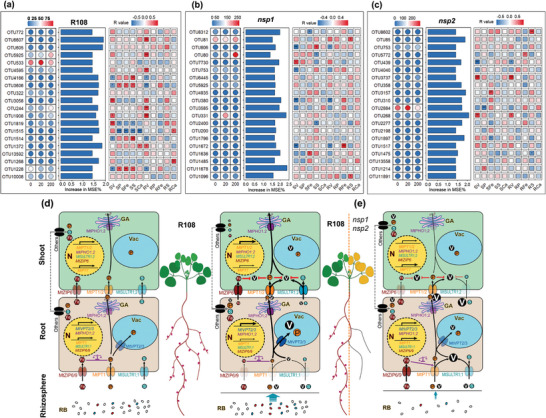
*NSP1* and *NSP2* mutations alter the rhizobacterial biomarkers and association between biomarkers and plant element concentrations. a–c) Abundance and importance assessment of rhizobacterial biomarkers in R108 (a), *nsp1* (b), and *nsp2* (c) *Medicago truncatula* plants, along with Spearman's correlation with plant element concentrations. The bubble chart displays the frequency of the top 20 biomarkers. The bar chart shows the importance of the top 20 biomarkers. The correlation matrix illustrates the relationships between the top 20 biomarkers and plant element concentrations. Plant element concentration data were sourced from Figure 2. Spearman's correlation coefficients were calculated with statistical significance indicated as follows: * *P* < 0.05, ** *P* < 0.01, and *** *P* < 0.001. d,e) A proposed model illustrating the response mechanism of *Medicago truncatula* to vanadium (V) stress and the effect of *NSP1* and *NSP2* mutations on it. In (d), V may share transporters with phosphate (P) and sulfate (S). Under V stress, the wild‐type (R108) plant downregulates the expression of P and S transporter genes (*MtPT1* and *MtSULTR1;1*) in roots to reduce V absorption. Furthermore, the upregulation of *MtVPT2*, *MtVPT3*, and *MtPHO1;2* accelerate the vacuolar compartmentalization of V and its loading into other tissues to reduce the toxicity of V to roots. The downregulation of the Fe transporter genes *MtZIP6* and *MtZIP9* ensures the response of P transporter genes to V through Fe‐P balance regulation. The upregulated expression of *MtZIP6*, *MtPT1*, *MtPT2*, *MtSULTR1;1*, and *MtSULTR1;2* in the shoots may increase the ability of Fe, P, and S to resist V toxicity. However, the tolerance of R108 to V and the expression response of genes in roots largely depend on the presence of soil microbes. R108 can maintain the diversity of the rhizobacterial community under V stress and actively regulate rhizobacteria to coordinate the response to V stress. In (e), both *nsp1* and *nsp2* mutants cannot form nodules. Subsequently, P, S, and Fe transporter genes are unable to respond positively or appropriately to V stress. Additionally, the rhizobacteria become fragile and disordered. Therefore, *nsp1* and *nsp2* exhibit a more sensitive V stress phenotype compared to R108.

Bacteria outside the modules may also be important. Therefore, we further mined the biomarkers in rhizobacteria and explored the correlation between them and the element concentration in plants (**Figure** **8**). We identified the 20 most important biomarkers in R108, *nsp1*, and *nsp2* and analyzed their association with elemental concentrations in plants. The biomarker with the highest score for R108 was classified in the Verrucomicrobiota, while the biomarker with the highest score for *nsp1* and *nsp2* was classified in the Proteobacteriota (Figure 8; and Table S1, Supporting Information). Proteobacteriota, Acidobacteriota, Gemmatimonadota, and Bacteroidota were the common bacterial biomarkers of the rhizospheres of the R108, *nsp1*, and *nsp2* plants (Figure 8; Figure S31, Supporting Information). Moreover, Proteobacteria had a high relative abundance among the rhizobacterial biomarkers of the three plant materials; however, compared to *nsp1* and *nsp2*, its relative abundance was more stable in R108 (Figure S31, Supporting Information). In addition, Verrucomicrobiota, Nitrospirota, and Bdellovibrionota were unique to being R108 biomarkers. These results indicate that the *NSP1* and *NSP2* mutations alter the biomarkers of the rhizobacteria associated with *M. truncatula* treated with V.

Correlation analysis of the selected biomarkers with plant element concentrations showed that the number of significant correlations was significantly higher in R108 than in *nsp1* and *nsp2* (Figure 8). In R108, more than half of the biomarkers were significantly correlated with V concentrations in roots, and most of them were significantly positively correlated (Figure 8a). Among them, OTU3058 and OTU1908 were positively correlated with V concentrations in shoots and roots. In addition, a large number of biomarkers were significantly correlated with P, Fe, and S concentrations in shoots. Among them, OTU1228 and OTU3606, belonging to Acidobacteriota and Bdellovibrionota, respectively, were significantly negatively correlated with V concentrations in shoots and roots, but also significantly positively correlated with P, Fe, and S concentrations in shoots. OTU1514 and OTU4166, belonging to Chloroflexota and Gemmatimonadota, respectively, were negatively correlated with V concentrations in shoots and roots and positively correlated with S concentrations. In addition, OTU1819, which belongs to the Bacteroidota, had a significant positive correlation with the concentration of V in shoots and a significant negative correlation with the concentrations of P and S in shoots. Compared to R108, the number of biomarkers significantly correlated with V concentrations in *nsp1* was significantly reduced, and the correlations were mainly negative (Figure 8b). Meanwhile, the number of biomarkers significantly correlated with other nutrient elements was significantly lower, and there was no significant correlation with P. In *nsp2*, fewer biomarkers were significantly correlated with V concentrations, and no significant correlations were found between them and other nutrient elements (Figure 8c). These results suggested that mutations in *NSP1* and *NSP2* weaken or alter the association of rhizobacteria with elements in plants, which may have caused their decreased V tolerance.

## 3 Discussion

Previous studies have shown that *Rhizobium* symbiosis plays an important regulatory role in the uptake and accumulation of heavy metals in legumes.^[^ ^21^ ^]^ NSP1 and NSP2 are key transcriptional regulators of *Rhizobium* symbiosis signaling.^[^ ^22^ ^]^ In this study, we found that the V tolerance levels of *nsp1* and *nsp2* mutants with *Rhizobium* symbiosis defects were significantly lower than that of wild‐type R108. Furthermore, it was confirmed from physiological and genetic perspectives that the absorption and accumulation of V are closely related to the absorption, accumulation, and homeostasis of P, Fe, and S. Moreover, rhizosphere microorganisms also play an important role in V tolerance in plants. Finally, from the perspective of P, Fe, and S regulation and rhizobacterial response, we revealed the mechanism of V absorption and accumulation is regulated by NSP1 and NSP2.

### 3.1 NSP1 and NSP2 Modulate V Accumulation and Tolerance in Plants through P, Fe, S, and their Transporters

Because of their similar structure and charge, V is considered to be a P analog and is absorbed by plants through the P transport pathway. When P conditions were changed under a certain V stress, the change of total P level in plants was very consistent with that of V, which further confirmed the possibility of V and P sharing transport system (Figure S4b,c, Supporting Information). However, there has been a lack of genetic evidence supporting this conclusion.^[^ ^23^ ^]^ *Medicago truncatula VPTs* mutant *mtvpt3* and *A. thaliana* P transporter mutants *pht1;1*, *pht1;9*, *vpt1*, and *pho1* exhibit varying degrees of stronger V tolerance phenotypes relative Col‐0 in shoots. To our knowledge, this is the first genetic support for previous inferences on the shared transport pathway of V and P (Figure 4a–c). However, the roots of *vpt1* exhibited a sensitive phenotype (Figure S16, Supporting Information). Recently, our research has shown that mutations in *MtVPT2* can reduce the efficiency of P transport from roots to shoots.^[^ ^24^ ^]^ *Arabidopsis thaliana VPT1* is closely related to *MtVPT2*, and mutations to it may reduce the efficiency of V transport from roots to shoots. Therefore, *vpt1* showed a phenotype of tolerance in shoots and sensitivity in roots under V stress. In addition, VPT1 is one of the most important transporters in *A. thaliana* that transports P from cytoplasm to vacuoles.^[^ ^10^ ^]^ Mutations to *VPT1* may lead to V not being effectively transported to root vacuoles for detoxification. *MtVPT2* and *MtVPT3* in R108 roots were significantly induced by V stress, which may be conducive to detoxification by vacuolar compartmentalization. Therefore, overexpression of *VPT1* and *MtVPT3* can significantly improve the V tolerance of plants. Also, *MtVPT3* overexpressed plants had higher P and V concentrations in shoots than the wild type (Figure S14j,k, Supporting Information).

However, *MtVPT3* mutations can also endow plants with V tolerance, but the mechanism of the effect may be different from that of *vpt1*. The mutation of *MtVPT3* can increase the efficiency of P transport from roots to shoots,^[^ ^24^ ^]^ but this also means that it inevitably will increase the absorption and transportation of V. As expected, V concentrations in *mtvpt3* shoots were higher than in wild‐type R108 under V stress (Figure S14c, Supporting Information). Unexpectedly, the concentrations of total P and Pi in *mtvpt3* shoots were lower than those in R108 (Figure S14d, Supporting Information), which is different from the previously reported results under normal conditions.^[^ ^24^ ^]^ However, the decrease in Pi concentration means that less Pi is likely to be stored in the vacuole, which favors more P in the cytoplasm to counter V toxicity. Higher P levels in the environment are more conducive to the survival of plants under V stress, which to some extent reflects the role of P in resistance to V toxicity (Figures S4 and S17, Supporting Information). In the root of *mtvpt3*, besides the significantly lower V concentration than the wild type, the higher P/V ratio may be also conducive to the V tolerance (Figure S14f,h, Supporting Information). In addition, the levels of S and Ca in the shoots and S, Fe and Ca in the roots of *mtvpt3* were significantly higher than those of the wild type (Figure S14d,g, Supporting Information). Similar to high P, high S and high Fe can also antagonize the toxicity of V (Figure 3; Figures S7,S8,S10, and S11, Supporting Information). Interestingly, *MtVPT3* overexpressing plants and *mtvpt3* mutants had very similar changes in the concentrations of S, Fe and Ca in shoots (Figure S14d,k, Supporting Information). This suggests that although they increase V levels in the shoots through different mechanisms, they have similar mechanisms for combating V toxicity. However, how *MtVPT3* regulates S, Fe and Ca levels needs to be further explored in the future. PHT1 and PHO1 determine the process of P absorption, loading, or redistribution in plants.^[^ ^25^ ^]^ The V‐tolerance phenotypes in shoots of their mutants may be owing to the obstruction of V absorption and transportation of P. Correspondingly, *MtPT1* in the roots of R108 was significantly downregulated by V stress, which may be beneficial for reducing V absorption and transport. In addition, *MtPHO1;2* was significantly induced by V stress in R108 roots, which may be the result of a decrease in P. Of course, the upregulation of its expression may also be an active process by which plants resist V stress, for example, through reducing the level of V in the root cytoplasm. However, these genes in *nsp1* and *nsp2* mutants exhibited little response to V stress or a response that was opposite to that of R108, such that they were sensitive to V stress (Figure 5b,c).

So far, no research has shown that the absorption of V in plants is related to S transport. However, S and P have certain similarities in their chemical structure, so some members of the SULTR family of S transporters also have an affinity for P.^[^ ^26^ ^]^ As a P analogue, V may also share transport pathways with S. We found that *A. thaliana SULTR* family mutants have different V tolerance phenotypes, especially mutants of genes in the *SULTR1* subfamily. In addition, increasing the concentration of SO_4_^2−^ in the growth medium may competitively inhibit the absorption and transport of V, thus alleviating the toxicity of V to plants (Figure S19c, Supporting Information). SULTR1;2 is not only a high‐affinity S transporter that is more important than SULTR1;1, but it is also likely a S receptor that regulates the response of other genes to S.^[^ ^27^ ^]^ Under low S conditions, mutations in *SULTR1;2* may be able to limit V uptake and thus confer a V‐tolerant phenotype to plants (Figure S19c,d, Supporting Information). However, under high S conditions, mutations in *SULTR1;2* may also reduce V sensitivity while reducing S sensitivity, thereby reducing the ability to cope with V stress, and thus causing roots to exhibit a sensitive phenotype. The expression of *MtSULTR1;1* in roots was significantly inhibited by V stress, which may be conducive to reducing the absorption of V by roots of *M. truncatula*. However, *MtSULTR1;1* showed no response to V stress in *nsp1* roots. P and S are necessary for *Rhizobium* symbiosis.^[^ ^28^ ^]^ R108, which successfully formed symbioses with *Rhizobium*, may have a stronger P and S uptake capacity than both *nsp1* and *nsp2*. Howeover, V is also likely to be more absorbed during the uptake of P and S. It is well known that V can inhibit ATPase activity, which in turn affects transporter activity. In this case, R108 still actively inhibits the absorption and transport pathways of nutrient elements through gene expression regulation. Therefore, V transported to shoots under the low concentration of V (20 mg L^−1^) was more in R108 than both *nsp1* and *nsp2*, while various nutrient element levels dropped sharply in R108 (Figure 2a). However, R108 is more effective than *nsp1* and *nsp2* in limiting V absorption, transport, and detoxification through the expression and regulation of P and S transporters. Therefore, under high‐concentration V stress (200 mg L^−1^), R108 accumulated lower V levels than both *nsp1* and *nsp2* and showed stronger V tolerance (Figures 1 and 2a).

In addition to these results based on P and S transporter mutants, we also found that the shoot V tolerance of the *A. thaliana* mutant *irt1* was higher than that of wild‐type plants. Owing to the formation of insoluble complexes, there is an antagonistic interaction between Fe and P in plants.^[^ ^29^ ^]^ For the *IRT1* mutation, the P in the shoots of a plant increases under normal conditions (Figure S15, Supporting Information). However, Fe deficiency in plants through the degradation of the P signaling central factor PHRs occurs to avoid P overload. When V enters plants, it may be incorrectly identified by plant systems as P and affect the accumulation of Fe. Therefore, Fe, P and V may form a mutually balanced and antagonistic relationship in plants. The translocation factor of V from root to shoot was significantly increased under low Fe condition (Figure S7c, Supporting Information). In order to antagonize V toxicity, P levels in the shoots were also increased (Figure S7d, Supporting Information). Surprisingly, the increase of V also stimulated a significant increase in Fe levels in the shoots under low Fe conditions (Figure S7e, Supporting Information). Under the condition of Fe deficiency, the concentration of Fe in the root decreased significantly, and correspondingly the concentration of P and V also decreased significantly (Figure S7b,d,e, Supporting Information). For Fe‐deficient *irt1*, V may inhibit P absorption and loading more strongly than for Col‐0. Of course, this effect will also inhibit the transport of V; thus, *irt1* shows a V‐tolerant phenotype, and the P level is lower than that of the wild type under V stress. Previous studies have shown that MtZIP6, which is closely related to IRT1 in *M. truncatula*, has Fe transport activity.^[^ ^30^ ^]^ Although the expression of *MtZIP6* was inhibited by V stress in wild‐type R108 roots, the response of its homologous gene *MtZIP9* to V stress was more prominent (Figure S25a,b, Supporting Information). The significant downregulation of *MtZIP6* and *MtZIP9* in R108 roots may be conducive to inhibiting the transport of V through P regulation, but their responses in *nsp1* or *nsp2* were not obvious or significantly upregulated. Therefore, *nsp1* and *nsp2* have higher Fe concentrations than R108, but exhibit more sensitive phenotypes to V than did R108 (Figure 5a). In addition, there is often retention between V and Fe in the environment, which may also exist in plants.^[^ ^31^ ^]^ When the Fe concentrations in the environment increase, the roots of wild‐type plants can effectively absorb Fe to retain V and exhibit a reduced toxicity phenotype, but the performance of *irt1* was not obvious (Figure S18, Supporting Information).

The response of the above transporter genes limits the absorption and transport of V by roots, but it also led to a significant decrease in the levels of P, Fe, and S in the shoots of R108 (Figure 2c–e). However, this was not evident in *nsp1* and *nsp2* mutants. This finding also implies that R108 preferentially adopts a strategy of limiting toxic ion transport at the expense of nutrient accumulation, rather than resisting ion toxicity, to adapt to V stress. With the increase of V concentration in the environment, it is inevitable that the concentration of V in plant shoots will increase. Therefore, the V concentrations in the shoots of R108 were significantly negatively correlated with the P, Fe, and S concentrations (Figure 2g–i). However, this pattern was significantly reduced or even opposite in *nsp1* and *nsp2* mutants. In contrast with the pattern observed in the roots, the P, S, and Fe transporter genes in the shoots of R108 were significantly upregulated under V stress, but most of them had no significant effect or their expression was decreased in *nsp1* and especially *nsp2* (Figure 5b,c). The upregulation of these transporter genes may be induced by the decrease of P, Fe, and S levels, or it may be a process of actively responding to V stress. Compared with the low concentration of V (20 mg L^−1^), the high concentration of V (200 mg L^−1^) stress significantly increased the P, Fe, and S levels in the shoots of R108 (Figure 2c–e). The increase of P, Fe, and S levels is conducive to combating the toxicity of V, which may be a strategy that is initiated gradually as V concentration increases. Although the levels of P and Fe in the shoots of *nsp1* and *nsp2* mutants were still higher than those in R108 under 200 mg L^−1^ V stress, they were unable to cope with the toxicity caused by the significant increase in V (Figure 2a,c,d). If P, S, and Fe are antagonistic to V, then P/V, S/V, and Fe/V ratios may be more important for V tolerance than simple changes in element content. Compared with 20 mg L^−1^ V stress, 200 mg L^−1^ V stress significantly increased the P/V, S/V and Fe/V ratios in R108 shoots (Figure S3, Supporting Information). However, this trend was reversed in *nsp1* and *nsp2*. Increasing P, S, and Fe levels in the environment significantly enhanced V tolerance in plants (Figures S4,S7, and S10, Supporting Information). However, the V tolerance changes of *nsp1* and *nsp2* differed from that of R108 in response to P, S, and Fe conditions. The P/V ratio was significantly increased in R108, but not in *nsp1* and *nsp2* under high P conditions (Figure S4d, Supporting Information). Therefore, V‐sensitive phenotypes of *nsp1* and *nsp2* are more likely to appear under high P conditions. The S/V ratio in *nsp1* and *nsp2* was significantly lower than that in R108 under low S conditions (Figure S10d, Supporting Information). However, high S conditions significantly increased the S/V ratio in plants, especially in *nsp1* and *nsp2*. Therefore, V‐sensitive phenotypes of *nsp1* and *nsp2* are more likely to appear under low S conditions. Under low Fe conditions, the Fe/V ratio was significantly increased in R108, but not in *nsp1* and *nsp2* (Figure S7f, Supporting Information). In addition, the P/V ratio did not change significantly in R108, but decreased significantly in *nsp1* and *nsp2*. Therefore, V‐sensitive phenotypes of *nsp1* and *nsp2* are more likely to appear under low Fe conditions. Besides the effects of P, Fe, and S, there may be other mechanisms that endow R108 with V tolerance. In addition, whether under low or high V conditions, R108 has a significantly higher V transfer coefficient from root to shoot than did *nsp2* (Figure 2b). This is difficult to explain through patterns of gene expression in roots or shoots, and further exploration is merited. Compared with the expression response of P, Fe, and S transporter genes in the shoots, the responses of these genes in R108 roots were more dependent on *Rhizobium* and soil microorganisms. Therefore, soil microorganisms may also play an important role in regulating plant V tolerance.

### 3.2 NSP1 and NSP2 are Essential for Maintaining Rhizobacterial Diversity and Cooperative Response Capacity under V Stress

Previous studies have shown that the diversity of rhizosphere microbial communities responds strongly to V stress, and that the colonization of some key microorganisms plays a key role in improving V tolerance in plants.^[^ ^32^ ^]^ *Rhizobium*–legume symbiosis can lead to changes in rhizosphere microbial communities. However, it is unclear whether this affects V tolerance. Our study shows that the V tolerance of *M. truncatula* depends on the presence of rhizosphere microorganisms. The mutation of *NSP1* rendered *Rhizobium* unable to form symbioses with plant roots, and it was also not conducive to maintaining α‐diversity of rhizobacteria under V stress (Figures S1 and S27, Supporting Information). Furthermore, β‐diversity analysis showed that the rhizobacterial community structures of *nsp1* and *nsp2* mutants were more affected by V treatment than that of R108 (Figure 6a–c). Therefore, *Rhizobium* symbiosis may affect the sensitivity of the legume rhizosphere microbial community to V stress. The enrichment of beneficial micro‐organisms is a possible way for plants to resist V stress. They can promote plant growth under heavy metal stress by producing plant growth promoting substances, plant hormones, enzymes, etc., or they can chelate heavy metals or change the valence of heavy metals to reduce heavy metal damage to plants.^[^ ^33^ ^]^ Previous studies have shown that Comamonadaceae, Pseudomonadaceae, and Rhodocyclaceae may comprise bacteria with V reduction ability in V‐contaminated soil, and their abundance increases at low V concentrations. However, their growth can also be inhibited by high levels of V stress.^[^ ^34^ ^]^ Our study showed that the abundance of Comamonadaceae in the rhizosphere of R108 under V stress was significantly higher than that of *nsp1* and *nsp2* (Figure S28a, Supporting Information). Pseudomonadaceae had a positive response to V stress for R108, but had no significant change for *nsp1* and *nsp2* mutants (Figure S28c, Supporting Information). The abundance of Rhodocyclaceae decreased rapidly for *nsp1* and *nsp2* mutants under high V stress, but was relatively stable for R108 (Figure S28d, Supporting Information). Therefore, the response of these taxa may be the reason why *nsp1* and *nsp2* mutants were more sensitive to V stress than wild‐type R108. Other studies have shown that some bacteria in Burkholderiaceae can dissolve insoluble inorganic P in soils, thereby promoting P uptake by plants.^[^ ^35^ ^]^ Perhaps, because of the similarity of V to P, these bacteria also promote the uptake of V by plants. In our study, the abundance of Burkholderiaceae in *nsp1* was higher than that in R108, which may be one reason why the V concentration of *nsp1* was higher than that of R108 when treated with a high concentration of V (≥200 mg L^−1^) (Figure S28b, Supporting Information).

Changes in rhizobacterial diversity affect community interactions.^[^ ^36^ ^]^ Co‐occurrence networks may provide a new perspective on microbial interaction analysis owing to their strong emergent properties.^[^ ^37^ ^]^ We found that mutations of *NSP1* and *NSP2* weakened the network complexity of rhizobacteria under V treatment and altered the interactions among bacteria (Figure 7a–c). Previous studies have shown that diverse and complex microbial communities tend to be more stable and resilient under external environmental stress relative to simple communities.^[^ ^38^ ^]^ The complex bacterial network in the rhizosphere of R108 may endow it with greater stability and resistance to V stress, thus increasing the V concentration threshold of R108 (Figure 7a–c). Mutations in *NSP1* and *NSP2* weakened the cooperative relationships among rhizobacteria, and rhizobacteria were more likely to exhibit disrupted homeostasis when exposed to V stress. Therefore, *nsp1* and *nsp2* were more severely stressed by V than was R108. Furthermore, we mapped VrOTUs that responded to V in the co‐occurrence networks, and they formed more subnetwork modules in R108 than both *nsp1* and *nsp2* (Figure 7a–c). This finding implies that R108 may have a stronger ability to drive rhizobacteria to combat V stress than both *nsp1* and *nsp2*. *NSP1* and *NSP2* mutations reshaped rhizobacterial communities (Figure S30, Supporting Information), which may affect plant–microbe and microbe–microbe synergistic responses to V stress.

### 3.3 NSP1 and NSP2 are Required for the Establishment of Associations between Rhizobacteria and Element Accumulations under V Stress

Rhizobacteria have been shown to increase tolerance to heavy metals by promoting nutrient uptake in plants.^[^ ^39^ ^]^ However, the assembly and function of rhizobacteria are strictly regulated by the host.^[^ ^40^ ^]^ Association analysis of rhizobacterial network modules with plant element concentrations showed that more modules in R108 were significantly correlated with V, P, Fe, and S concentrations compared to *nsp1* and *nsp2*. In *nsp1* and *nsp2*, only one module showed a significant association with plant V concentrations, and the associations with P, Fe, and S concentrations were significantly weaker compared to R108 (Figure 7d–f). These findings indicate that rhizobacteria, in addition to directly participating in V stress responses, seem to actively mobilize other elements involved in plant defenses against V stress, but this function is defective in *nsp1* and *nsp2*. Therefore, we further investigated the relationship between rhizobacteria and V tolerance in different plant materials through the development of biomarkers. The most important biomarker of R108 was classified in Verrucomicrobiota, while those of *nsp1* and *nsp2* were classified in Proteobacteria (Figure 8; and Table S1, Supporting Information). Previous studies have shown that Proteobacteria and Verrucomicrobiota have a high tolerance to V and are the main bacteria found in V‐contaminated environments.^[^ ^41^ ^]^ However, in *nsp1* and *nsp2*, the fluctuations in the relative abundance of Proteobacteria were larger than those in R108, but such fluctuations almost completely disappeared for Verrucomicrobiota (Figure S31, Supporting Information). In addition to Bdellovibrionota, R108, unlike *nsp1* and *nsp2*, was also associated with the biomarker Nitrospirota, which is involved in the biological fixation of S and N elements.^[^ ^42^ ^]^ Therefore, in addition to the rhizobacteria associated with V stress, there were also bacteria associated with nutrient cycling that serve as a biomarker of R108, and these bacteria were also abundant.

The above findings have shown that V accumulation and tolerance in plants are related to P, Fe, and S. The association analysis showed that multiple biomarkers in R108 were significantly associated with not only shoot V concentration but also P, Fe, and S concentrations, and this phenomenon was significantly weakened in symbiosis‐defective *nsp1* and *nsp2* mutants (Figure 8). These biomarkers were found to be members of the Acidobacteriota, Bdellovibrionota, Chloroflexota, Bacteroidota, and Gemmatimonadota (Table S1, Supporting Information). Previous studies have shown that members of the Gemmatimonadota are involved in the geochemical cycling of elements such as P and S.^[^ ^43^ ^]^ More importantly, some of the bacteria in Acidobacteriota, Chloroflexota, and Bacteroidota have the ability to reduce pentavalent V to tetravalent V, thus reducing the toxicity of V to plants.^[^ ^44^ ^]^ In our study, Gemmatimonadota, Chloroflexota, Acidobacteriota, and Bacteroidota had a significant negative association with the concentrations of V in shoots and roots of R108 and a significant positive association with the concentrations of P, Fe, and S (Figure 8a). The presence of these bacteria may promote the accumulation of P, Fe, and S while reducing the toxicity of V to R108. In addition, there were several biomarkers in R108 that were significantly positively correlated with V concentrations in shoots and roots (Figure 8a). Most of them belong to Proteobacteria, which also seem to have the ability to reduce the toxicity of V to plants.^[^ ^45^ ^]^ Therefore, R108 reduces the toxic effect of V on itself by recruiting a large number of beneficial rhizobacteria that can reduce V toxicity and promote nutrient circulation, while mutations in *NSP1* and *NSP2* cause defects in this ability of plants to recruit rhizobacteria.

## 4 Conclusion

A proposed model is given in Figure 8d,e. V may share the transport pathways with P and S. Fe may interfere with the absorption and accumulation of V through the regulation of P transport. Mutations or overexpression of P, S, and Fe transporter genes can enhance the V tolerance of plants to varying degrees. Wild‐type R108 *M. truncatula* was able to actively regulate the expression of P, S, and Fe transporter genes to limit the absorption and accumulation of V and resist the toxicity of V. However, the tolerance of R108 to V and the expression response of genes in roots largely depended on the presence of *Rhizobium* symbiosis and/or soil microorganisms. R108 plants were able to maintain the diversity of the rhizobacterial community under V stress and actively regulate the cooperative response of rhizobacteria to V stress. These bacteria were significantly associated with the levels of V, P, S, and Fe in plants and jointly assisted or regulated the ability of plants to cope with V stress. When *NSP1* and *NSP2* were mutated, the symbiosis between roots and rhizobia was defective. Subsequently, the P, S, and Fe transporter genes were unable to respond positively or correctly to V stress, and the rhizobacterial community became unstable and its ability to cope with V weakened. Therefore, the V tolerance of *nsp1* and *nsp2* mutants was significantly lower than that of R108. In terms of V accumulation and tolerance regulation, how NSP1 and NSP2 regulate the expression of P, S, and Fe transporters through nodule signaling and rhizosphere microbiome is a complex and challenging scientific question that deserves further exploration in the future. This study suggests that NSP‐associated root nodule symbiosis or NSPs themselves play an important role in regulating V accumulation and tolerance in legumes, which may provide new ideas for green sustainable management of V pollution and protection of human health.

## 5 Experimental Section

### Materials and Methods

In this study, *M*. *truncatula* ecotype R108 was used as the control material for the *Tnt1*‐insertion mutants *nsp1* (NF9220), *nsp2* (NF10950), and *mtvpt3‐1* (NF3062), which were obtained from the Noble Foundation *Tnt1* database (http://medicago‐mutant.noble.org/mutant/database.php). *Arabidopsis thaliana* mutants *pht1;1*, *pht1;9*, *pho1*, *vpt1*, *irt1*, *sultr1;1*, *sultr1;2*, *sultr3;3/3;4/3;5*, *sultr3;1/3;2/3;3/3;5*, *sultr3;1/3;2/3;3/3;4/3;5*, *sultr4;1*, and *sultr4;2* were all in the Columbia‐0 (Col‐0) background. The overexpression plant *VPT1*‐OE and *MtVPT3*‐OE were based on Col‐0. Please see the Supporting Information for more details. PCR is used to screen homozygous individuals based on the primers listed in Table S1 (Supporting Information). Na_3_VO_4_ was used for the V treament in this study. The V treatment concentrations of *M. truncatula* and *A. thaliana* growing in soil were 0, 20, 200, and 2000 mg L^−1^, respectively, in vermiculite culture was 1000 mg L^−1^, for the hydroponic culture with ½‐strength Hoagland solution in HDAS was 30 or 100 mg L^−1^, and in ANS agar medium was 5 mg L^−1^.

Rhizosphere soil was brushed from the plant roots, collected, and frozen at −80 °C prior to rhizobacterial sequencing. After collecting the shoots and roots of the plants, they were dried, and 30 mg of the resulting sample powder was collected. After digestion, determinations were made of the concentrations of V, P, Fe, S, and Ca by inductively coupled plasma mass spectrometry (ICP‐OES) (ARCOS, SPECTRO Analytical Instruments GmbH, Germany). Fresh shoots of *irt1* and Col‐0 grown in vermiculite were obtained, from which samples of 100 mg were frozen and ground with liquid nitrogen for determination of Pi concentrations. The ascorbate‐molybdate‐antimony method was used for this determination, as previously described.^[^ ^10^ ^]^ The accumulation of ROS in plant tissues was detected by DAB and H2DCFDA staining. Chlorophyll fluorescence of leaves was measured using a kinetics multispectral fluorescence imaging system (Imaging‐PAM, WALZ GmbH, Germany) according to the manufacturer's instructions, and F_0_, F_m_ and F_v_/F_m_ ratios were analyzed. Fresh shoots and roots were collected from R108, *nsp1*, and *nsp2* plants grown on an inclined plane for RNA extraction. After reverse transcription into cDNA, real‐time quantitative PCR (RT‐qPCR) was performed, and *MtACTIN* (*MTR_3g095530*) was used as the internal control gene. The primers used for qPCR are listed in Table S1 (Supporting Information).

Then, 16S rRNA genes of distinct regions (V4‐V5) were amplified using specific primers (515F‐806R) with a barcode. The library was sequenced on an Illumina NovaSeq platform. Sequence analysis was performed using Uparse software (Uparse v7.0.1001, http://drive5.com/uparse/). Sequences with ≥97% similarity were assigned to the same operational taxonomic units (OTUs). A representative sequence for each OTU was screened for further annotation. The abundance information of OTUs was normalized using the sequence number corresponding to the sample with the fewest sequences as the standard. The subsequent analysis was carried out on the basis of the output normalized data. Please refer to the Supporting Information for detailed test methods and procedures.

### Statistical Analysis

All data were expressed as the mean ± SD, and statistically analyzed with SPSS 19.0 statistical software (IBM, Inc., Armonk, NY, USA) using Duncan's multiple range test *P* < 0.05 and independent samples t‐tests (* *P* < 0.05; ** *P* < 0.01; *** *P* < 0.001). β‐diversity was analyzed using principal coordinate analysis (PCoA) based on Bray–Curtis distance, and PERMANOVA was used to analyze significant differences. The construction of rhizobacterial co‐occurrence networks involved filtering out low‐frequency groups with a set screening threshold, standardized using counters per million, and performing Spearman correlation analysis between OTUs, thus identifying significant positive correlations (*ρ* > 0.7 and *p* < 0.05). Biomarkers were identified using Random Forests algorithm.

## Conflict of Interest

The authors declare no conflict of interest.

## Author Contributions

J.L. designed research. P.L., X.Z., L.L., Y.C., and X.L. performed research, with contributions made by L.Y. and J.Y. J.L., P.L., and X.Z. analyzed data. H.G. contributed new reagents/analytic tools. J.W. and K.S.M. constructed the *Tnt1*‐insertion mutants. J.L. and P.L. wrote the manuscript, with contributions made by X.Z. and L.L. J.L. acquired funding for this project.

## Supporting information

Supporting Information

## Data Availability

The data that support the findings of this study are available in the supplementary material of this article.
