# Supplementary material for: Nodulation Signaling Pathway 1 and 2 Modulate Vanadium Accumulation and Tolerance of Legumes
Source: Adv Sci (Weinh). 2024 Jan 15;11(12):2306389. doi: 10.1002/advs.202306389 (PMC10966554; doi:10.1002/advs.202306389)
Supplement: Supplementary file 1 — Supporting Information [file ADVS-11-2306389-s001.pdf]

ADVANCED SCIENCE
Open Access

Supporting Information

for *Adv. Sci.*, DOI 10.1002/advs.202306389

Nodulation Signaling Pathway 1 and 2 Modulate Vanadium Accumulation and Tolerance of Legumes

*Peng Liu, Xinfei Zhang, Lin Lin, Yanyan Cao, Xizhen Lin, Liaoliao Ye, Jun Yan, Huiling Gao, Jiangqi Wen, Kirankumar S. Mysore and Jinlong Liu**

Supporting Information

1. Materials and Methods

1.1. Plant Materials and Growth Conditions

The *Medicago truncatula* mutant *mtvpt3-1*, *Arabidopsis thaliana* mutant *vpt1*, and the overexpression plant *VPT1*-OE and *MtVPT3*-OE have been described in our previous studies.[1] The *A. thaliana* mutant *pht1;1* has been described by Liu et al. [2], *pht1;9* has been described by Remy et al. [3], *pho1* has been described by Hamburger et al.,[4] *irt1* has been described by Wang et al. [5], *sultr1;1* and *sultr1;2* have been described by Xu et al. [6], *sultr3;3/3;4/3;5*, *sultr3;1/3;2/3;3/3;5* and *sultr3;1/3;2/3;3/3;4/3;5* have been described by Chen et al. [7], and sultr4;1 and sultr4;2 have been described by Kataoka et al. [8].

Medicago truncatula seeds were sterilized with 75 % ethanol and washed five times using ddH_2O. After carefully cutting the seed coat with a scalpel, the seeds were transferred to a double-layer filter paper for vernalization at 4 °C for 2 days, and then germinated at 22 °C in dark. The germinated seedlings were transferred to sponge bars and floated on ½-strength Hoagland's nutrient solution for further growth. The plants were cultured under long-day conditions (16 h of illumination (150 μmol m^{-2} sec^{-1}) and 8 h of dark) at 22 °C and 50 % relative humidity in a growth chamber. For the analysis of rhizobacterial community, one-week-old seedlings with uniform growth were transferred to potting media containing a mixture of soil and vermiculite (2:1, v/v) as described by Durán et al. [9]. During growth, the plants were watered with ½-strength Hoagland nutrient solution twice and distilled water at other times. After nodulation, plants were treated with different concentrations of Na_3VO_4 (0, 20, 200, and 2000 mg L^{-1}) for one week, and then rhizosphere soil was obtained for microbiome determination. In order to analyze the dependence of vanadium (V) tolerance of wild-type R108 plants on soil microorganisms, autoclave and non-autoclave potting media were used. For strictly control the impact of microorganisms on plant V tolerance, 3-week-old wild-type (R108), *nsp1* and *nsp2* seedlings were cultured in a hydroponic device with an absorbent slope (HDAS) containing sterilized or unsterilized ½-strength Hoagland's nutrient solution formulated with soil extract.[10] After nodulation, plants were treated with 0 or 30 mg L^{-1} Na_3VO_4 for one week. For investigating the effect of vacuolar phosphate transporter mutations on plant V tolerance, one-week-old of wild-type (R108) and

mtvpt3-1 seedlings were transplanted into vermiculite media containing ½-strength Hoagland's nutrient solution and cultivated for 3 weeks before undergoing V treatment at concentrations of 0 and 1000 mg L^{-1}.

Arabidopsis thaliana was germinated according to the method described by Liu et al. [1a]. For investigating the effect of V stress on the shoot growth of *A. thaliana* P, Fe, and S transporter mutants, one-week-old seedlings were transferred to vermiculite, and cultured with ANS nutrient solution for 2 weeks, and then treated with or without 1000 mg L^{-1} Na_3VO_4 for one week.[1b] For investigating the effect of V stress on the root growth of *A. thaliana* phosphorus (P), iron (Fe), and sulfur (S) transporter mutants, 4-day-old seedlings were transferred to ANS agar medium with or without 5 mg L^{-1} Na_3VO_4 for 10 days.

1.2. Inorganic phosphorus (Pi) determination

Inorganic phosphorus concentration was measured using the ascorbate–molybdate–antimony method, as previously described.[11] Briefly, plant tissues were quickly ground into powder with liquid nitrogen. Pre-cooled 5 % perchloric acid (1 mL) was then added to the 0.1 g sample to extract Pi. The extract was centrifuged at 14000 g for 5 min, and then, the supernatant was used to determine Pi concentration. A reaction mixture containing 500 μL of sample and 10 mL of Pi detection agent (0.2 % ammonium molybdate, 0.005% potassium antimonyl tartrate, 2.2 % sulfuric acid, and 0.4 % ascorbate) was incubated at 36 °C for 15 min. Pi concentration was measured at 690 nm.

1.3. ROS determination

H_2O_2 was detected by DAB (3,3-diaminobenzidine) staining, as described previously. [12] Plant samples were vacuum infiltrated with 1 mg/ml DAB (50 mM Tris-acetate, pH 3.8) solution containing 0.05% (v/v) Tween 20 and incubated at 25 °C for 13 h in the dark. Next, the samples were rinsed in 80% (vol/vol) ethanol for 10 min at 70 °C and mounted in lactic acid/phenol/water (1:1:1, vol/vol/vol) before they were photographed using a DSLR camera. Intracellular ROS were detected using 2′-7′ dichlorodihydrofluorescein diacetate (H_2DCFDA, HY-D0940, MedChemExpress, USA). Briefly, the leaf samples were vacuum infiltrated with the staining solution (10 μM H_2DCFDA, 10 mM Tris-HCl, pH 7.2) for 5 min at 60 KPa pressure and incubated in the dark at room temperature for 25 min. Next, the samples were washed in three changes of autoclaved ddH_2O and mounted in 20% glycerol. Their fluorescence was then analyzed under a confocal microscope (Leica TCS SP8, Germany) at 488 nm excitation. ROS signals were detected at an emission of 500-550 nm. In addition, the chlorophyll autofluorescence was detected

at 640-700 nm.

1.4. RT qPCR analysis

Eastep Super Total RNA Extraction Kit (Promega, Shanghai, China) was used to extract total RNA from the samples. HiScript III 1st Strand cDNA Synthesis Kit (Vazyme, Nanjing, China) was used to synthesize first-strand cDNA with oligo (dT) primers. Real-time quantitative PCR (RT-qPCR) was performed using the ChamQ Universal SYBR qPCR Master Mix (Vazyme) on a LightCycler 480II (Roche, Basel, Switzerland). *MtACTIN* (MTR_3g095530) was used as the internal control. The primers used for qPCR are listed in Supplementary Table S1.

1.5. DNA extraction, PCR amplification and sequencing of rhizospheric bacteria

Total genome DNA from samples was extracted using CTAB/SDS method. DNA concentration and purity was monitored on 1 % agarose gels. According to the concentration, DNA was diluted to l ug/μL using sterile water.

16S rRNA genes of distinct regions (16S V4-V5) were amplified used specific primer (515F-806R) with the barcode. All PCR reactions were carried out with 15 μL of Phusion® High-Fidelity PCR Master Mix (New England Biolabs); 0.2 μM of forward and reverse primers, and about 10 ng template DNA. Thermal cycling consisted of initial denaturation at 98 °C for 1 min, followed by 30 cycles of denaturation at 98 °C for 10 s, annealing at 50°C for 30 s, and elongation at 72 °C for 30 s. Finally, 72 ℃ for 5 min.

Sequencing libraries were generated using TruSeq® DNA PCR-Free Sample Preparation Kit (Illumina, USA) following manufacturer's recommendations and index codes were added. The library quality was assessed on the Qubit 2.0 Fluorometer (Thermo Scientific) and Agilent Bioanalyzer 2100 system. At last, the library was sequenced on an Illumina NovaSeq platform and 250 bp paired-end reads were enerated.

1.6. Bioinformatics analysis on 16S rRNA gene profiling of rhizospheric bacteria

Sequence analysis was performed by Uparse software (Uparse v7.0.1001，http://drive5.com/uparse/). Sequences with a similarity of ≥ 97 % were assigned to the same OTUs. Representative sequence for each OTU was screened for further annotation. For each representative sequence, the Silva Database (http://www.arb-silva.de/) was used based on Mothur algorithm to annotate taxonomic

information. In order to study phylogenetic relationship of different OTUs, and the difference of the dominant species in different samples (groups), multiple sequence alignment were conducted using the MUSCLE software (Version 3.8.31 , http://www.drive5.com/muscle/). The abundance information of OTUs was normalized using the sequence number corresponding to the sample with the fewest sequences as the standard. Subsequent analysis of alpha diversity and beta diversity were performed based on this output normalized data. All data processing was done in R.4.2.1. The evaluation of α-diversity was conducted using four indices: the Shannon diversity index, Simpson's index, ACE, and Chao1. Additionally, β-diversity was analyzed using principal coordinate analysis (PCoA) based on Bray–Curtis distance, and PERMANOVA was used to analyze significant differences. The construction of rhizobacterial co-occurrence networks involved filtering out low-frequency groups with a set screening threshold, standardized using counters per million, and performing Spearman correlation analysis between OTUs, thus identifying significant positive correlations ($\rho > 0.7$ and $p < 0.05$). V-responsive OTUs in the modules were calculated and visualized, and Mantle tests were performed on the levels of V, P, Fe, S, and Ca in plants. Biomarkers were identified using Random Forests, and Spearman correlation analysis was performed between the biomarkers and plant element concentration.

1.6. Statistical analysis

All the data were statistically analyzed with SPSS 19.0 statistical software (IBM, Inc., Armonk, NY, USA) using Duncan's multiple range test $P < 0.05$ and independent samples t-tests (* $P < 0.05$; ** $P < 0.01$; *** $P < 0.001$).

References

[1] a) J. Liu, L. Yang, M. Luan, Y. Wang, C. Zhang, B. Zhang, J. Shi, F. G. Zhao, W. Lan, S. Luan, *Proc. Natl. Acad. Sci. USA.* **2015**, *112*, E6571; b) J. Liu, S. Fu, L. Yang, M. Luan, F. Zhao, S. Luan, W. Lan, *Plant Signal. Behav.* **2016**, *11*, e1213474; c) J. Liu, R. Yang, J. Yan, C. Li, X. Lin, L. Lin, Y. Cao, T. Xu, J. Li, Y. Yuan, J. Wen, K. S. Mysore, S. Luan, *Plant J.* **2023**, DOI: 10.1111/tpj.16363.

[2] Y. Liu, Y. Zhang, Z. Wang, S. Guo, Y. Fang, Z. Zhang, H. Gao, H. Ren, C. Wang, *Plant Physiol.* **2023**, *192*, 910.

[3] E. Remy, T. R. Cabrito, R. A. Batista, M. C. Teixeira, I. Sá-Correia, P. Duque, *New Phytol.* **2012**, *195*, 356.

[4] D. Hamburger, E. Rezzonico, J. M.-C. Petétot, C. R. Somerville, Y. Poirier, *Plant Cell.* **2002**, *14*, 889.

[5] Z. Wang, Y. Zhang, Y. Liu, D. Fu, Z. You, P. Huang, H. Gao, Z. Zhang, C. Wang, *Sci. China Life Sci.* **2023**, DOI: 10.1007/s11427-022-2330-4.

[6] Z. Xu, M.-L. Cai, S.-H. Chen, X.-Y. Huang, F.-j. Zhao, P. Wang, *Environ. Sci. Technol.* **2021**, 55, 1576.

[7] Z. Chen, P. X. Zhao, Z. Q. Miao, G. F. Qi, Z. Wang, Y. Yuan, N. Ahmad, M. J. Cao, R. Hell, M. Wirtz, C. B. Xiang, *Plant Physiol.* **2019**, *180*, 593.

[8] T. Kataoka, A. Watanabe-Takahashi, N. Hayashi, M. Ohnishi, T. Mimura, P. Buchner, M. J. Hawkesford, T. Yamaya, H. Takahashi, *Plant Cell.* **2004**, *16*, 2693.

[9] P. Durán, T. J. Ellis, T. Thiergart, J. Ågren, S. Hacquard, *New Phytol.* **2022**, *236*, 608.

[10] L. Ye, P. Yang, Y. Zeng, C. Li, N. Jian, R. Wang, S. Huang, R. Yang, L. Wei, H. Zhao, Q. Zheng, H. Gao, J. Liu, *J. Hazard. Mater.* **2021**, *415*, 125611.

[11] T. Y. Liu, T. K. Huang, S. Y. Yang, Y. T. Hong, S. M. Huang, F. N. Wang, S. F. Chiang, S. Y. Tsai, W. C. Lu, T. J. Chiou, *Nat. Commun.* **2016**, *7*, 11095.

[12] J. Liu, R. Yang, N. Jian, L. Wei, L. Ye, R. Wang, H. Gao, Q. Zheng, *Plant Cell Environ.* **2020**, *43*, 1348.

2. Supporting Figures

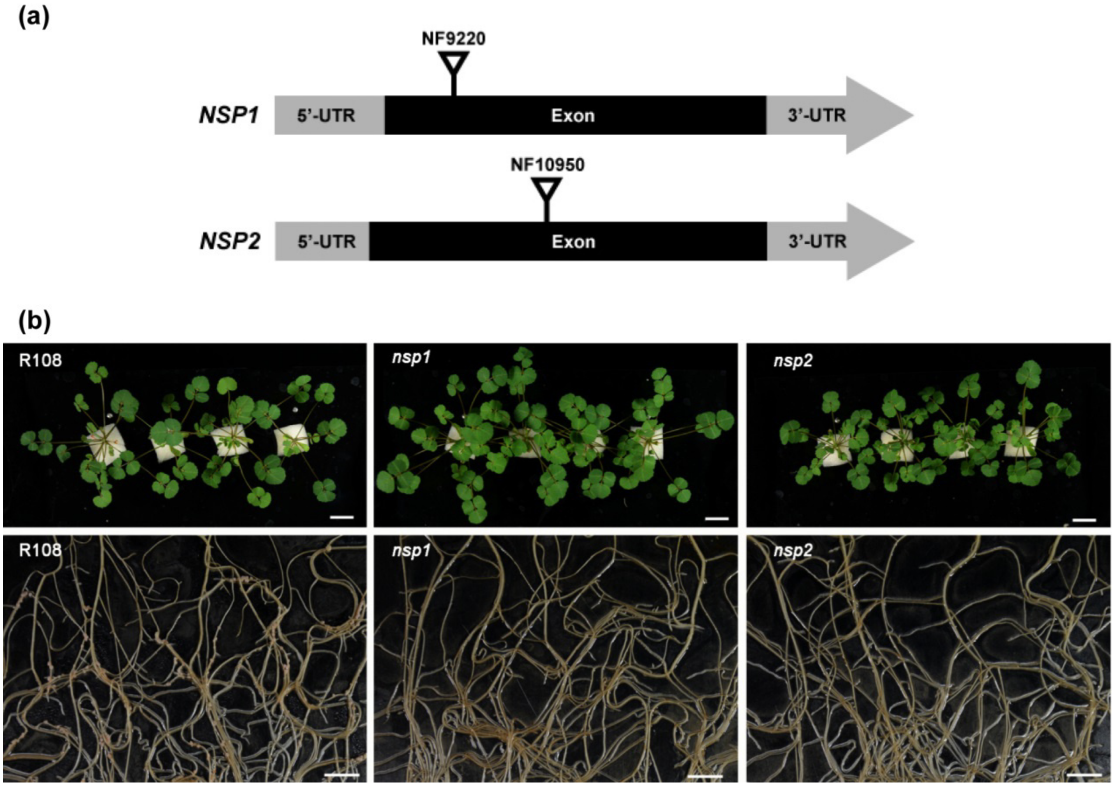

Figure S1. The *Rhizobium* symbiosis is defective in *nsp1* and *nsp2* mutant plants.

(a) Diagrams of *Tnt1*-insertions in the *NSP1* and *NSP2* genes of the mutant lines *nsp1* (NF9220) and *nsp2* (NF10950). The gray and black boxes represent the 5'-UTR and exon, respectively. The gray arrow indicates the 3'-UTR.

(b) Phenotypes of shoots and root nodules of wild-type (R108), *nsp1*, and *nsp2* plants. Three-week-old seedlings were inoculated with *Rhizobium meliloti* 1021 for one week. Bars, 2 cm.

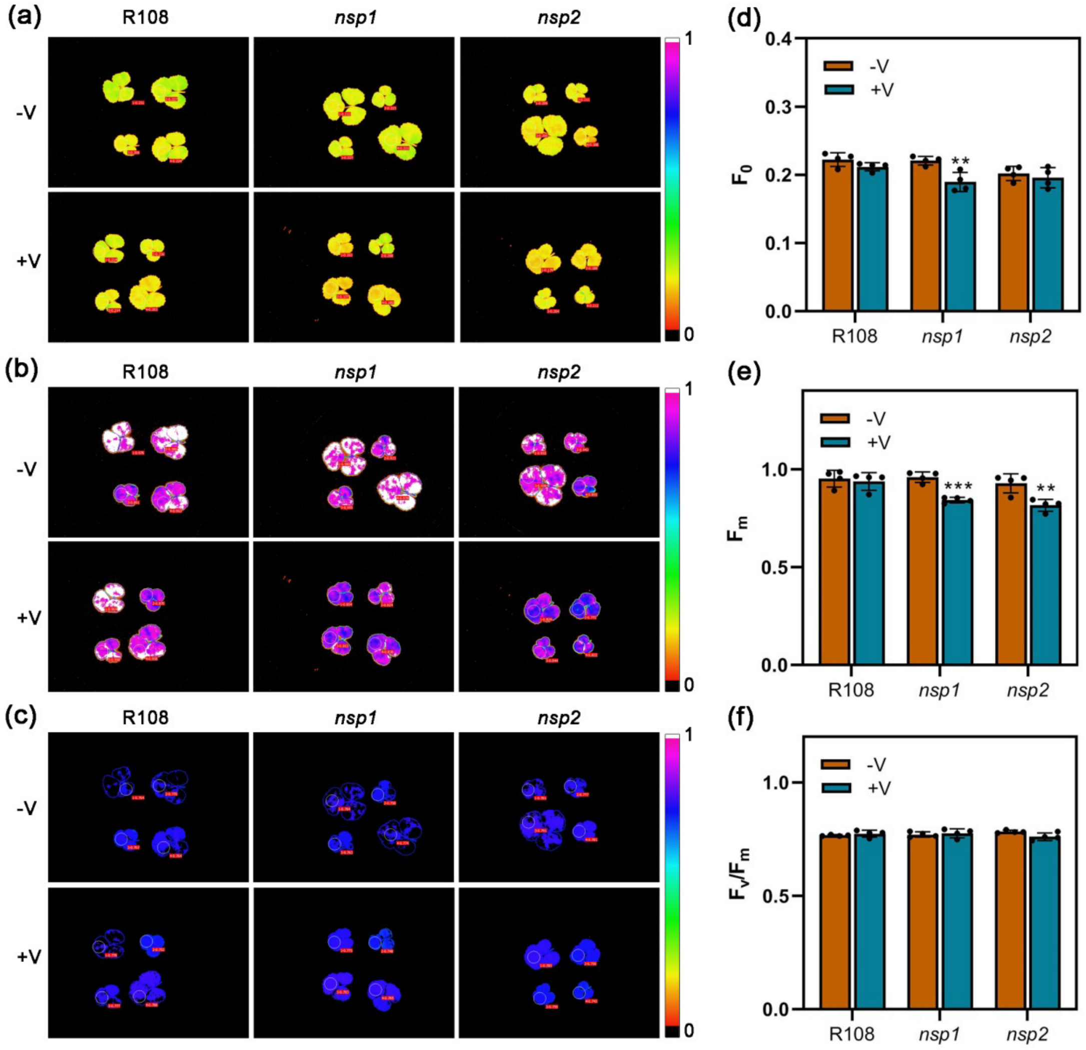

Figure S2. *NSP1* and *NSP2* mutations increase the sensitivity of chlorophyll fluorescence to vanadium (V) stress.

(a-c) Images of F0 (a), Fm (b), and Fv/Fm ratios (c) in the leaves of wild type (R108) and mutant *nsp1* and *nsp2 Medicago truncatula* plants. Three-week-old seedlings were cultured with ½-strength Hoagland nutrient solution prepared with soil extract. After nodulation of R108, plants were treated with (+V) or without (-V) 100 mg L^{-1} V for 16 h. Then, leaves were obtained for chlorophyll fluorescence imaging.

(d-f) Statistical analysis of F0 (d), Fm (e), and Fv/Fm ratios (f) in (a-c). Data are expressed as mean ± SD values ($n = 4$). Statistical significance is denoted by asterisks based on independent sample *t*-tests (** $P < 0.01$, *** $P < 0.001$).

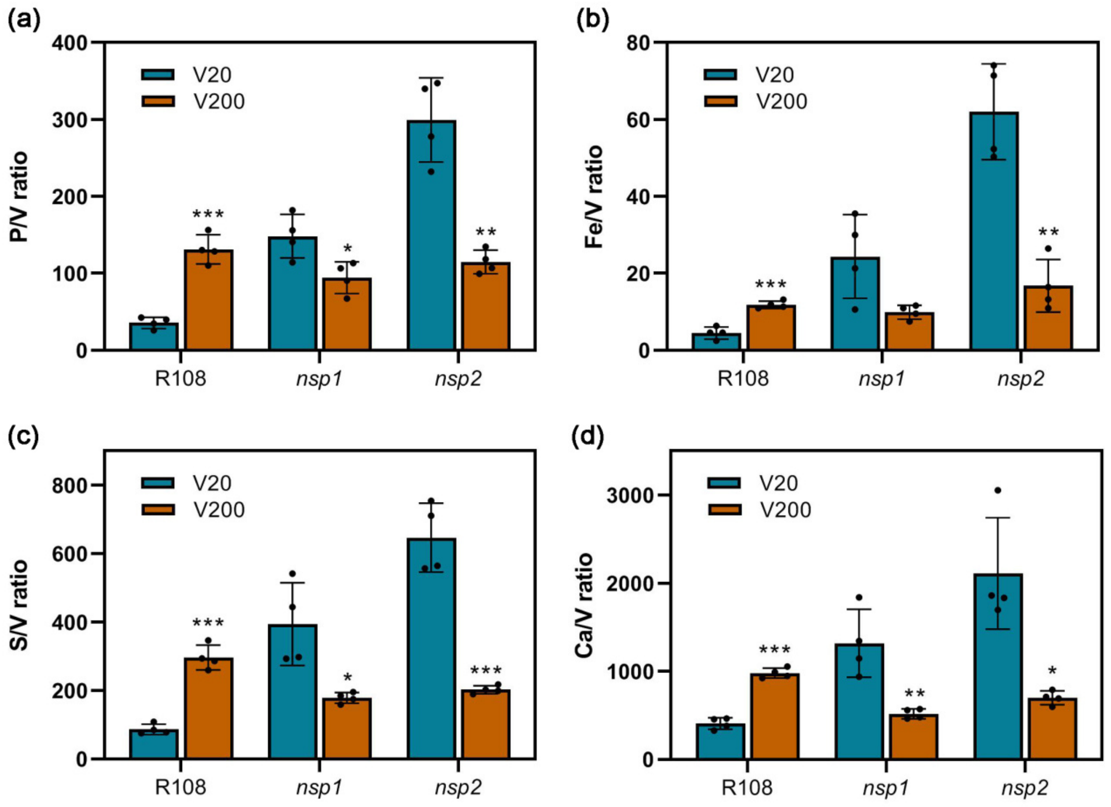

Figure S3. *NSP1* and *NSP2* mutations reverse the increase in the ratio of nutrient elements to vanadium (V) caused by V stress increase.

The P/V (a), Fe/V (b), S/V (c), and Ca/V (d) ratios in the shoots of 5-week-old wild-type (R108), *nsp1*, and *nsp2 Medicago truncatula* plants treated with 20 mg L^{-1} (V20) or 200 mg L^{-1} (V200) for one week. Data were calculated from the ion concentrations in Figure 2. Data are expressed as mean ± SD values ($n = 4$). Statistical significance is denoted by asterisks based on independent sample *t*-tests (* $P < 0.05$, ** $P < 0.01$, *** $P < 0.001$).

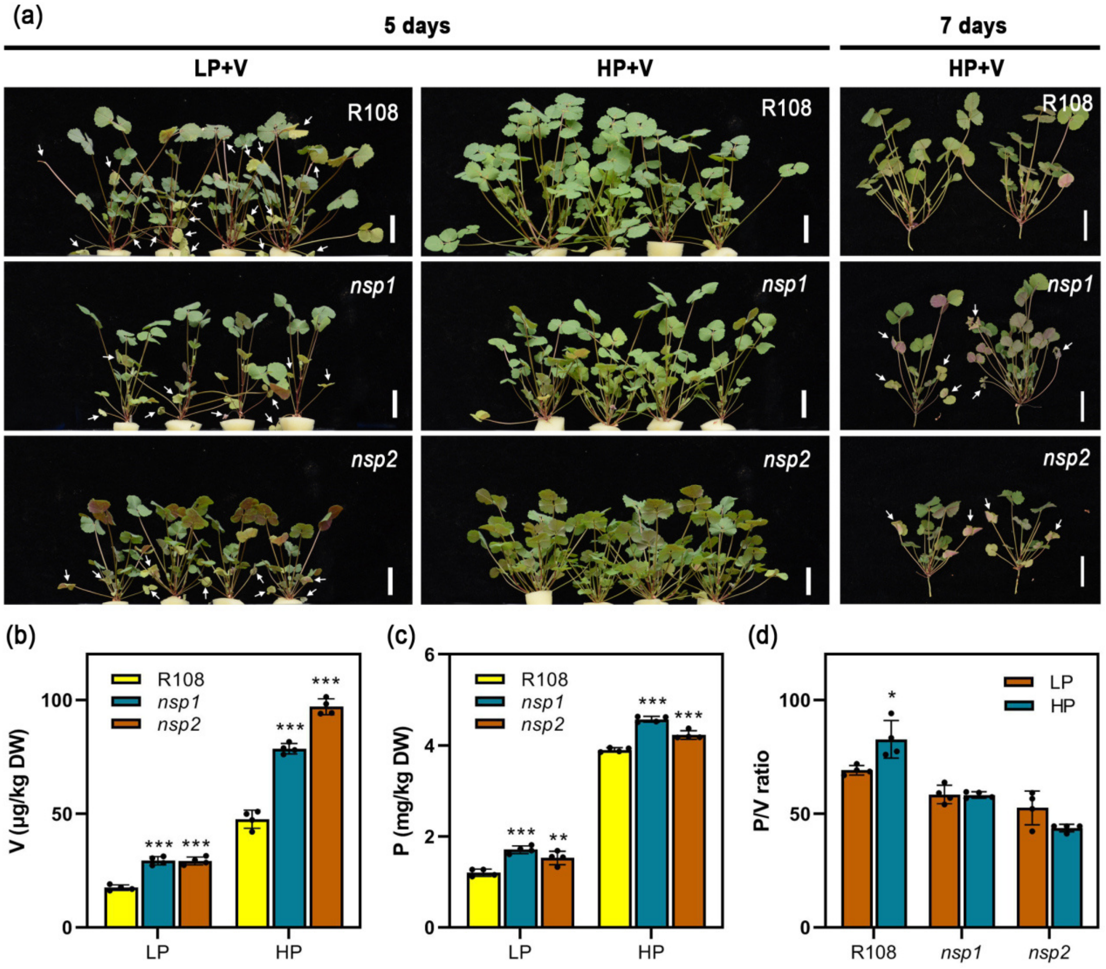

Figure S4. Effects of different phosphorus (P) conditions on vanadium (V) tolerance and ion accumulation in plants.

(a) Growth phenotypes of wild-type (R108) and mutant *nsp1* and *nsp2 Medicago truncatula* plants induced by V stress under low or high P conditions. Three-week-old seedlings were cultured with ½-strength Hoagland nutrient solution prepared with soil extract. After nodulation of R108, plants were treated with 30 mg L^{-1} V under low P (LP, 1 μM PO_4^{3-}) or high P (HP, 1 mM PO_4^{3-}) conditions for 5 or 7 days. Arrows indicate plant leaves that curl or fall after death. Bars, 2 cm.

(b,c) Concentrations of V (b) and P (c) in the shoots of wild-type (R108) and mutant *nsp1* and *nsp2* plants after 5 days of V treatment described in (a).

(d) The P/V ratio in the shoots of R108, *nsp1*, and *nsp2* plants. Data were calculated from P and V concentrations in (b,c). Data are expressed as mean ± SD values (n = 4). Statistical significance is denoted by asterisks based on independent sample t-tests (* $P < 0.05$, ** $P < 0.01$, *** $P < 0.001$).

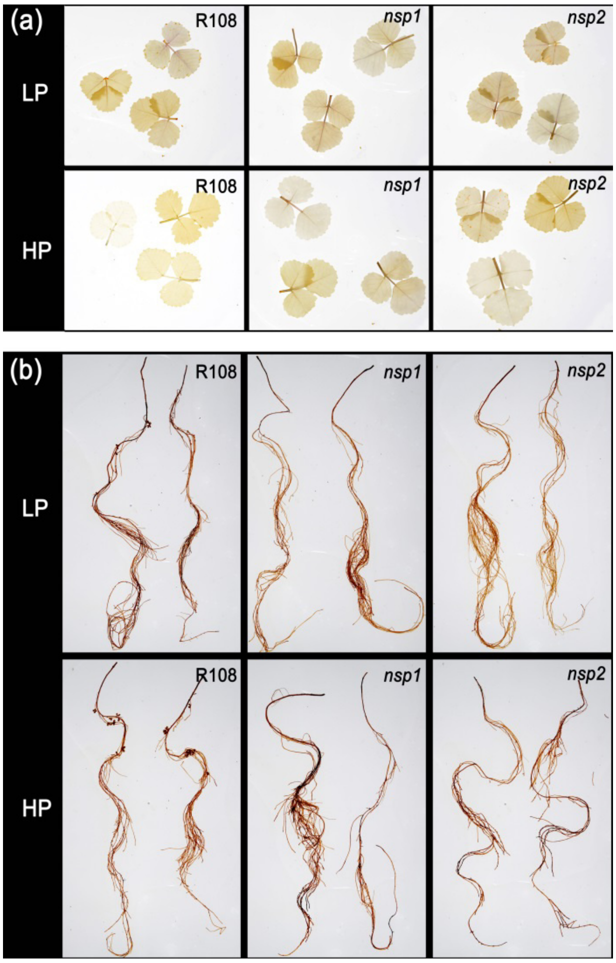

Figure S5. ROS accumulation in plants exposed to vanadium (V) stress under different phosphorus (P) conditions.

ROS accumulation in the leaves (a) and roots (b) of wild-type (R108) and mutant *nsp1* and *nsp2 Medicago truncatula* plants. Three-week-old seedlings were cultured with ½-strength Hoagland nutrient solution prepared with soil extract. After nodulation of R108, plants were treated with 30 mg L^{-1} V under low P (LP, 1 μM PO_4^{3-}) or high P (HP, 1 mM PO_4^{3-}) conditions for 5 days. Then, leaves and roots were obtained for DAB staining.

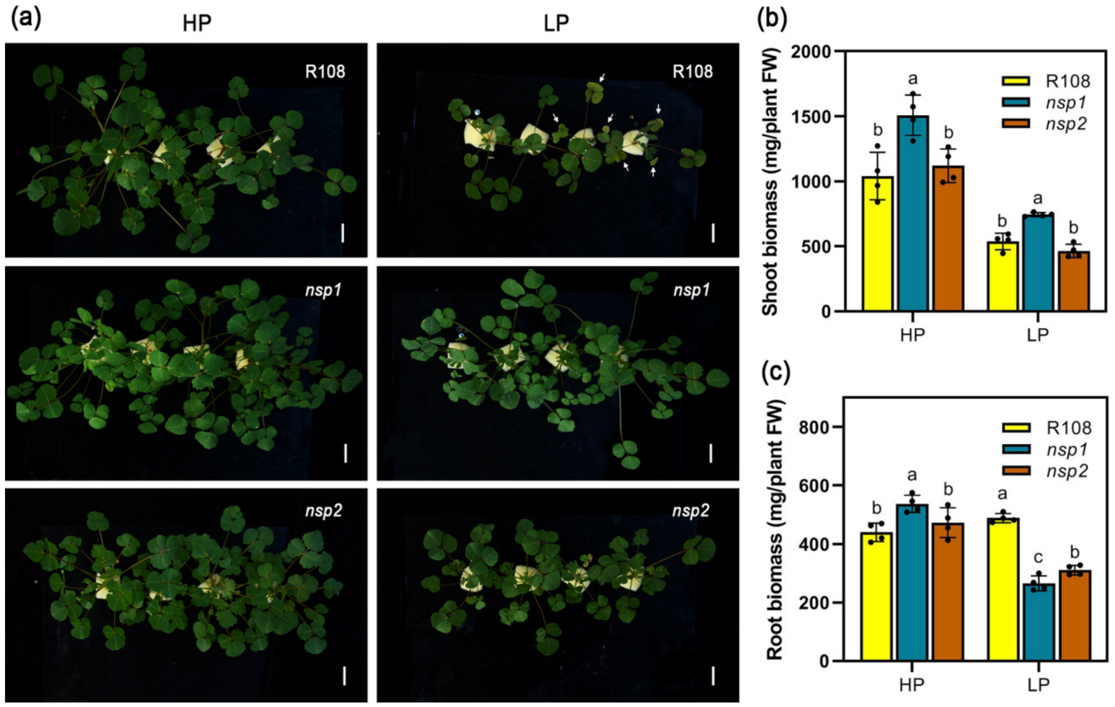

Figure S6. *NSP1* and *NSP2* mutations increase low phosphorus (P) tolerance of *Medicago truncatula*.

(a) Growth phenotypes of wild-type (R108) and mutant *nsp1* and *nsp2* plants under high or low P conditions. Three-week-old seedlings were cultured with ½-strength Hoagland nutrient solution prepared with soil extract. After nodulation of R108, plants were treated with high P (HP, 1 mM PO_4^{3-}) or low P (LP, 1 μM PO_4^{3-}) for 7 days. Arrows indicate dead and yellow leaves. Bars, 2 cm.

(b,c) Shoot (b) and root (c) biomass of R108, *nsp1*, and *nsp2* plants under high or low P conditions. Plants were treated as described in (a). Data are expressed as mean ± SD values (n = 4). Different letters above the bars indicate significant differences at P < 0.05 (Duncan's test).

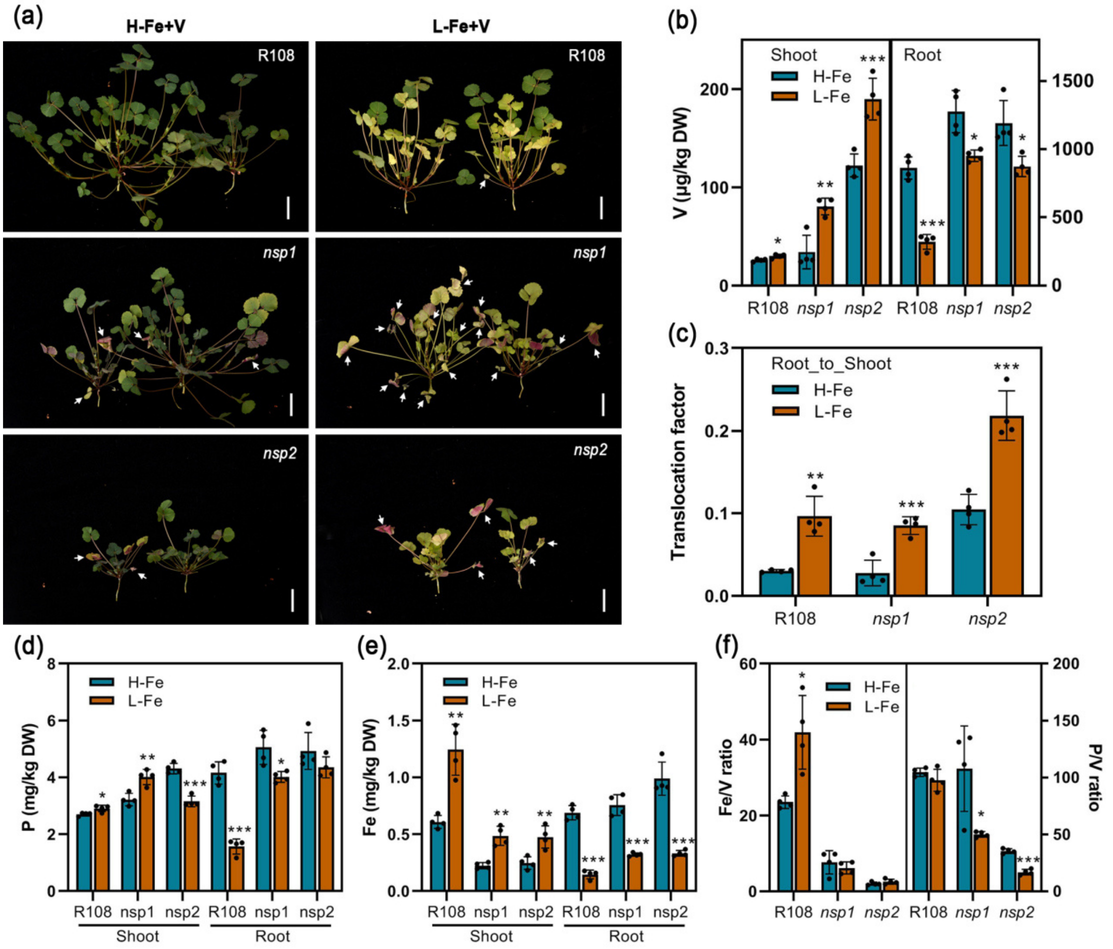

Figure S7. Effects of different iron (Fe) conditions on vanadium (V) tolerance and ion accumulation in plants.

(a) Growth phenotypes of wild-type (R108) and mutant *nsp1* and *nsp2 Medicago truncatula* plants induced by V stress under low or high Fe conditions. Three-week-old seedlings were cultured with ½-strength Hoagland nutrient solution prepared with soil extract. After nodulation of R108, plants were treated with 30 mg L^{-1} V under low Fe (L-Fe, 1 μM Fe^{2+}) or high Fe (H-Fe, 89.6 μM Fe^{2+}) conditions for 5 or 7 days. Arrows indicate plant leaves that curl or fall after death. Bars, 2 cm.

(b) The V concentration in the shoots and roots of wild-type (R108) and mutant *nsp1* and *nsp2* plants after 5 days of V treatment described in (a).

(c) The translocation factor of V from roots to shoots of R108, *nsp1*, and *nsp2* plants after 5 days of V treatment described in (a). Data were calculated from shoot-to-root ratios of V concentrations in (b).

(d,e) Concentrations of P (d) and Fe (e) in the shoots and roots of wild-type (R108) and mutant *nsp1* and *nsp2* plants after 5 days of V treatment described in (a).

(f) The Fe/V and P/V ratios in the shoots of R108, *nsp1*, and *nsp2* plants. Data were calculated from P, Fe, and V concentrations in (b,d,e). Data are expressed as mean ± SD values (n = 4). Statistical significance is denoted by asterisks based on independent sample t-tests (* $P < 0.05$, ** $P < 0.01$, *** $P < 0.001$).

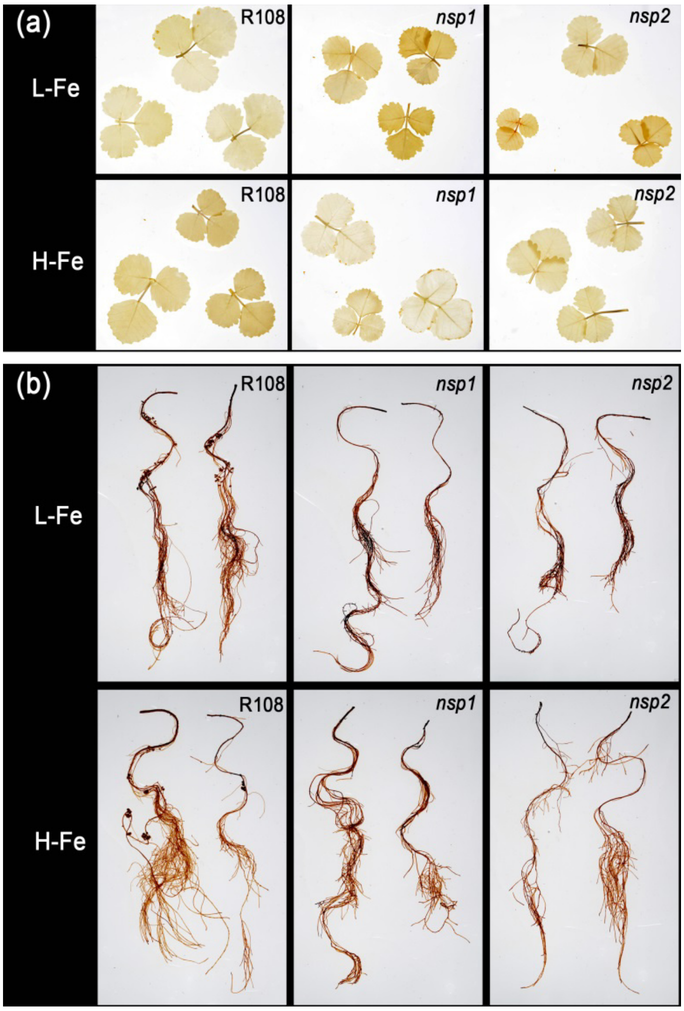

Figure S8. ROS accumulation in plants exposed to vanadium (V) stress under different iron (Fe) conditions.

ROS accumulation in the leaves (a) and roots (b) of wild-type (R108) and mutant *nsp1* and *nsp2 Medicago truncatula* plants. Three-week-old seedlings were cultured with ½-strength Hoagland nutrient solution prepared with soil extract. After nodulation of R108, plants were treated with 30 mg L^{-1} V under low Fe (L-Fe, 1 μM Fe^{2+}) or high Fe (H-Fe, 89.6 μM Fe^{2+}) conditions for 5 days. Then, leaves and roots were obtained for DAB staining.

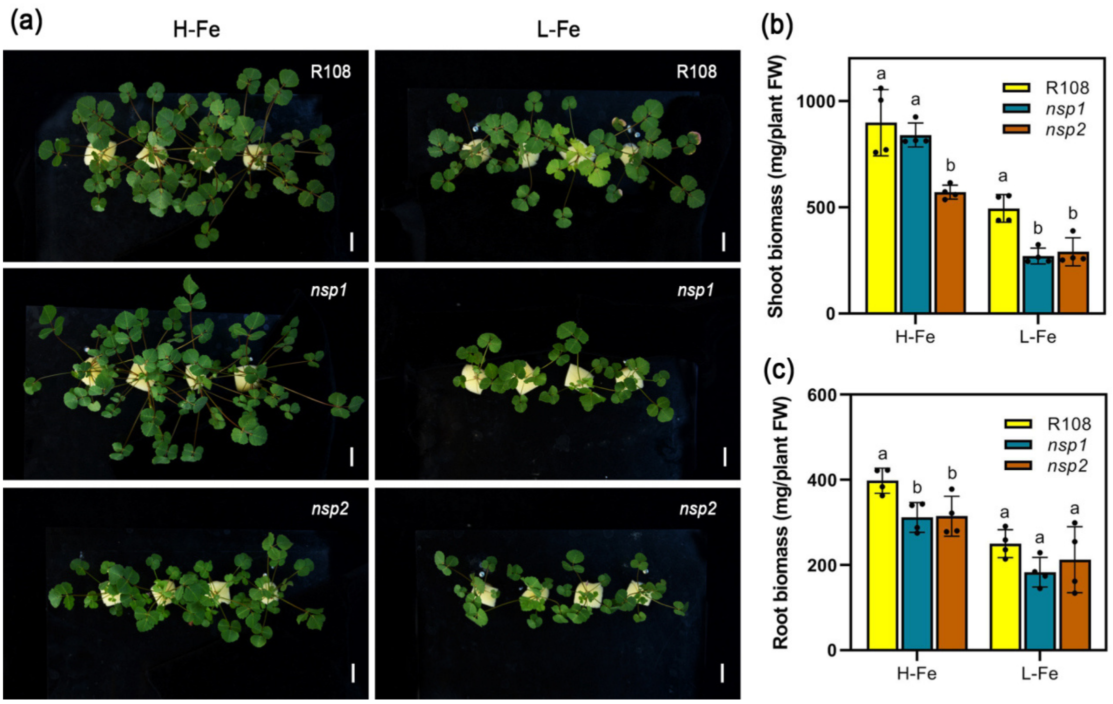

Figure S9. Effect of *NSP1* and *NSP2* mutations on iron (Fe) adaptation of *Medicago truncatula*.

(a) Growth phenotypes of wild-type (R108) and mutant *nsp1* and *nsp2* plants under high or low Fe conditions. Three-week-old seedlings were cultured with ½-strength Hoagland nutrient solution prepared with soil extract. After nodulation of R108, plants were treated with high Fe (H-Fe, 89.6 μM Fe^{2+}) or low Fe (L-Fe, 1 μM Fe^{2+}) for 7 days. Bars, 2 cm.

(b,c) Shoot (b) and root (c) biomass of R108, *nsp1*, and *nsp2* plants under high or low Fe conditions. Plants were treated as described in (a). Data are expressed as mean ± SD values (n = 4). Different letters above the bars indicate significant differences at $P < 0.05$ (Duncan's test).

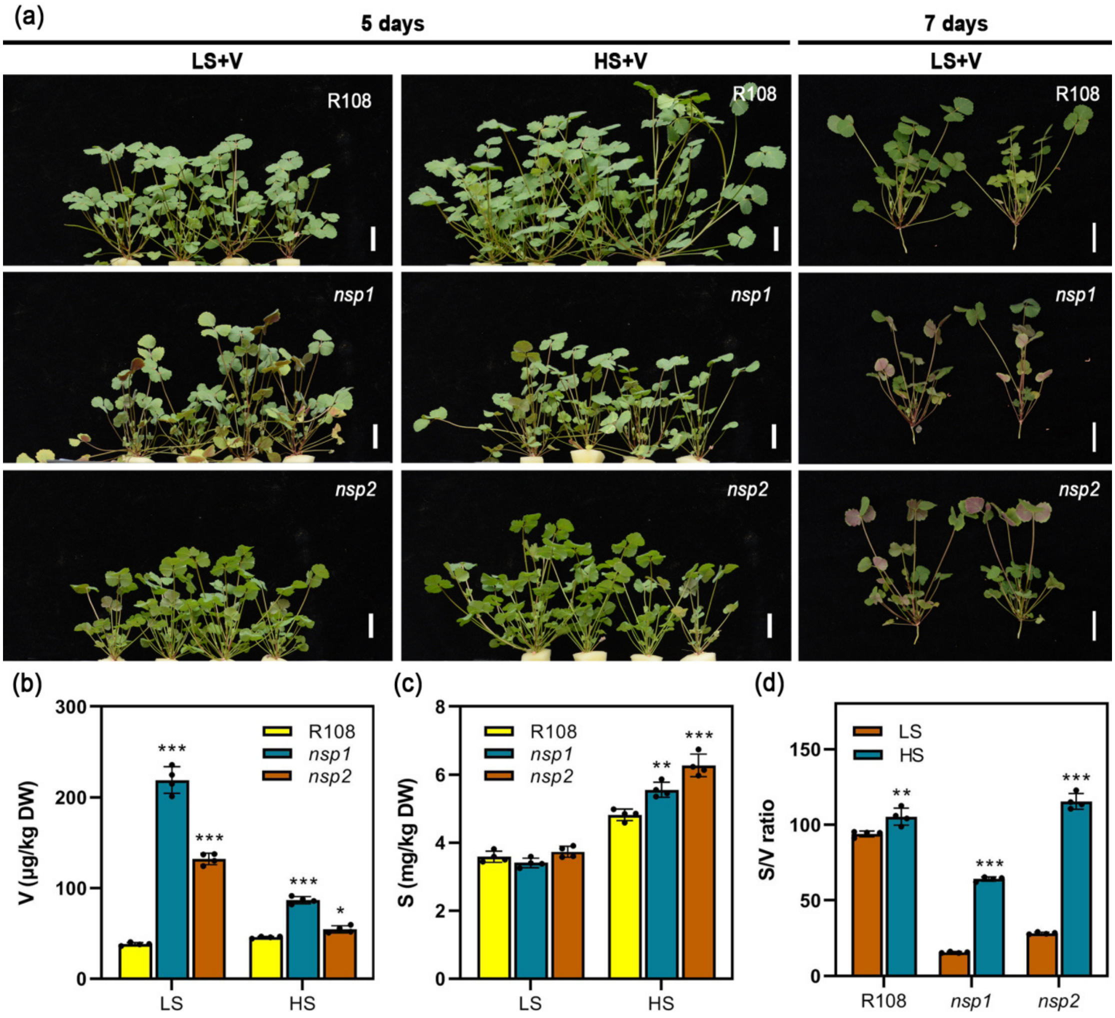

Figure S10. Effects of different sulfur (S) conditions on vanadium (V) tolerance and ion accumulation in plants.

(a) Growth phenotypes of wild-type (R108) and mutant *nsp1* and *nsp2 Medicago truncatula* plants induced by V stress under low or high S conditions. Three-week-old seedlings were cultured with ½-strength Hoagland nutrient solution prepared with soil extract. After nodulation of R108, plants were treated with 30 mg L^{-1} V under low S (LP, 1 µM SO_4^{2-}) or high S (HP, 5 mM SO_4^{2-}) conditions for 5 or 7 days. Bars, 2 cm.

(b,c) Concentrations of V (b) and S (c) in the shoots of wild-type (R108) and mutant *nsp1* and *nsp2* plants after 5 days of V treatment described in (a).

(d) The S/V ratio in the shoots of R108, *nsp1*, and *nsp2* plants. Data were calculated from S and V concentrations in (b,c). Data are expressed as mean ± SD values ($n = 4$). Statistical significance is denoted by asterisks based on independent sample *t*-tests (* $P < 0.05$, ** $P < 0.01$, *** $P < 0.001$).

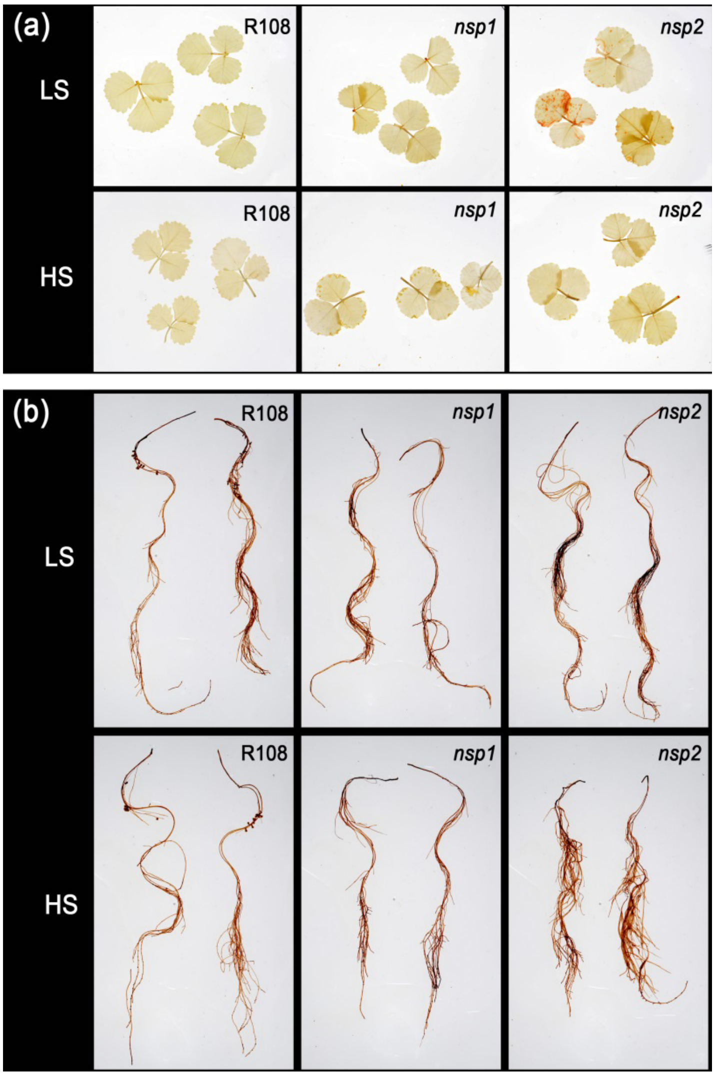

Figure S11. ROS accumulation in plants exposed to vanadium (V) stress under different sulfur (S) conditions.

ROS accumulation in the leaves (a) and roots (b) of wild-type (R108) and mutant *nsp1* and *nsp2 Medicago truncatula* plants. Three-week-old seedlings were cultured with ½-strength Hoagland nutrient solution prepared with soil extract. After nodulation of R108, plants were treated with 30 mg L^{-1} V under low S (LS, 1 μM SO_4^{2-}) or high S (HS, 5 mM SO_4^{2-}) conditions for 5 days. Then, leaves and roots were obtained for DAB staining.

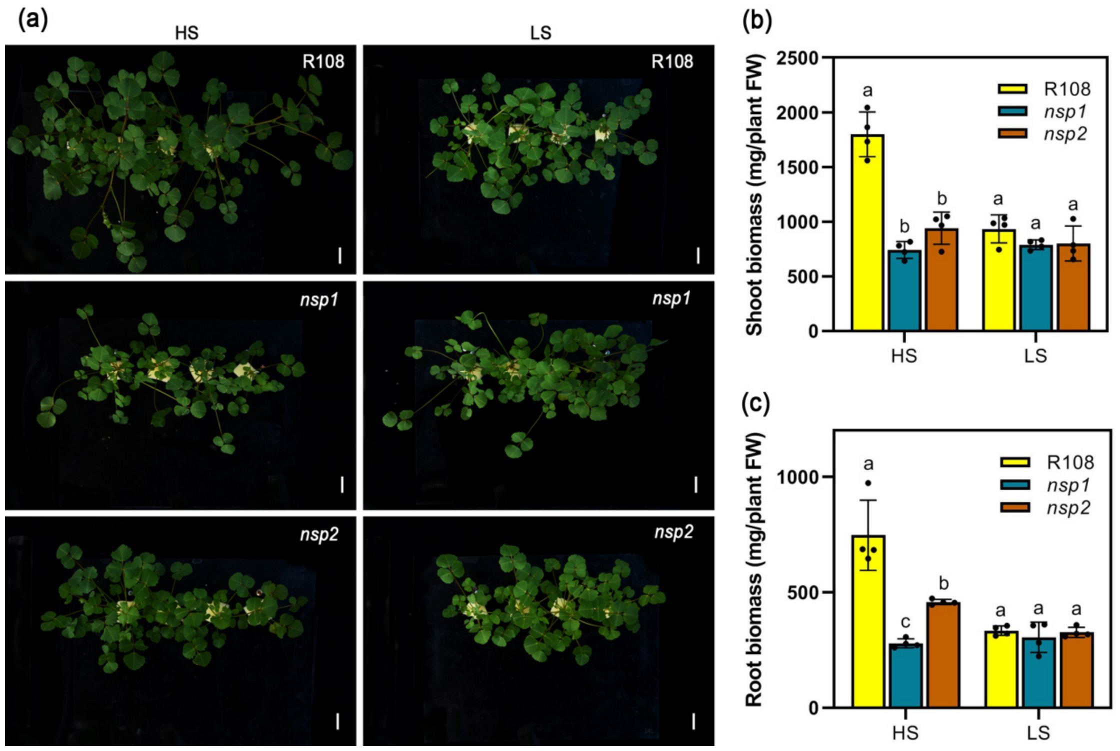

Figure S12. Effect of *NSP1* and *NSP2* mutations on sulfur (S) adaptation of *Medicago truncatula*.

(a) Growth phenotypes of wild-type (R108) and mutant *nsp1* and *nsp2* plants under high or low S conditions. Three-week-old seedlings were cultured with ½-strength Hoagland nutrient solution prepared with soil extract. After nodulation of R108, plants were treated with high S (HS, 5 mM SO_4^{2-}) or low S (LS, 1 μM SO_4^{2-}) for 7 days. Bars, 2 cm.

(b,c) Shoot (b) and root (c) biomass of R108, *nsp1*, and *nsp2* plants under high or low S conditions. Plants were treated as described in (a). Data are expressed as mean ± SD values (n = 4). Different letters above the bars indicate significant differences at P < 0.05 (Duncan's test).

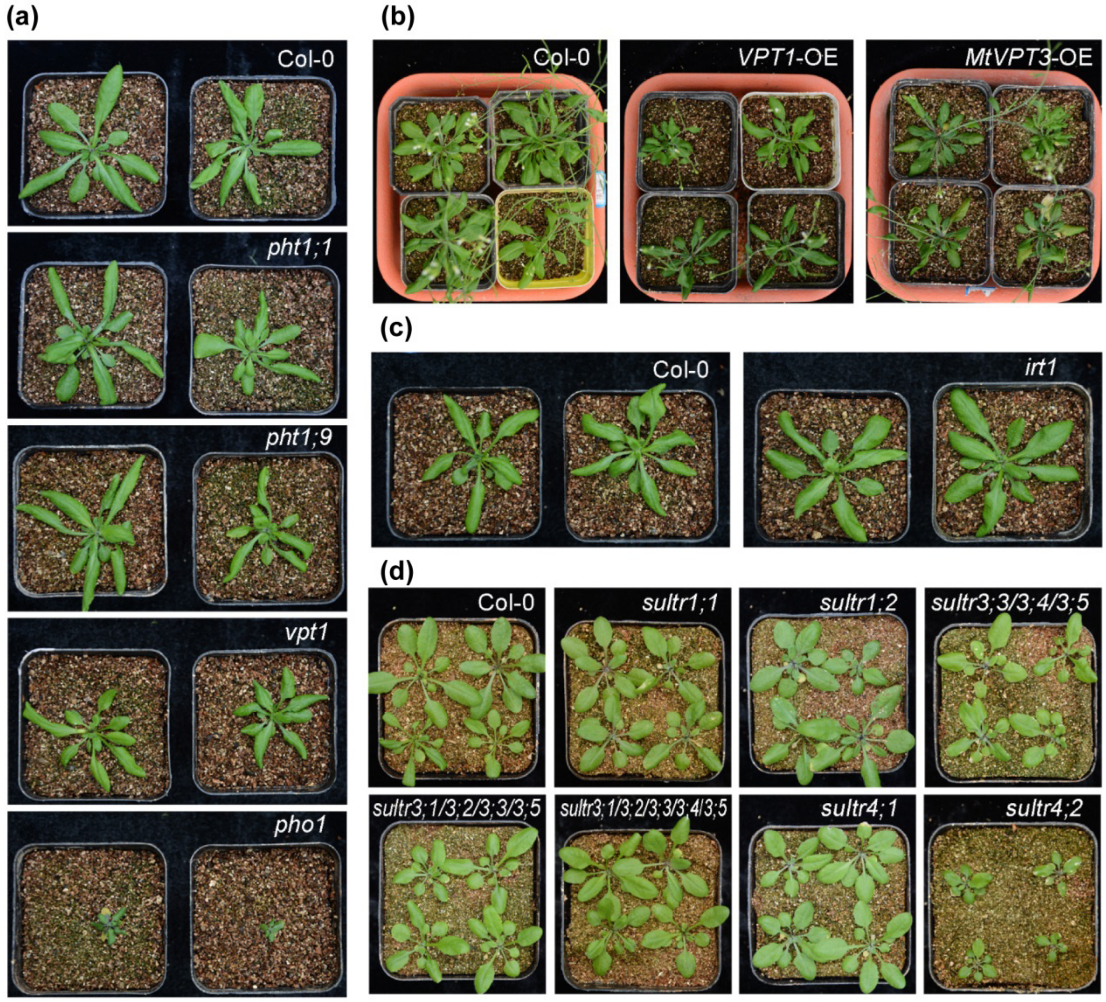

Figure S13. Growth phenotypes of *Arabidopsis thaliana* wild-type (Col-0), mutant, and overexpressing plants under normal conditions.

(a) Growth phenotypes of wild-type (Col-0) and phosphate transporter mutant (*pht1;1*, *pht1;9*, *vpt1*, and *pho1*) plants.

(b) Growth phenotypes of wild-type (Col-0), *VPT1*-overexpressing (*VPT1-OE*), and *MtVPT3*-overexpressing (*MtVPT3-OE*) plants.

(c) Growth phenotypes of wild-type (Col-0), iron transporter mutant *irt1* plants.

(d) Growth phenotypes of wild-type (Col-0), sulfate transporter mutant (*sultr1;1*, *sultr1;2*, *sultr3;3/3;4/3;5*, *sultr3;1/3;2/3;3/3;5*, *sultr3;1/3;2/3;3/3;4/3;5*, *sultr4;1*, and *sultr4;2*) plants.

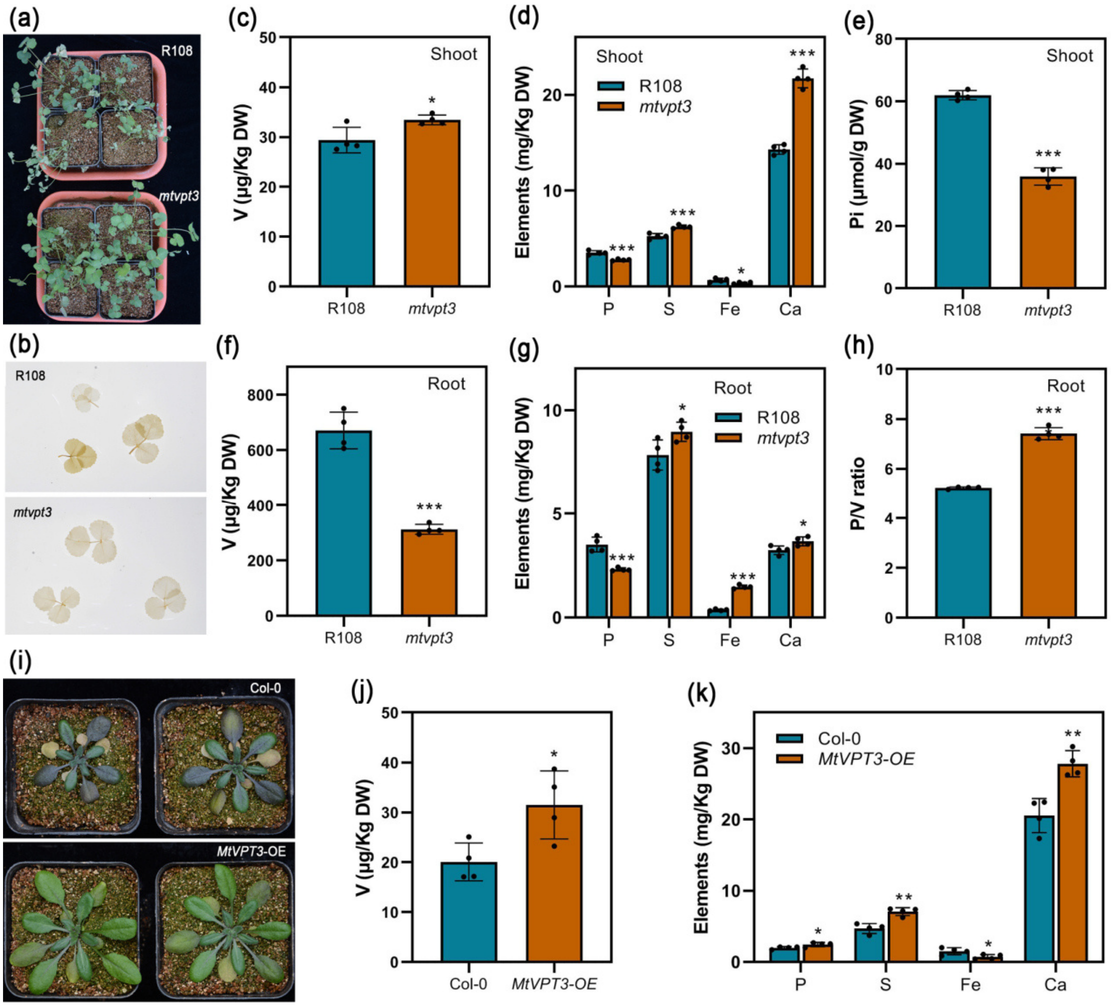

Figure S14. Effects of mutation and overexpression of *MtVPT3* on element accumulation in plants under vanadium (V) stress.

(a) Growth phenotypes of *Medicago truncatula* wild-type (R108) plants and the vacuolar phosphate transporter mutant *mtvpt3* plants under V stress. Five-week-old seedlings were treated with 1000 mg L^{-1} V for five days.

(b) ROS accumulation in the leaves of wild-type (R108) and *mtvpt3* mutant plants under V stress. Plants were treated as described in (a). Then, leaves were obtained for DAB staining.

(c-e) Concentrations of total V (c), P, S, Fe, and Ca (d) and inorganic P (Pi) (e) in shoots of wild-type (R108) and *mtvpt3* mutant plants described in (a).

(f,g) Concentrations of total V (f), P, S, Fe, and Ca (g) in roots of wild-type (R108) and *mtvpt3* mutant plants described in (a).

(h) The P/V ratio in the roots of wild-type (R108) and *mtvpt3* mutant plants described in (a). Data were calculated from P and V concentrations in (f,g).

(i) Growth phenotypes of *Arabidopsis thaliana* wild-type (Col-0) and *MtVPT3*-overexpressing plants (*MtVPT3*-OE) under V stress. Three-week-old seedlings were treated with 1000 mg L^{-1} V for 10 days.

(j,k) Concentrations of total V (j), P, S, Fe, and Ca (k) in shoots of wild-type (Col-0) and *MtVPT3*-OE plants described in (a).

Data are expressed as mean ± SD values ($n = 4$). Statistical significance is denoted by asterisks based on independent sample *t*-tests (* $P < 0.05$, ** $P < 0.01$, *** $P < 0.001$).

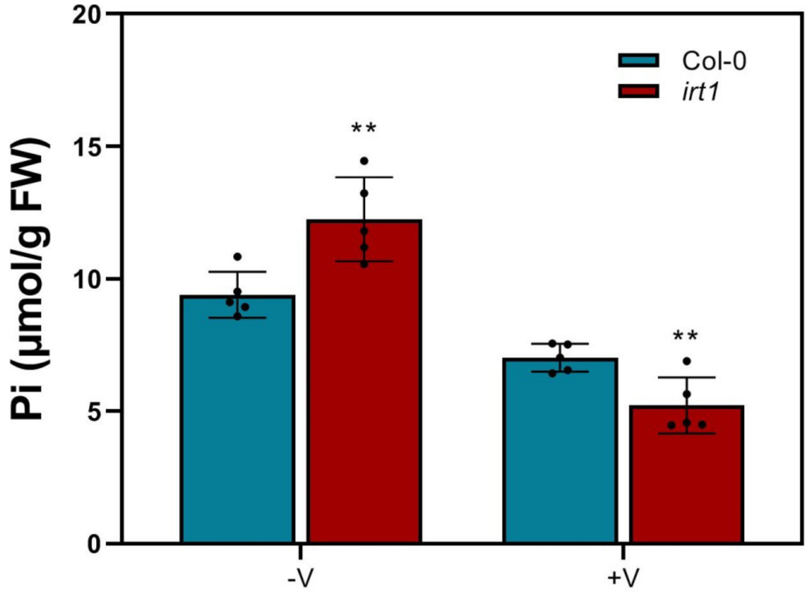

Figure S15. The *IRT1* mutation alters the Inorganic phosphorus (Pi) concentrations in the shoot of *Arabidopsis thaliana*.

Three-week-old seedlings grown in vermiculite media were treated with (+V) or without (-V) 1000 mg L^{-1} VO_4^{3-} for one week. Data are presented as mean ± SD. $n = 5$. Asterisks denote statistically significant differences according to independent sample *t*-tests (** $P < 0.01$).

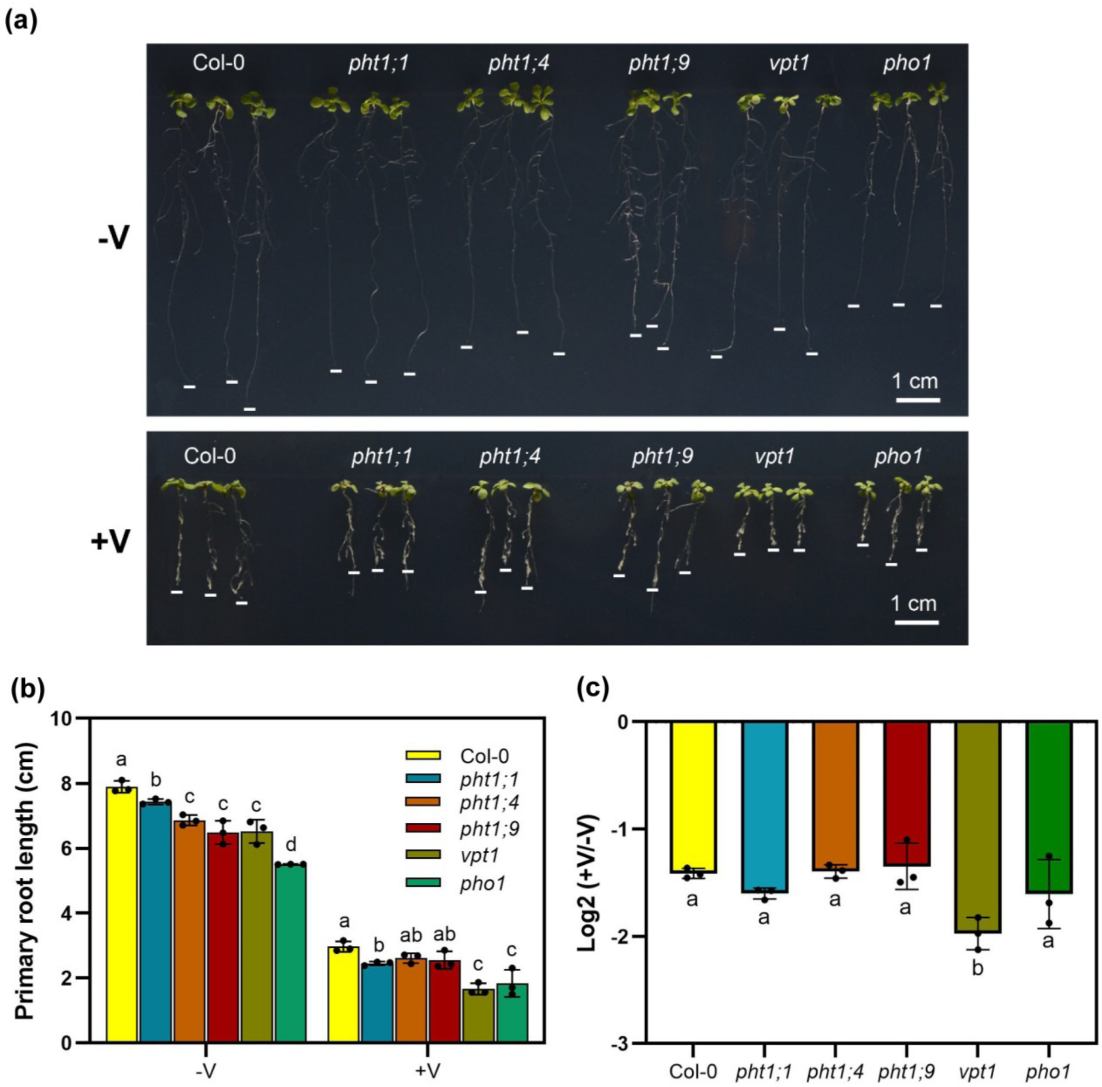

Figure S16. The impact of vanadium (V) stress on the root growth of *Arabidopsis thaliana* phosphate transporter mutants.

(a) Growth phenotype of two-week-old wild-type (Col-0), *pht1;1*, *pht1;4*, *pht1;9*, *vpt1*, and *pho1* mutant plants under V stress. Seedlings were cultured on ANS agar medium with (+V) or without (-V) 5 mg L^{-1} VO_4^{3-}. Bars, 1 cm.

(b) Primary root length of wild-type (Col-0) and phosphate transporter mutant plants as described in (a). The data are expressed as mean ± SD. $n = 4$.

(c) The change of primary root length under V stress. The primary root length under normal conditions (-V) was used as the control. The log2 of the ratio of each primary root length under V stress (+V) divided by the value of control was calculated. Data are presented as mean ± SD. $n = 4$. Different letters above the bars represent significant differences at $P < 0.05$ (Duncan's test).

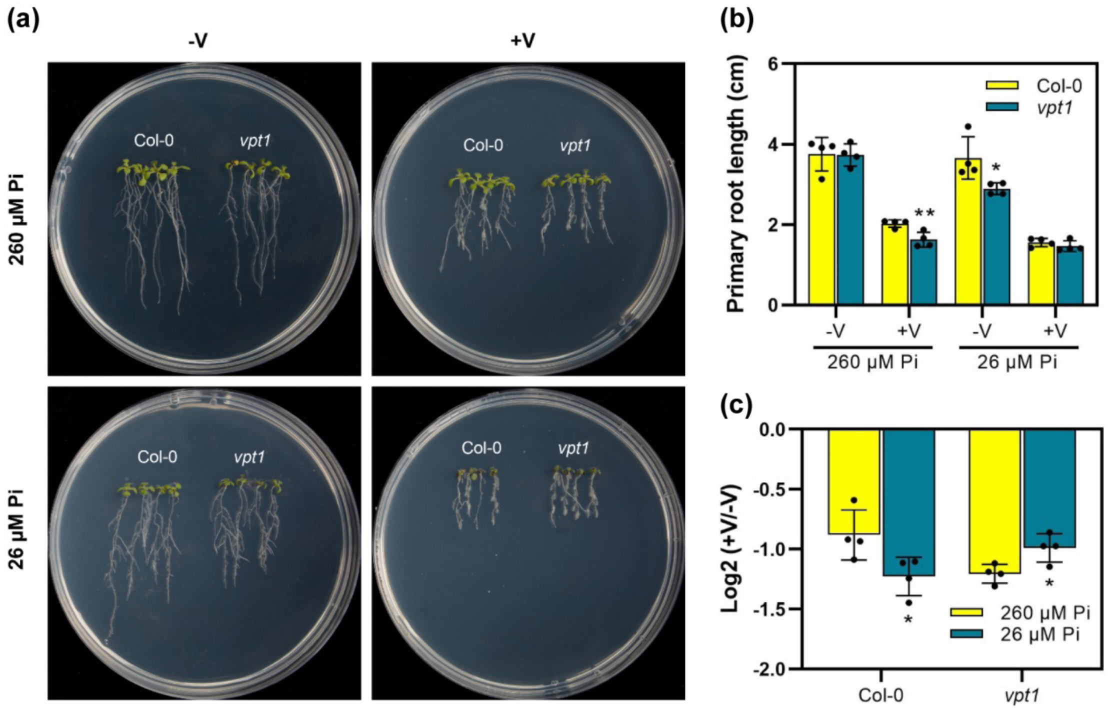

Figure S17. Growth phenotype of the phosphorus (P) transporter mutant *vpt1* under vanadium (V) stress.

(a) Growth phenotype of 2-week-old wild-type (Col-0) and *vpt1* mutant plants under different P and V conditions. Seedlings were cultured on ANS agar medium containing 260 or 26 μM PO_4^{3-} (Pi), and 0 (-V) or 5 mg L^{-1} VO_4^{3-} (+V).

(b) Primary root length of wild-type (Col-0) and *vpt1* seedlings under different P and V conditions as described in (a). The data are expressed as mean ± SD. $n = 4$.

(c) The change of primary root length under V stress. The primary root length under normal conditions (-V) was used as the control. The log2 of the ratio of each primary root length under V stress (+V) divided by the value of control was calculated. Data are presented as mean ± SD. $n = 4$. Asterisks denote statistically significant differences according to independent sample *t*-tests (* $P < 0.05$, ** $P < 0.01$).

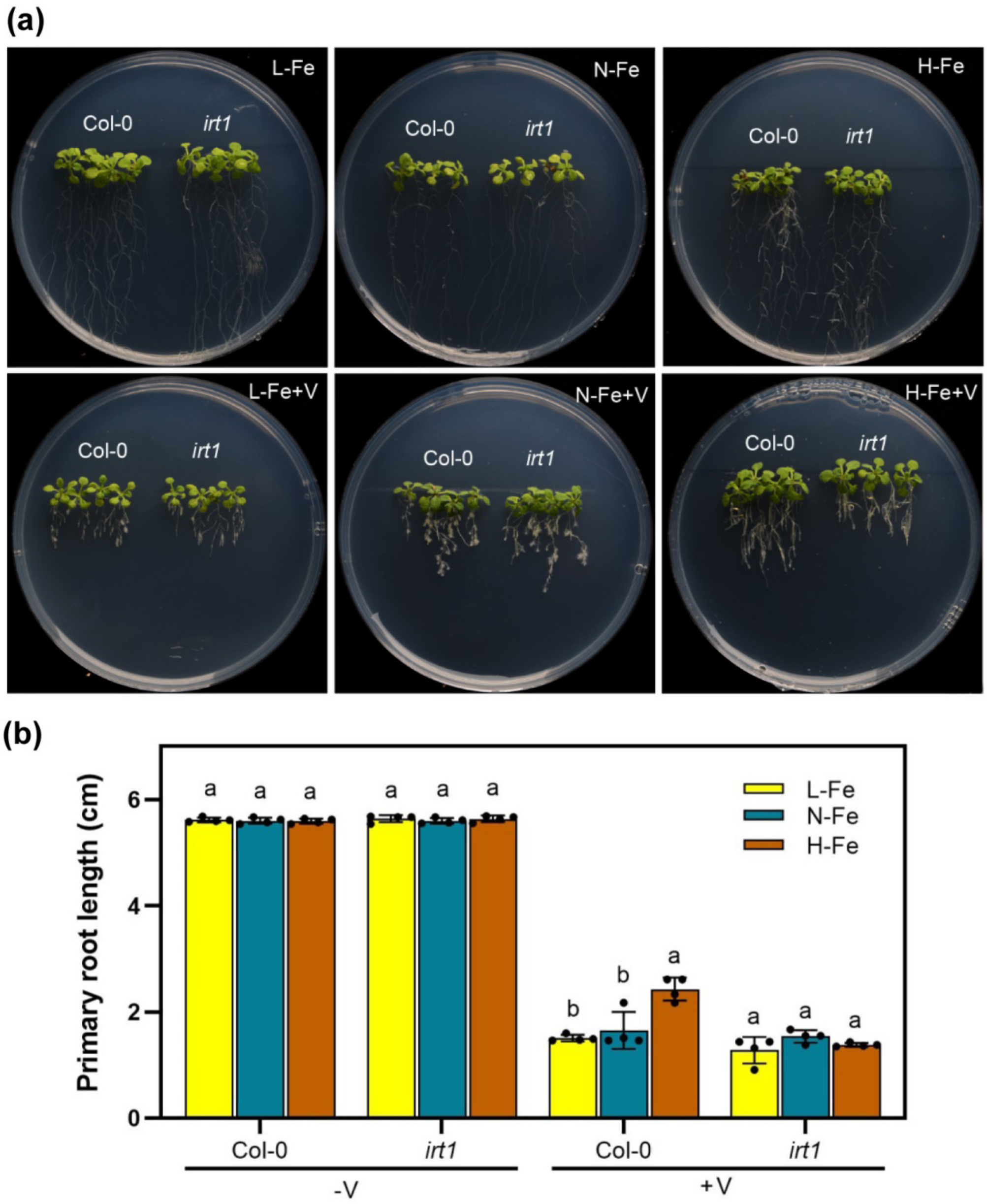

Figure S18. Growth phenotypes of *Arabidopsis thaliana* iron (Fe) transporter mutant *irt1* under different concentrations of vanadium (V) and Fe.

(a) Growth phenotype of 2-week-old wild-type (Col-0) and *irt1* mutant plants under different V and Fe conditions. Seedlings were cultured on ANS agar medium with (+V) or without (-V) 5mg L^{-1} VO_4^{3-}, accompanied by low iron (L-Fe, 8.96 µM Fe^{2+}), normal iron (N-Fe, 44.8 µM Fe^{2+}), and high iron (H-Fe, 89.6 µM Fe^{2+}) concentrations.

(b) Primary root length of wild-type (Col-0) and *irt1* seedlings under different P and V conditions as described in (a). The data are expressed as mean ± SD. $n = 4$. Different letters above the bars represent significant differences at $P < 0.05$ (Duncan's test).

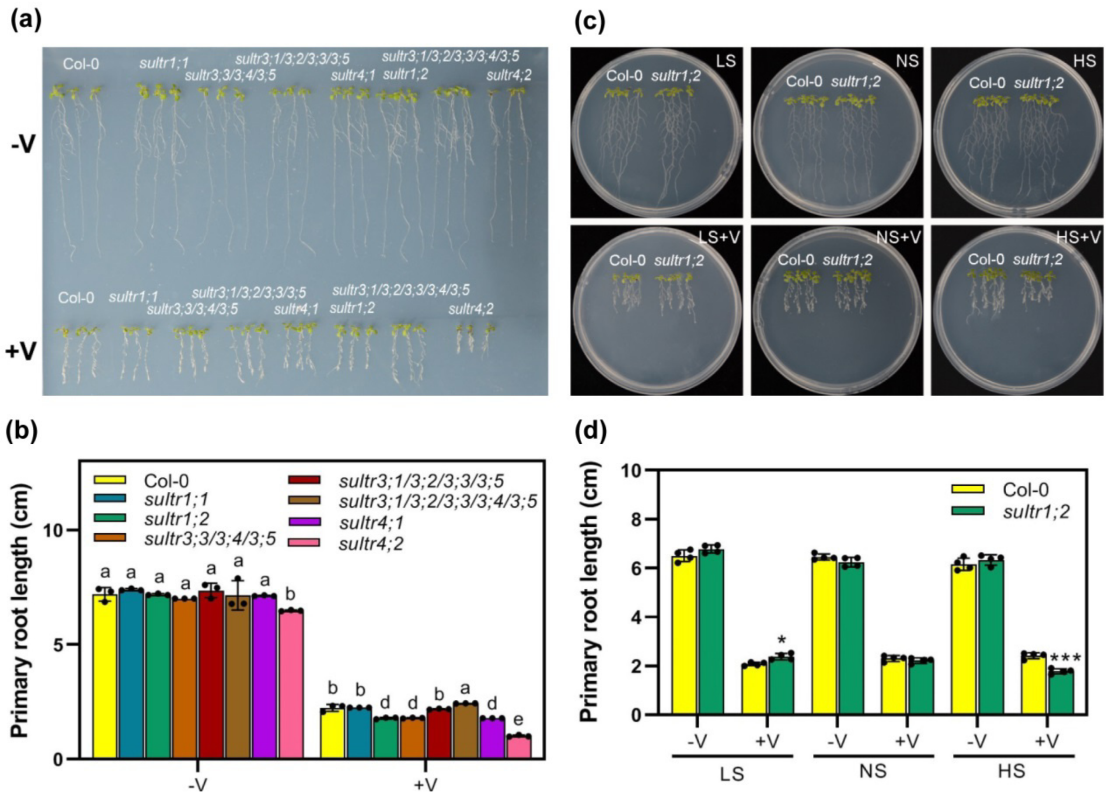

Figure S19. Growth phenotypes of *Arabidopsis thaliana* wild-type (Col-0) and sulfur (S) transporter mutants under different concentrations of vanadium (V) and S.

(a) Growth Phenotypes of two-week-old wild-type (Col-0) and S transporter mutant (*sultr1;1*, *sultr1;2*, *sultr3;3/3;4/3;5*, *sultr3;1/3;2/3;3/3;5*, *sultr3;1/3;2/3;3/3;4/3;5*, *sultr4;1*, and *sultr4;2*) plants under V stress. Seedlings were cultured in ANS agar medium with (+V) or without (-V) 5 mg L^{-1} VO_4^{3-}.

(b) Primary root length of wild-type (Col-0) and S transporter mutants under V stress as described in (a).

(c) Growth phenotypes of two-week-old wild-type (Col-0) and *sultr1;2* mutant plants under different V and S conditions. Seedlings were cultured in ANS agar medium containing 5000 μM (HS), 500 μM (NS) or 50 μM (LS) SO_4^{2-}, and 0 (-V) or 5mg L^{-1} VO_4^{3-} (+V).

(d) Primary root length of wild-type (Col-0) and *sultr1;2* mutant plants under different V and S conditions as described in (c).

Data are expressed as mean ± SD. n = 4. Different letters above the bars represent significant differences at P < 0.05 (Duncan's test). Asterisks denote statistically different values according to Duncan's test (*** P < 0.001).

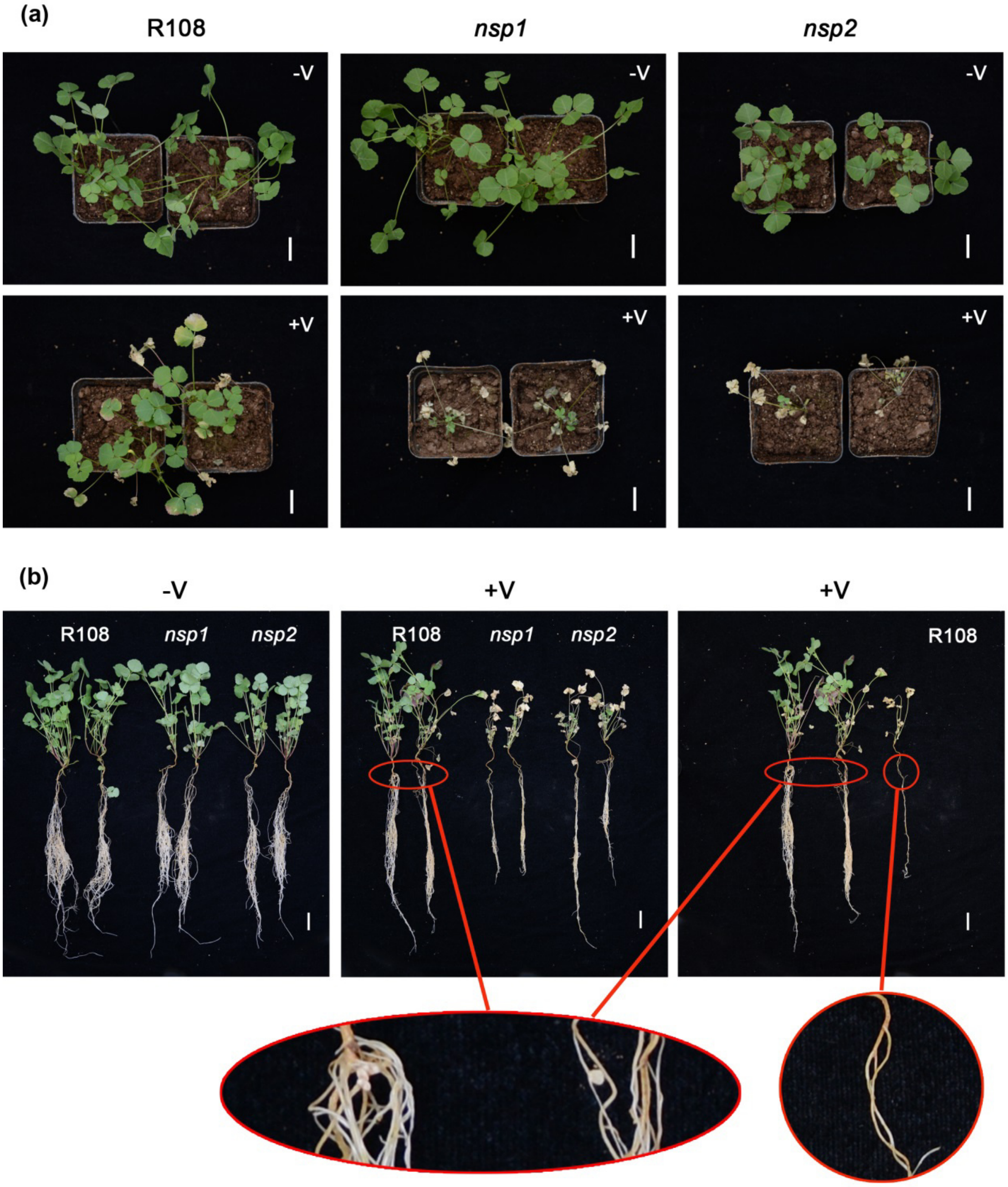

Figure S20. Soil autoclave was not able to completely eliminate the impact of microorganisms on plant vanadium (V) tolerance.

(a) Some wild-type (R108) plants was still more tolerant to V stress than *nsp1* and *nsp2* mutants under autoclaved soil growth conditions. Three-week-old seedlings were treated with (+V) or without (-V) 500 mg L^{-1} VO_4^{3-} for one week. Bars, 2 cm.

(b) Wild-type (R108) plants with nodules had higher V tolerance than *nsp1*, *nsp2*, and R108 without nodules. Seedlings were treated as described in (a). The plants with nodules circled by red boxes in the middle and right images were the same plants. Bars, 2 cm.

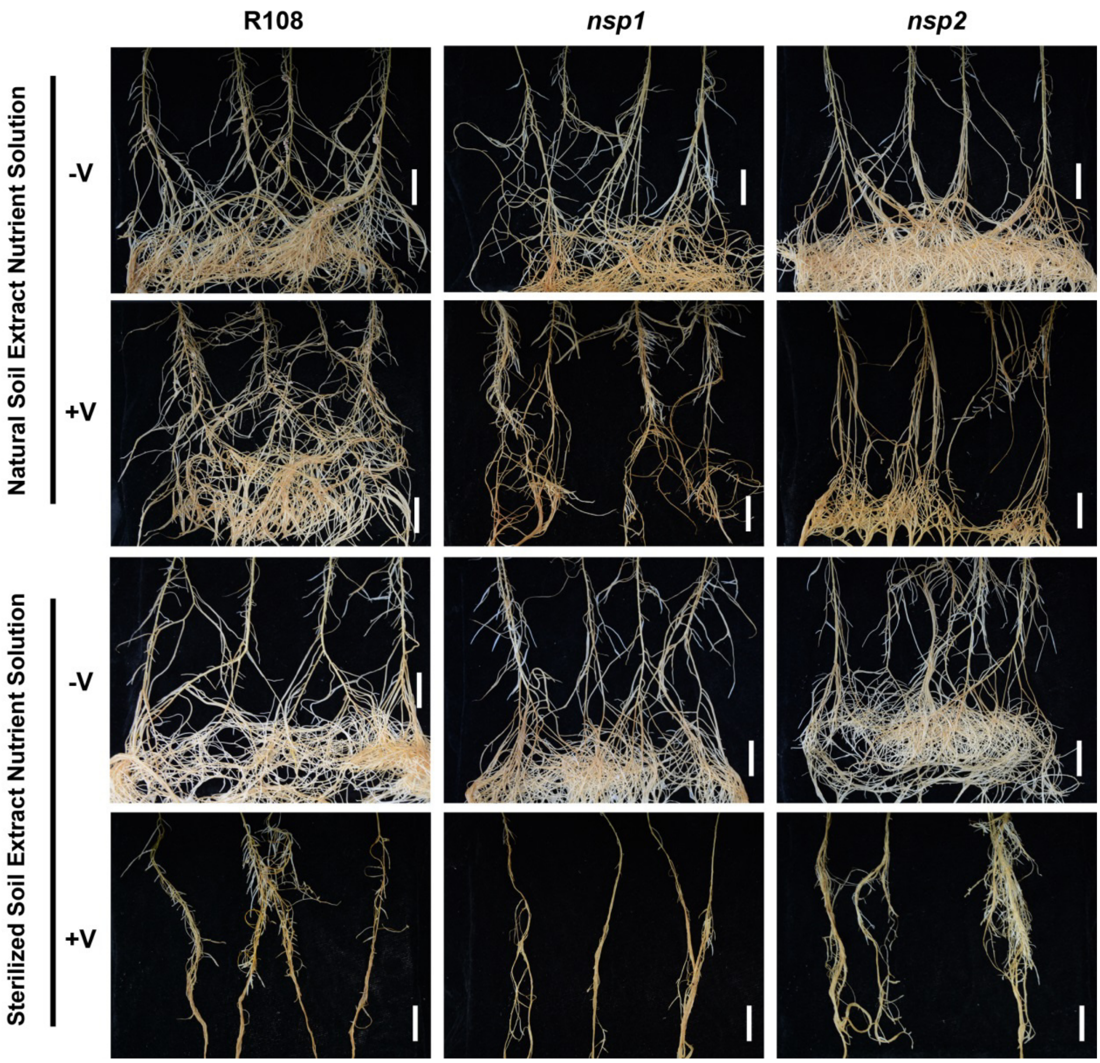

Figure S21. Root phenotypes of wild-type (R108) and *nsp1* and *nsp2* mutant *Medicago truncatula* plants under vanadium (V) stress in the presence and absence of soil microorganisms.

Three-week-old seedlings were cultured with ½-strength Hoagland nutrient solution prepared with non-sterile (Natural) or sterile soil extract (Sterilized Soil Extract Nutrient Solution). After nodulation of R108, plants were treated with (+V) or without (-V) 30 mg L^{-1} VO_4^{3-} for one week. The roots in images correspond to the shoots shown in Figure 5. Bars, 2 cm.

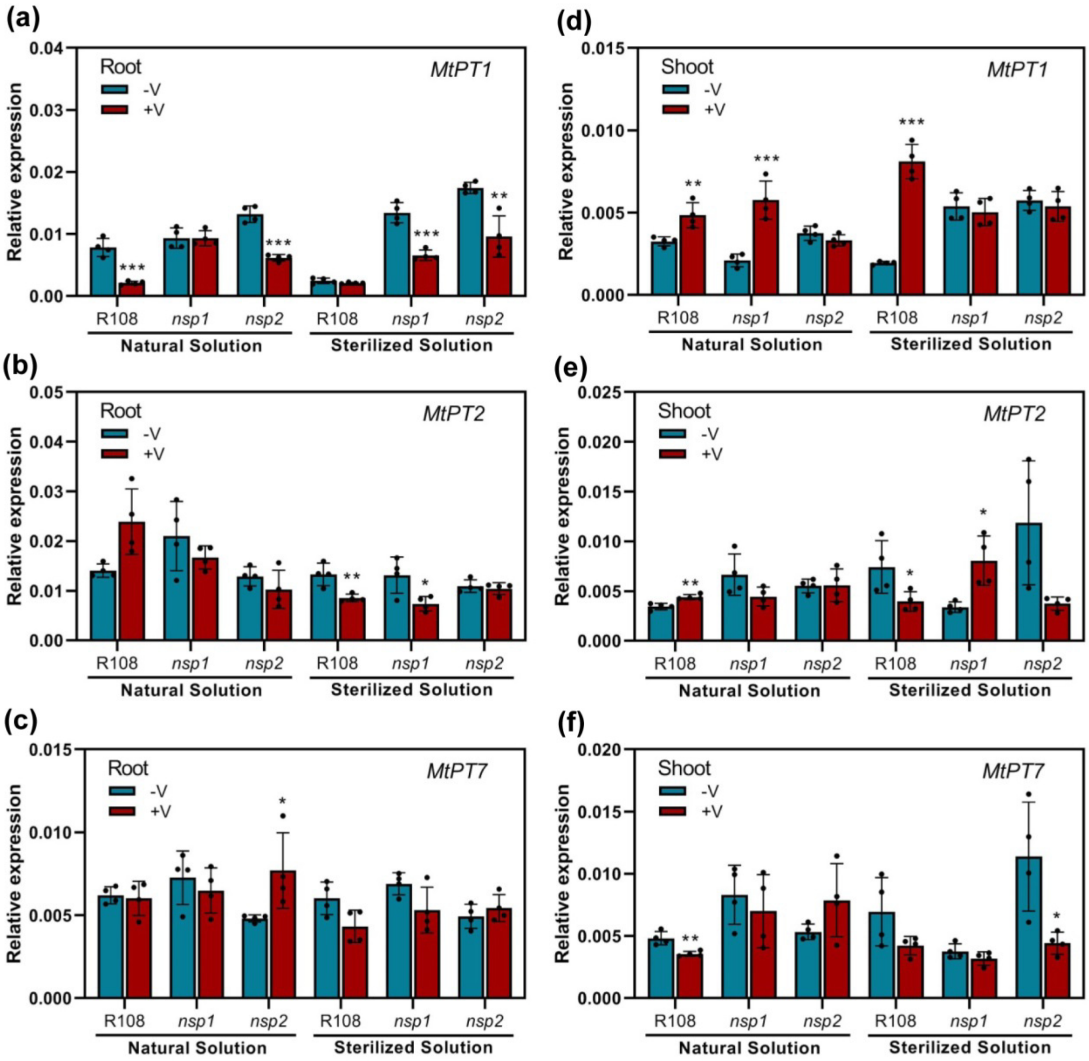

Figure S22. Relative expressions of *PHT1* family phosphate transporter genes in the roots and shoots of *Medicago truncatula* wild-type (R108), *nsp1*, and *nsp2* mutant plants under vanadium (V) stress.

(a–c) The relative expression of *MtPT1* (a), *MtPT2* (b), and *MtPT7* (c) in roots of wild-type (R108), *nsp1*, and *nsp2* plants. Three-week-old seedlings were cultured with ½-strength Hoagland nutrient solution prepared with non-sterile (Natural) or sterile soil extract (Sterilized Solution). After nodulation of R108, plants were treated with (+V) or without (-V) 30 mg L^{-1} VO_4^{3-} for one week, and used for mRNA analysis.

(d–f) The relative expression of *MtPT1* (d), *MtPT2* (e), and *MtPT7* (f) in shoots of wild-type (R108), *nsp1*, and *nsp2* plants. Seedlings were treated as described in (a–c). Data are expressed as mean ± SD (n = 4). Asterisks denote statistically significant differences according to Duncan's test (* $P < 0.05$, ** $P < 0.01$, *** $P < 0.001$).

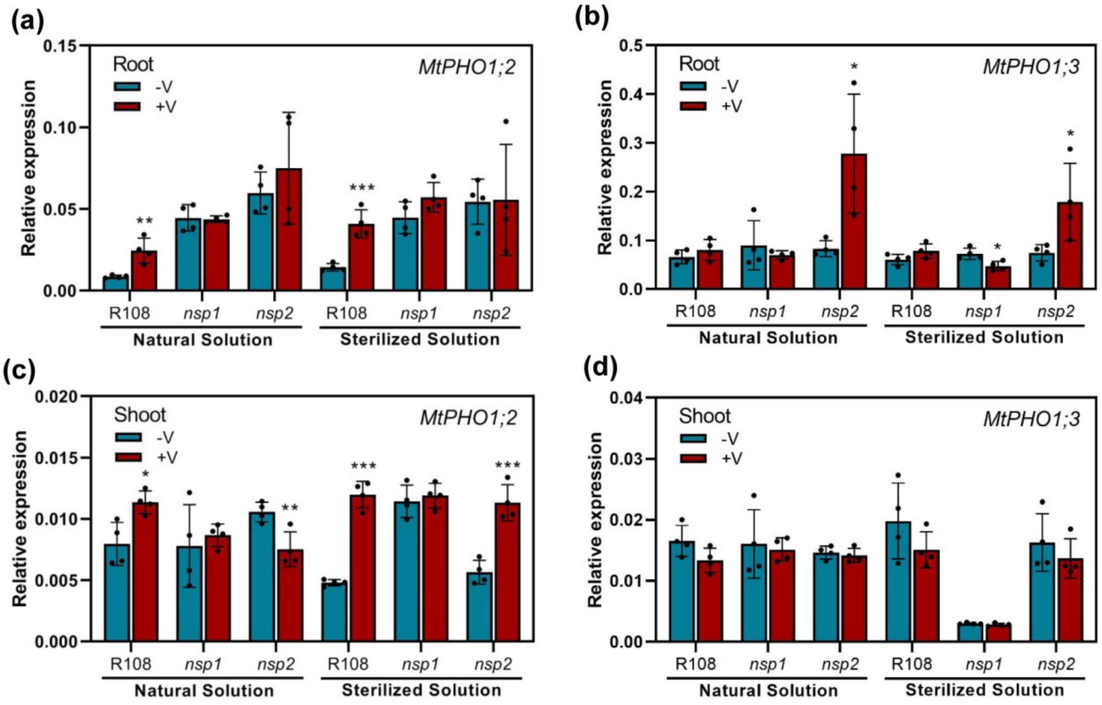

Figure S23. Relative expressions of *PHO1* family phosphate transporter genes in the roots and shoots of *Medicago truncatula* wild-type (R108), *nsp1*, and *nsp2* mutant plants under vanadium (V) stress.

(a,b) The relative expression of *MtPHO1;2* (a) and *MtPHO1;3* (b) in roots of wild-type (R108), *nsp1*, and *nsp2* plants. Three-week-old seedlings were cultured with ½-strength Hoagland nutrient solution prepared with non-sterile (Natural) or sterile soil extract (Sterilized Solution). After nodulation of R108, plants were treated with (+V) or without (-V) 30 mg L^{-1} VO_4^{3-} for one week, and used for mRNA analysis.

(c,d) The relative expression of *MtPHO1;2* (c) and *MtPHO1;3* (d) in shoots of wild-type (R108), *nsp1*, and *nsp2* plants. Seedlings were treated as described in (a,b). Data are expressed as mean ± SD (n = 4). Asterisks denote statistically significant differences according to Duncan's test (* $P < 0.05$, ** $P < 0.01$, *** $P < 0.001$).

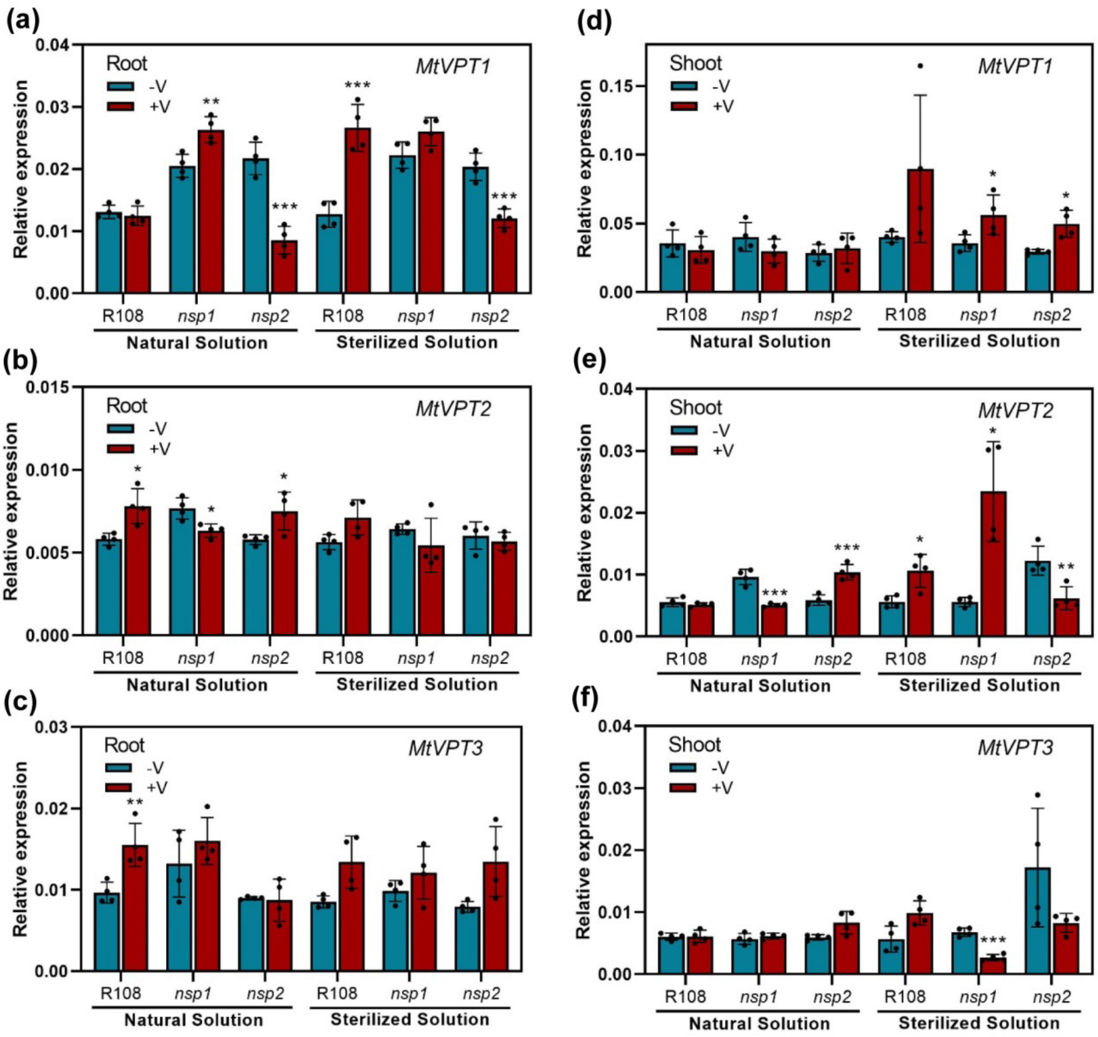

Figure S24. Relative expressions of *VPTs* family phosphate transporter genes in the roots and shoots of *Medicago truncatula* wild-type (R108), *nsp1*, and *nsp2* mutant plants under vanadium (V) stress.

(a–c) The relative expression of *MtVPT1* (a), *MtVPT2* (b), and *MtVPT3* (c) in roots of wild-type (R108), *nsp1*, and *nsp2* plants. Three-week-old seedlings were cultured with ½-strength Hoagland nutrient solution prepared with non-sterile (Natural) or sterile soil extract (Sterilized Solution). After nodulation of R108, plants were treated with (+V) or without (-V) 30 mg L^{-1} VO_4^{3-} for one week, and used for mRNA analysis.

(d–f) The relative expression levels of *MtVPT1*(d), *MtVPT2* (e), and *MtVPT3* (f) in shoots of wild-type (R108), *nsp1*, and *nsp2* plants. Seedlings were treated as described in (a–c). Data are expressed as mean ± SD (n = 4). Asterisks denote statistically significant differences according to Duncan's test (* $P < 0.05$, ** $P < 0.01$, *** $P <$ 0.001).

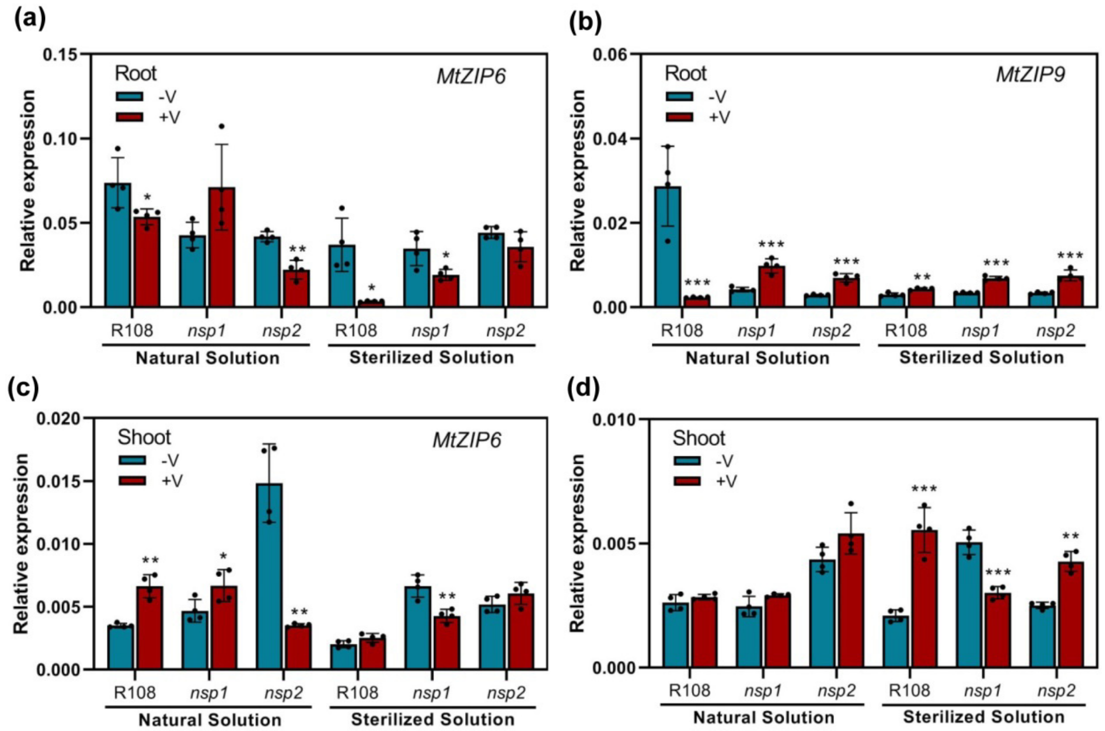

Figure S25. Relative expressions of *ZIP* family iron transporter genes in the roots and shoots of *Medicago truncatula* wild-type (R108), *nsp1*, and *nsp2* mutant plants under vanadium (V) stress.

(a,b) The relative expression of *MtZIP6* (a) and *MtZIP9* (b) in roots of wild-type (R108), *nsp1*, and *nsp2* plants. Three-week-old seedlings were cultured with ½-strength Hoagland nutrient solution prepared with non-sterile (Natural) or sterile soil extract (Sterilized Solution). After nodulation of R108, plants were treated with (+V) or without (-V) 30 mg L^{-1} VO_4^{3-} for one week, and used for mRNA analysis.

(c,d) The relative expression of *MtZIP6* (c) and *MtZIP9* (d) in shoots of wild-type (R108), *nsp1*, and *nsp2* plants. Seedlings were treated as described in (a,b). Data are expressed as mean ± SD (n = 4). Asterisks denote statistically significant differences according to Duncan's test (* $P < 0.05$, ** $P < 0.01$, *** $P < 0.001$).

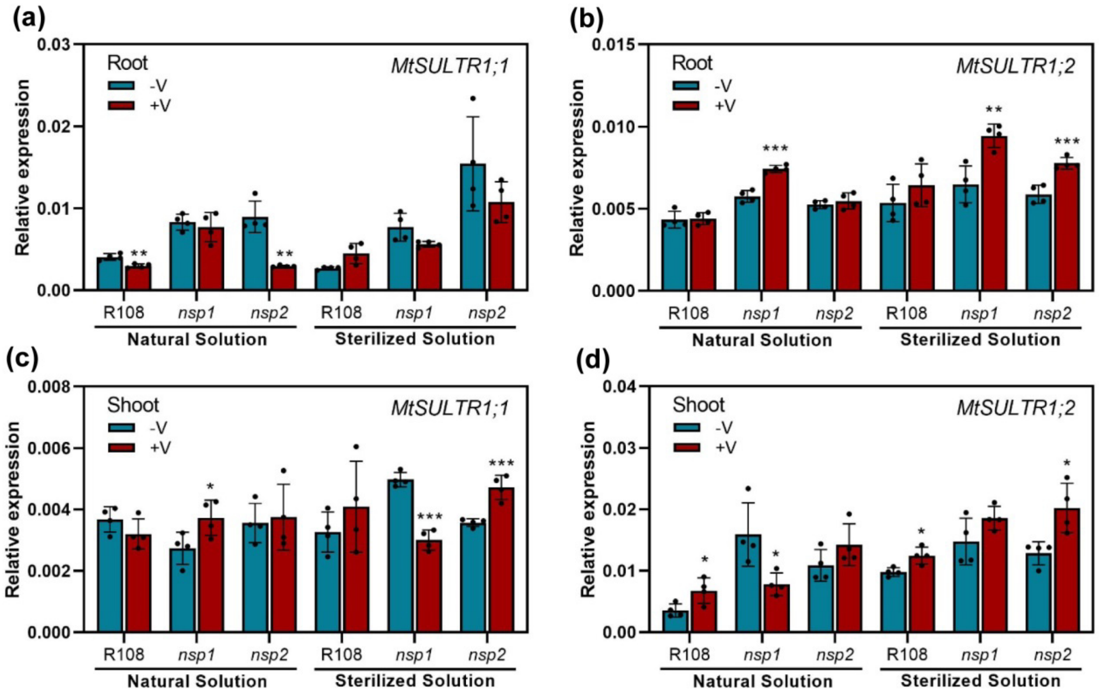

Figure S26. Relative expressions of *SULTR1* family sulfur transporter genes in the roots and shoots of *Medicago truncatula* wild-type (R108), *nsp1*, and *nsp2* mutant plants under vanadium (V) stress.

(a,b) The relative expression of *MtSULTR1;1* (a) and *MtSULTR1;2* (b) in roots of wild-type (R108), *nsp1*, and *nsp2* plants. Three-week-old seedlings were cultured with ½-strength Hoagland nutrient solution prepared with non-sterile (Natural) or sterile soil extract (Sterilized Solution). After nodulation of R108, plants were treated with (+V) or without (-V) 30 mg L^{-1} VO_4^{3-} for one week, and used for mRNA analysis.

(c,d) The relative expression of *MtSULTR1;1* (c) and *MtSULTR1;2* (d) in shoots of wild-type (R108), *nsp1*, and *nsp2* plants. Seedlings were treated as described in (a,b). Data are expressed as mean ± SD (n = 4). Asterisks denote statistically significant differences according to Duncan's test (* $P < 0.05$, ** $P < 0.01$, *** $P < 0.001$).

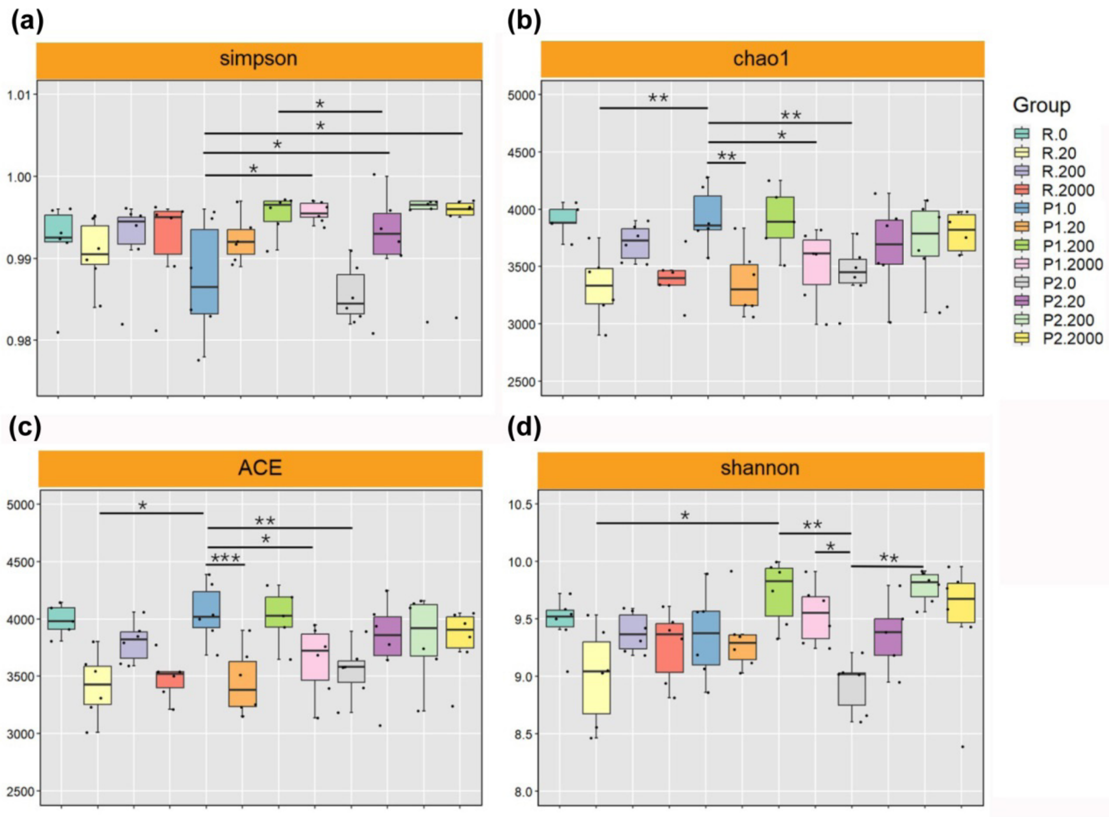

Figure S27. *NSP1* and *NSP2* mutations alter the α-diversity of rhizobacteria under vanadium (V) stress.

Simpson, chao1, ACE, and Shannon indices were used to depict the α-diversity of rhizobacteria in wild-type (R108), *nsp1*, and *nsp2 Medicago truncatula* plants treated with different V concentrations. The points represent sample values, and the box plots display quartile values. In the legend, R, P1, and P2 represent R108, *nsp1*, and *nsp2*, respectively, while 0, 20, 200, and 2000 represent VO_4^{3-} treatment concentrations in mg L^{-1}. Asterisks indicate statistically significant differences according to Wilcoxon rank-sum test and Tukey's test (* $P < 0.05$, ** $P < 0.01$, *** $P < 0.001$).

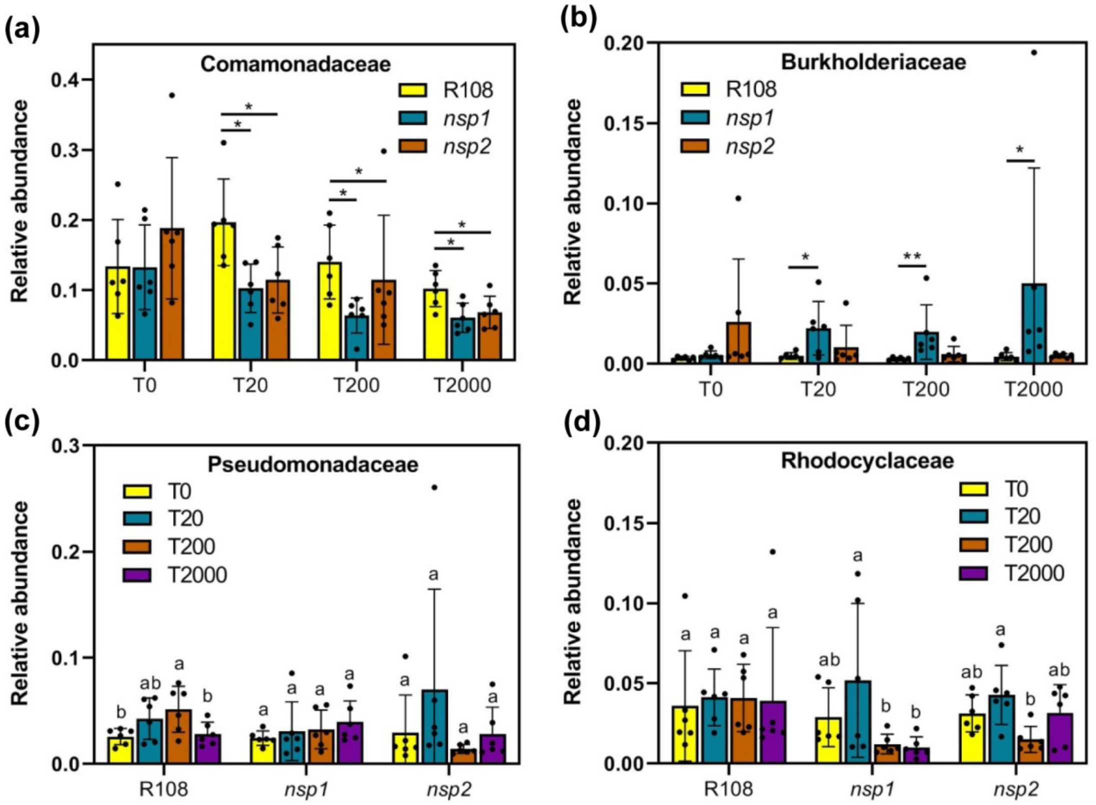

Figure S28. Effects of *NSP1* and *NSP2* mutations on the relative abundance of rhizobacteria under vanadium (V) stress.

(a,b) Relative abundances of Comamonadaceae (a) and Burkholderiaceae (b) in the rhizosphere soil of wild-type (R108), *nsp1*, and *nsp2* mutant plants under different V conditions. Four-week-old seedlings were treated with 0 mg L^{-1} (T0), 20 mg L^{-1} (T20), 200 mg L^{-1} (T200), and 2000 mg L^{-1} (T2000) VO_4^{3-} for one week. Asterisks indicate statistically significant differences according to Duncan's test (* $P < 0.05$, ** $P < 0.01$, *** $P < 0.001$).

(c,d) Relative abundances of Pseudomonadaceae (c) and Rhodocyclaceae (d) in the rhizosphere soil of wild-type (R108), *nsp1*, and *nsp2* plants under different V conditions. Plants were treated as described in (a,b). Different letters above the bars represent significant differences at $P < 0.05$ (Duncan's test).

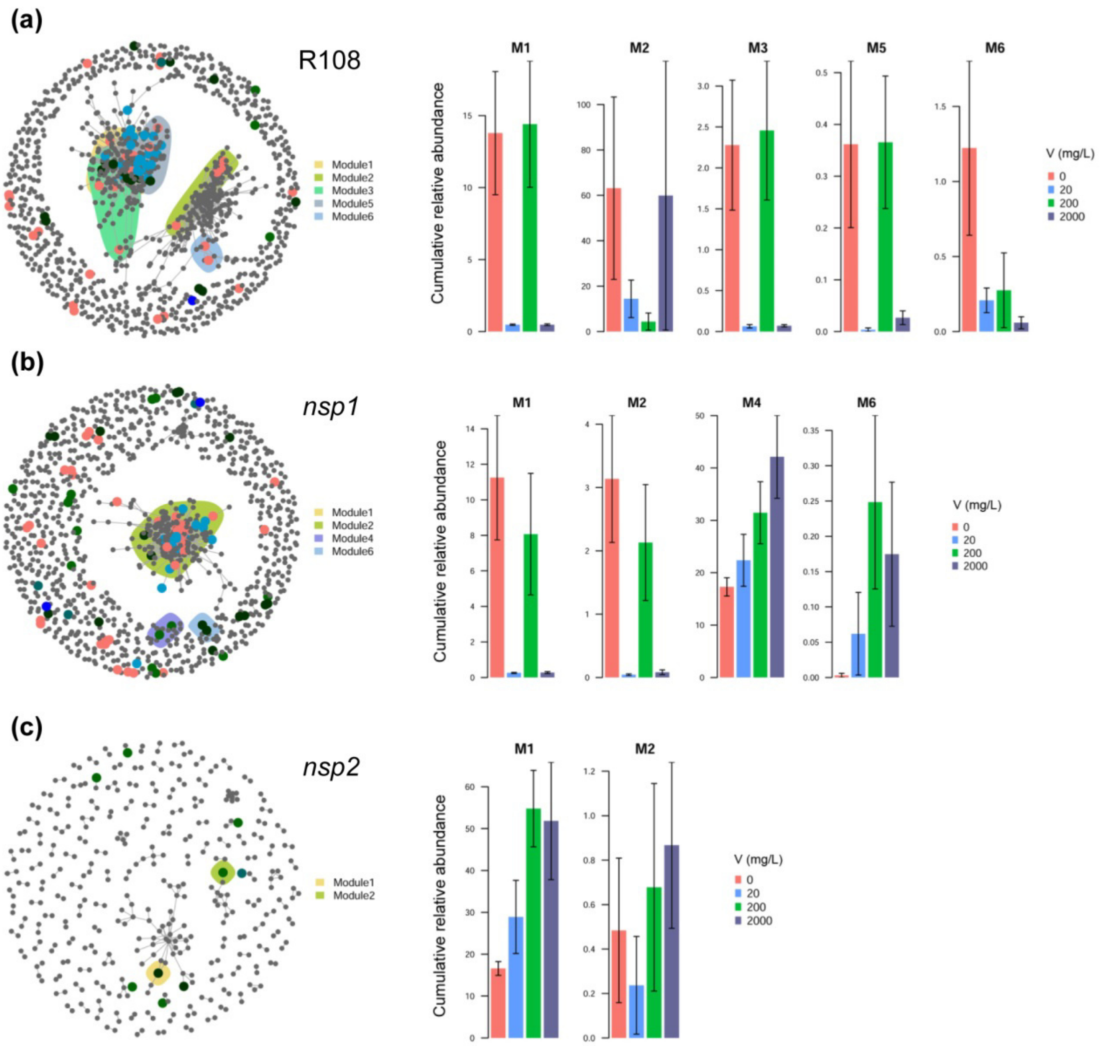

Figure S29. The distribution of vanadium-responsive operational taxonomic units (VrOTUs) in wild-type (R108), *nsp1*, and *nsp2* mutant plants.

(a) Wild-type (R108) rhizobacterial co-occurrence network and modules containing VrOTUs. The co-occurrence network is the same as shown in Figure 8. The bar chart displays the cumulative relative abundance of VrOTUs (in millions) under 0 mg L^{-1} (pink), 20 mg L^{-1} (baby blue), 200 mg L^{-1} (green), and 2000 mg L^{-1} (dark gray) VO_4^{3-} treatments.

(b) The rhizobacterial co-occurrence networks and VrOTUs modules for *nsp1*. The bar chart was displayed in the same manner as described in (a).

(c) The rhizobacterial co-occurrence networks and VrOTUs modules for *nsp2*. The bar chart was displayed in the same manner as described in (a).

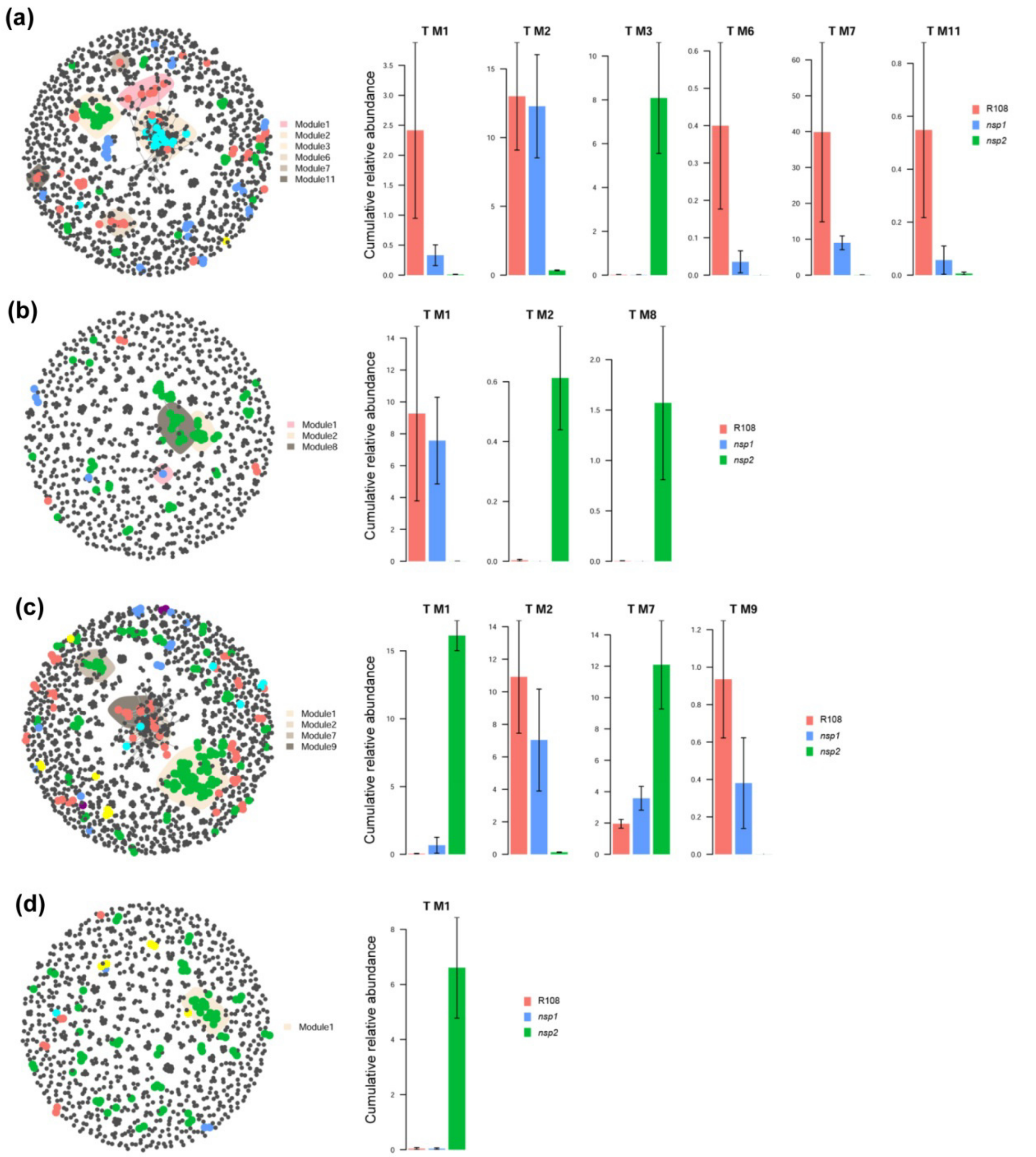

Figure S30. Species-differentiated operational taxonomic units (SdOTUs) distribution of rhizobacteria from wild-type (R108), *nps1*, and *nsp2* plants under the same vanadium concentration treatment.

(a) The rhizobacterial co-occurrence network and modules containing SdOTUs under 0 mg L^{-1} VO_4^{3-} treatment. Low frequency operational taxonomic units (OTUs) with frequencies less than 83% were removed from all samples. SdOTUs were highlighted and colored. The edges of the co-occurrence network were selected based on Spearman's rho > 0.7 and *P*-value < 0.001. The bar chart displays the cumulative relative abundance of SdOTUs (in millions) in R108 (pink), *nsp1* (baby blue), and *nsp2* (green). The cumulative relative abundance reflects the significant influence of plant materials on the SdOTUs.

(b) The rhizobacterial co-occurrence network and modules containing SdOTUs under 20 mg L^{-1} VO_4^{3-} treatment. Data were analyzed as described in (a).

(c) The rhizobacterial co-occurrence network and modules containing SdOTUs under 200 mg L^{-1} VO_4^{3-} treatment. Data were analyzed as described in (a).

(d) The rhizobacterial co-occurrence network and modules containing SdOTUs under 2000 mg L^{-1} VO_4^{3-} treatment. Data were analyzed as described in (a).

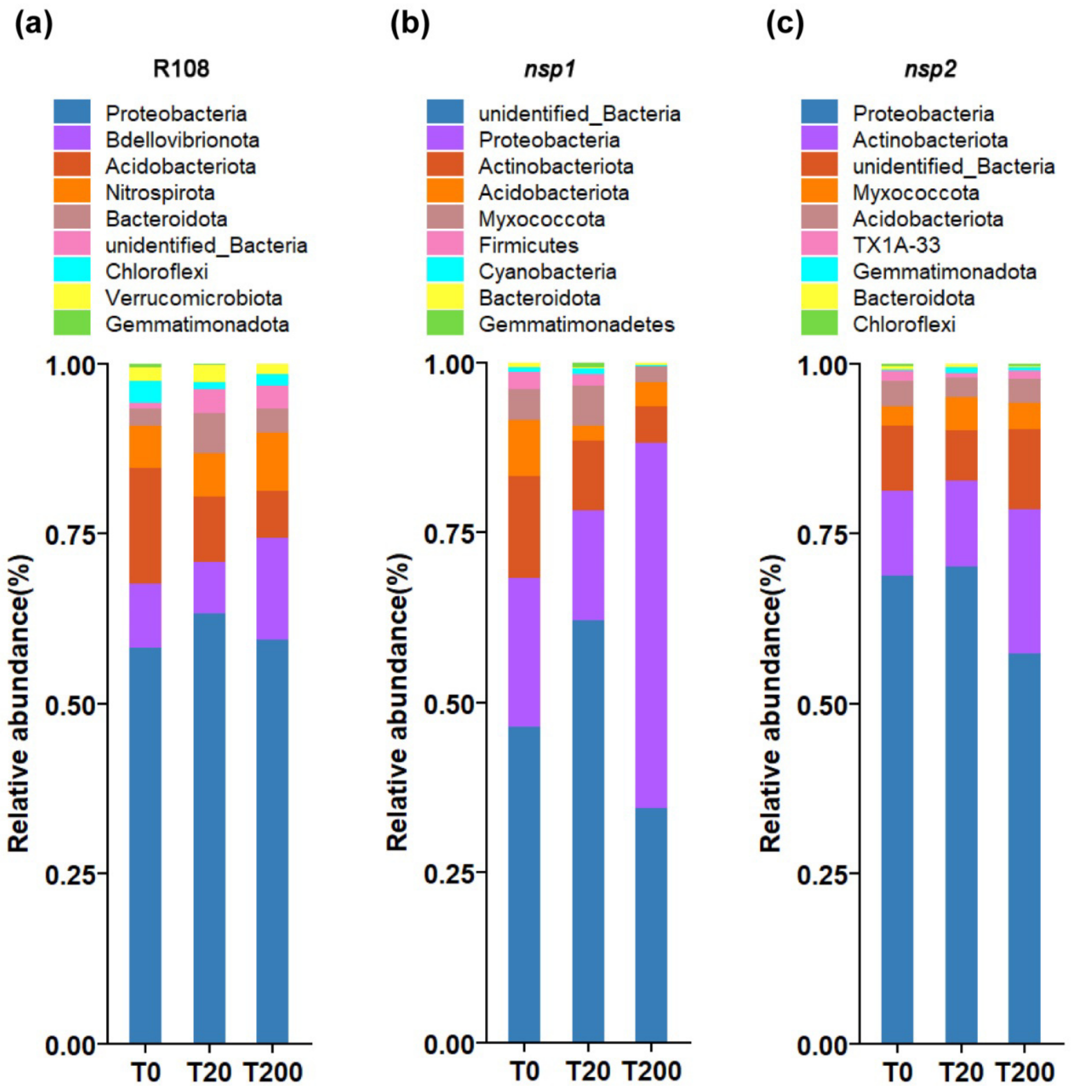

Figure S31. Relative abundance accumulation of rhizobacterial biomarkers in wild-type (R108), *nsp1*, and *nsp2* plants under vanadium (V) stress.

(a) The relative abundance accumulation of rhizobacterial biomarkers in wild-type (R108) plants under different V conditions. Four-week-old seedlings were treated with 0 mg L^{-1} (T0), 20 mg L^{-1} (T20), 200 mg L^{-1} (T200), and 2000 mg L^{-1} (T2000) VO_4^{3-} for one week.

(b) The relative abundance accumulation of rhizobacterial biomarkers in *nsp1* plants under different V conditions. Seedlings were treated as described in (a).

(c) The relative abundance accumulation of rhizobacterial biomarkers in *nsp2* plants under different V conditions. Seedlings were treated as described in (a).

3. Supporting Tables

Table S1. List of rhizobacterial biomarkers classification

R108		*nsp1*		*nsp2*	
**OTUID**	Phylum	OTUID	Phylum	OTUID	Phylum
**OTU772**	unidentified_Bacteria	OTU8312	Acidobacteriota	OTU8602	Actinobacteriota
**OTU6607**	Proteobacteria	OTU81	unidentified_Bacteria	OTU85	Proteobacteria
**OTU605**	Verrucomicrobiota	OTU806	Cyanobacteria	OTU753	Actinobacteriota
**OTU5925**	Proteobacteria	OTU80	Proteobacteria	OTU5772	Bacteroidota
**OTU533**	Proteobacteria	OTU7730	unidentified_Bacteria	OTU439	unidentified_Bacteria
**OTU4595**	Nitrospirota	OTU753	Actinobacteriota	OTU4040	unidentified_Bacteria
**OTU4166**	Gemmatimonadota	OTU6445	unidentified_Bacteria	OTU3737	unidentified_Bacteria
**OTU3606**	Bdellovibrionota	OTU5925	Proteobacteria	OTU358	Acidobacteriota
**OTU322**	Proteobacteria	OTU4935	Proteobacteria	OTU3157	Bacteroidota
**OTU3058**	unidentified_Bacteria	OTU380	Firmicutes	OTU310	Myxococcota
**OTU244**	Bdellovibrionota	OTU3585	Acidobacteriota	OTU2884	Proteobacteria
**OTU1908**	Proteobacteria	OTU331	unidentified_Bacteria	OTU268	Proteobacteria
**OTU1819**	Bacteroidota	OTU2400	Bacteroidota	OTU2277	Acidobacteriota
**OTU1515**	Verrucomicrobiota	OTU200	Myxococcota	OTU2198	Gemmatimonadota
**OTU1514**	Chloroflexi	OTU1796	Myxococcota	OTU1897	Actinobacteriota
**OTU1372**	Chloroflexi	OTU1672	unidentified_Bacteria	OTU1517	Proteobacteria
**OTU13592**	unidentified_Bacteria	OTU1636	Gemmatimonadetes	OTU1475	Chloroflexi
**OTU1268**	unidentified_Bacteria	OTU1485	unidentified_Bacteria	OTU13558	TX1A-33
**OTU1228**	Acidobacteriota	OTU11678	Proteobacteria	OTU1214	unidentified_Bacteria
**OTU10008**	Proteobacteria	OTU1096	Proteobacteria	OTU11891	Proteobacteria

Table S2. List of PCR primers

Names	Genes	Locus tag	Purposes	Primer sequences (5'-3')
Tnt-F	*Tnt1*		Mutant identification	TCCTTGTTGGATTGGTAGCCAACTTTGTTG
Tnt-R				TGTAGCACCGAGATACGGTAATTAACAAGA
nsp1-NF9220-F	*NSP1*	MTR_8g020840		GTCTCTTTCTTTCCATCATTTTTG
nsp1-NF9220-R				GGCTCTTCCACCATAGTTCC
nsp2-NF10950-F	*NSP2*	MTR_3g072710		ATGCCATCAATGACCTCCACT
nsp2-NF10950-R				TATTACTACCCACACCCACCTCTT
MtPT1-qPCR-F	*MtPT1*	MTR_1g043220	RT-qPCR	TGGCAAATCATAGTCGCATC
MtPT1-qPCR-R				TGACATTTGTGAGGCTGAGG
MtPT2-qPCR-F	*MtPT2*	MTR_1g043290		TGTTAGGTAAAGCCCGTGTTACT
MtPT2-qPCR-R				ACGATGTTCCACACTTGGCT
MtPT7-qPCR-F	*MtPT7*	MTR_1g074940		TGTGGCCAATTTCTTGGGGA
MtPT7-qPCR-R				CAACTTCAATGCCTAGTTTCTTCC
MtPHO1;2-qPCR-F	*MtPHO1;2*	MTR_1g075640		GGTGACATGGGTTGAGACTATC
MtPHO1;2-qPCR-R				CAGTGCCCTCTTCGAATAACT
MtPHO1;3-qPCR-F	*MtPHO1;3*	MTR_8g069955		GCGGATCAACTTTGCAGTTAG
MtPHO1;3-qPCR-R				CCCTCATGCAGTATCCATAGTC
MtVPT1-qPCR-F	*MtVPT1*	MTR_6g053510		GTGGGAATAGGTGCTGTTGT
MtVPT1-qPCR-R				GCAGGCTGATCGTAGATTGATAG
MtVPT2-qPCR-F	*MtVPT2*	MTR_1g099580		CGTGTTATGTCATCAAGGCTTTC
MtVPT2-qPCR-R				CAAGTAGCCAGCAAGGGTAATA
MtVPT3-qPCR-F	*MtVPT3*	MTR_7g076520		GGCTTGTGGTCCAGCTATT
MtVPT3-qPCR-R				CCATCCAGGTAGTGTGTCTTG
MtZIP9-qPCR-F	*MtZIP9*	MTR_3g081640		CATACTCATGCATCCCATGGTC
MtZIP9-qPCR-R				CTACAAGCGGCCTTATGGT
MtZIP6-qPCR-F	*MtZIP6*	MTR_4g083570		GGACATTCACATGGTGGTCAC
MtZIP6-qPCR-R				GCTCCCATTCCAAGCCCTATC
MtSULTR1;1-qPCR-F	*MtSULTR1;1*	MTR_2g008470		GATTGCTGGACTCACCATTGC
MtSULTR1;1-qPCR-R				TTCCAAGCAAGAGAGACACCAC
MtSULTR1;2-qPCR-F	*MtSULTR1;2*	MTR_3g073780		GCTGGTCTCACTATTGCAAGTC
MtSULTR1;2-qPCR-R				CGGTCCTATAGCTATATCGCGAG
MtACTIN-F	*MtACTIN*	MTR_3g095530		TTGCTGACCGTATGAGCAAG
MtACTIN-R				TGGATGGACCAGACTCATCA
